# Highly Sensitive Ammonia Gas Sensors at Room Temperature Based on the Catalytic Mechanism of N, C Coordinated Ni Single-Atom Active Center

**DOI:** 10.1007/s40820-024-01484-4

**Published:** 2024-08-27

**Authors:** Wenjing Quan, Jia Shi, Min Zeng, Wen Lv, Xiyu Chen, Chao Fan, Yongwei Zhang, Zhou Liu, Xiaolu Huang, Jianhua Yang, Nantao Hu, Tao Wang, Zhi Yang

**Affiliations:** 1 National Key Laboratory of Advanced Micro and Nano Manufacture Technology, Shanghai Jiao Tong University, Shanghai, 200240 People’s Republic of China; 2 Department of Micro/Nano Electronics, School of Electronic Information and Electrical Engineering, Shanghai Jiao Tong University, Shanghai, 200240 People’s Republic of China; 3 Shanghai Key Laboratory of Intelligent Sensing and Detection Technology, School of Mechanical and Power Engineering, East China University of Science and Technology, Shanghai, 200237 People’s Republic of China

**Keywords:** Gas sensor, Single atom, Catalytic activation, Targeted adsorption, End-sealing passivation

## Abstract

Significant challenges are posed by the limitations of gas sensing mechanisms for trace-level detection of ammonia (NH_3_). In this study, we propose to exploit single-atom catalytic activation and targeted adsorption properties to achieve highly sensitive and selective NH_3_ gas detection. Specifically, Ni single-atom active sites based on N, C coordination (Ni–N–C) were interfacially confined on the surface of two-dimensional (2D) MXene nanosheets (Ni–N–C/Ti_3_C_2_T_x_), and a fully flexible gas sensor (MNPE–Ni–N–C/Ti_3_C_2_T_x_) was integrated. The sensor demonstrates a remarkable response value to 5 ppm NH_3_ (27.3%), excellent selectivity for NH_3_, and a low theoretical detection limit of 12.1 ppb. Simulation analysis by density functional calculation reveals that the Ni single-atom center with N, C coordination exhibits specific targeted adsorption properties for NH_3_. Additionally, its catalytic activation effect effectively reduces the Gibbs free energy of the sensing elemental reaction, while its electronic structure promotes the spill-over effect of reactive oxygen species at the gas–solid interface. The sensor has a dual-channel sensing mechanism of both chemical and electronic sensitization, which facilitates efficient electron transfer to the 2D MXene conductive network, resulting in the formation of the NH_3_ gas molecule sensing signal. Furthermore, the passivation of MXene edge defects by a conjugated hydrogen bond network enhances the long-term stability of MXene-based electrodes under high humidity conditions. This work achieves highly sensitive room-temperature NH_3_ gas detection based on the catalytic mechanism of Ni single-atom active center with N, C coordination, which provides a novel gas sensing mechanism for room-temperature trace gas detection research.

**Supplementary Information:**

The online version contains supplementary material available at 10.1007/s40820-024-01484-4.

## Highlights

- Exploiting single-atom catalytic activation and targeted adsorption properties, Ni single-atom active sites based on N, C coordination are constructed on the surface of two-dimensional MXene nanosheets (Ni–N–C/Ti_3_C_2_T_x_), enabling highly sensitive and selective NH_3_ gas detection.
- The catalytic activation effect of Ni–N–C/Ti_3_C_2_T_x_ effectively reduces the Gibbs free energy of the sensing elemental reaction, while its electronic structure promotes the spill-over effect of reactive oxygen species at the gas–solid interface.
- An end-sealing passivation strategy utilizing a conjugated hydrogen bond network of the conductive polymer was employed on MXene-based flexible electrodes, effectively mitigating the oxidative degradation of MXene-based gas sensors.

**Supplementary Information:**

The online version contains supplementary material available at 10.1007/s40820-024-01484-4.

## Graphical abstract

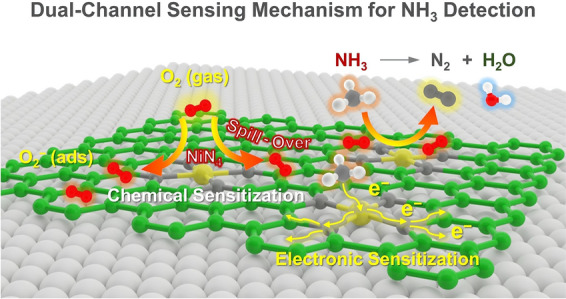

## Introduction

Early detection of variations in exhaled breath levels can be crucial in the prevention and early intervention of certain medical conditions. For instance, the concentration of exhaled breath ammonia (NH_3_) for a healthy human being is approximately below 0.96 ppm, which is a bio-marker for kidney malfunction or renal disease. In contrast, patients with end-stage renal disease exhibit concentrations ranging from about 4.88 ppm [1]. Currently, flexible chemiresistive gas sensors featuring excellent portability and non-invasive have the potential to realize real-time monitoring of exhaled breath [2, 3]. Nevertheless, the performance of these gas sensors has been hindered by challenges related to high sensitivity, selectivity, and stability [4, 5].

Recently, inspired by the size effects and excellent catalytic activity of metal nanoparticles, single-atom catalysts (SACs), with isolated metal atoms dispersed on the supports, have emerged as promising gas sensing materials with excellent sensitivity, selectivity, and stability [6]. Notably, the engineered electronic structures of the metal active sites in SACs can regulate the charge distribution of metal centers, optimizing the adsorption/desorption of target gases on the catalyst surface [7]. Furthermore, the targeted adsorption properties of SACs can be improved by selecting specific metal atoms and adjusting the spatial structure around isolated single atoms.

In particular, nickel (Ni) metal atoms have attracted significant attention since the early work by Peyghan et al., which demonstrated their strong interactions with NH_3_ molecules [8]. Moreover, Yang et al. substantiated that the Ni single-atom active sites based on N, C coordination (Ni–N–C), characterized by unfilled *d* electrons and unsaturated coordination states, exhibit an electronic structure akin to noble metals and promote the hydrogen evolution reaction with high efficiency [9]. Additionally, Zhou et al. presented evidence that Ni–N–C can reduce the Gibbs free energy in the reaction [10], thereby facilitating oxygen species transfer through the spill-over effect [11]. However, the gas-sensing application of SACs has relatively been less exploited, primarily owing to the challenges posed by low specific surface area, unsuitable conductivity, and unstable charge transfer of traditional carbon-based supports in complex gas-sensing environments [12].

Titanium carbide (Ti_3_C_2_T_x_) MXene opened up new opportunities for low-concentration NH_3_ sensing, which is due to the large specific surface area and excellent charge transport ability [13]. This material has nearly free electron (NFE) states near the Fermi level, which provides stable channels for electron transport and high charge transfer ability. For instance, Khazaei et al. employing first-principles calculations, have elucidated the presence of NFE states across various MXenes [14]. These NFE states contribute to the electronic transport free of nuclear scattering, a feature markedly distinct from graphene, where NFE states typically reside well above the Fermi level [14]. Moreover, Ti_3_C_2_T_x_–MXene is easy to adsorb and anchor with other materials owing to a large number of oxygen functional groups (–O and –OH) on the surface of Ti_3_C_2_T_x_ sheets, expected to address the issues of unstable charge transfer in SACs [15]. However, the weak gas-sensing response caused by the narrow band gap of Ti_3_C_2_T_x_ significantly limits the further increase of gas-sensing performance [16]. The interfacial confinement of the Ti_3_C_2_T_x_–MXene with N, C coordinated Ni single atoms can optimize the overall band gap of the composite, promoting effective electron transfer and potentially leading to improved gas-sensing performance.

Furthermore, traditional gas sensors rely on sputtering technology to construct metal interdigital electrodes, which can lead to Schottky barriers at the metal–semiconductor interface, obstructing charge transfer. It’s urgent to develop a novel fabrication process to overcome this limitation, ensuring efficient charge transfer and higher gas sensing performance. Recent evidence suggests that employing flexible nanomaterial electrodes with lower work functions by printing technologies, such as MXene-based non-metallic electrodes, can enhance sensing performance by forming ohmic contacts at the metal–semiconductor interface [17]. In particular, inkjet printing allows precise deposition of nanomaterials in a non–contact, additive patterning, and mask-less manner on various substrates, facilitating the manufacturing of large-scale, cost-effective electronic devices.

It’s worth noting that Ti_3_C_2_T_x_–MXene-based electrodes are susceptible to oxidative degradation when exposed to water and/or oxygen owing to surface terminations and defects. This leads to structural transformations that can affect its stability [18]. There are some efforts to minimize Ti_3_C_2_T_x_–MXene interaction with moisture and oxygen. For instance, Green et al. demonstrated that the L-ascorbic acid molecules could protect MXene nanoflakes by blocking surface active sites from reacting with water [19]. Stanciu et al. used fluoroalkylsilane molecules for surface treatment, creating a superhydrophobic protection layer on Ti_3_C_2_T_x_, which showed improved hydration stability in humid environments [20]. However, these surface modifications may affect the electrical conductivity of Ti_3_C_2_T_x_–MXene, potentially impacting the performance of gas sensors [21]. To address this issue, poly(3,4-ethylenedioxythiophene):poly(styrenesulfonic acid) (PEDOT:PSS), as one of the conductive polymers, is anticipated to enhance the electrical conductivity of Ti_3_C_2_T_x_–MXene electrodes while shielding the Ti_3_C_2_T_x_–MXene nanosheet from oxidative degradation [22]. This end-sealing passivation strategy on the defect sites with a conjugated hydrogen bond network is expected to improve both the stability and conductivity for MXene-based gas sensors.

Herein, a paper-based fully flexible sensor was prepared to detect low concentrations of NH_3_ with high sensitivity, selectivity, and stability at room temperature. Ti_3_C_2_T_x_–MXene interfacially confined with N, C coordination Ni single atoms (Ni–N–C/Ti_3_C_2_T_x_), was prepared using an electrostatically adsorbed strategy, leading to a high response towards NH_3_. The mechanism behind its high gas sensing performance was systematically investigated, mainly attributed to the catalytic activation and targeted adsorption properties of the N, C coordinated Ni single-atom active sites with a similar electronic structure to noble metal. Additionally, a dual-channel sensing mechanism of both chemical and electronic sensitization of Ni–N–C/Ti_3_C_2_T_x_ promotes effective electron transfer to the two-dimensional (2D) MXene conductive network, thereby amplifying the sensing signal of NH_3_ gas molecules. Furthermore, the long-term stability was significantly improved by the conjugated hydrogen bond network end-sealing passivation effect on Ti_3_C_2_T_x_–MXene electrode edge defects, which was induced by the organic solvent N-methylpyrrolidone (NMP) and PEDOT:PSS. Overall, this work provides important insights into developing high-performance flexible gas sensors and expands the potential applications of MXene and SACs.

## Experimental Section

### Materials and Apparatus

The synthesis method for nitrogen-doped porous carbon (N–C), the distribution of Ni nanoparticles in nitrogen-doped porous carbon (Ni NPs/N–C), Ni–N–C, and Ni–N–C/Ti_3_C_2_T_x_, as well as the fabrication process for MXene-based electrodes, fully flexible gas sensors, and the apparatus involved in this work, are all detailed in the Supporting Information.

### Gas Sensor Measurements

The gas sensors were tested in a self-made gas sensing test system in Fig. S1. Before introducing the target gas, the chamber was purified using dry compressed air to establish and stabilize the baseline signal. The different concentrations of target gas were diluted with compressed air as the carrier gas which was controlled by two mass flow controllers (MFCs) and injected into the gas mixing chamber. The flow direction of the diluted target gas and background gas was controlled using a four-way valve. To prevent any fluctuations when switching the gas path, the flow rates of both the diluted target gas and the background gas were maintained at the same level. To understand the real-time sensor response and change of current using the Agilent 4156C semiconductor parameter analyzer (Agilent Technologies, USA) at room temperature (25 °C), a constant working voltage of 500 mV was applied between the sensor electrodes. By changing the type of target gas (NH_3_, H_2_, NO_2_, NO, and CO_2_), we conducted selective analysis of the sensors. It should be noted that, unlike these gases, the saturated vapors of acetone and ethanol were obtained through the bubbling method. Specifically, dry compressed air was divided into two gas paths, one of which passed through acetone or ethanol liquid to produce saturated vapors of these substances. Under the different levels of humidity, we studied the influence of humidity on NH_3_ sensing, and the commercial hygrometer was employed to measure the relative humidity of the test chamber. The sensor response (*S*) was defined as *S* =|*R*_a_–*R*_g_|*/R*_a_, where *R*_a_ corresponded to the resistance of the sensor in dry air and *R*_g_ corresponded to the resistance of the sensor in the target gas. The response time (*τ*_*res.*_) and recovery time (*τ*_*rec.*_) were defined by reaching 90% of the saturation of the response and recovery curve.

### Computational Details

The geometry optimization and electronic structure were studied through the first-principles density functional theory (DFT) calculations in the Vienna Ab-initio Simulation Package (VASP) with a projector augmented wave (PAW) method [23, 24]. The exchange–correlation function was described by the generalized gradient approximation (GGA) and the Perdew-Burke-Ernzerhof (PBE). Furthermore, the DFT-D3 correction method was used to analyze the vdW interaction. The electron wave function was expanded projector augmented waves, with the cutoff energy of 450 eV. In addition, the Brillouin-zone sampling was employed using a 3 × 3 × 1 Gamma-centered k-mesh. All atomic coordinates underwent relaxation until both the energy and the Hellmann–Feynman force reached the convergence criteria of 1 × 10^−5^ eV and 0.02 eVÅ^−1^, respectively. The charges transferred between distinct gas sensing materials and gas molecules were evaluated using Bader analysis [25].

## Results and Discussion

### Synthesis and Characterization of Ni–N–C/Ti_3_C_2_T_x_

**Fig. 1:**
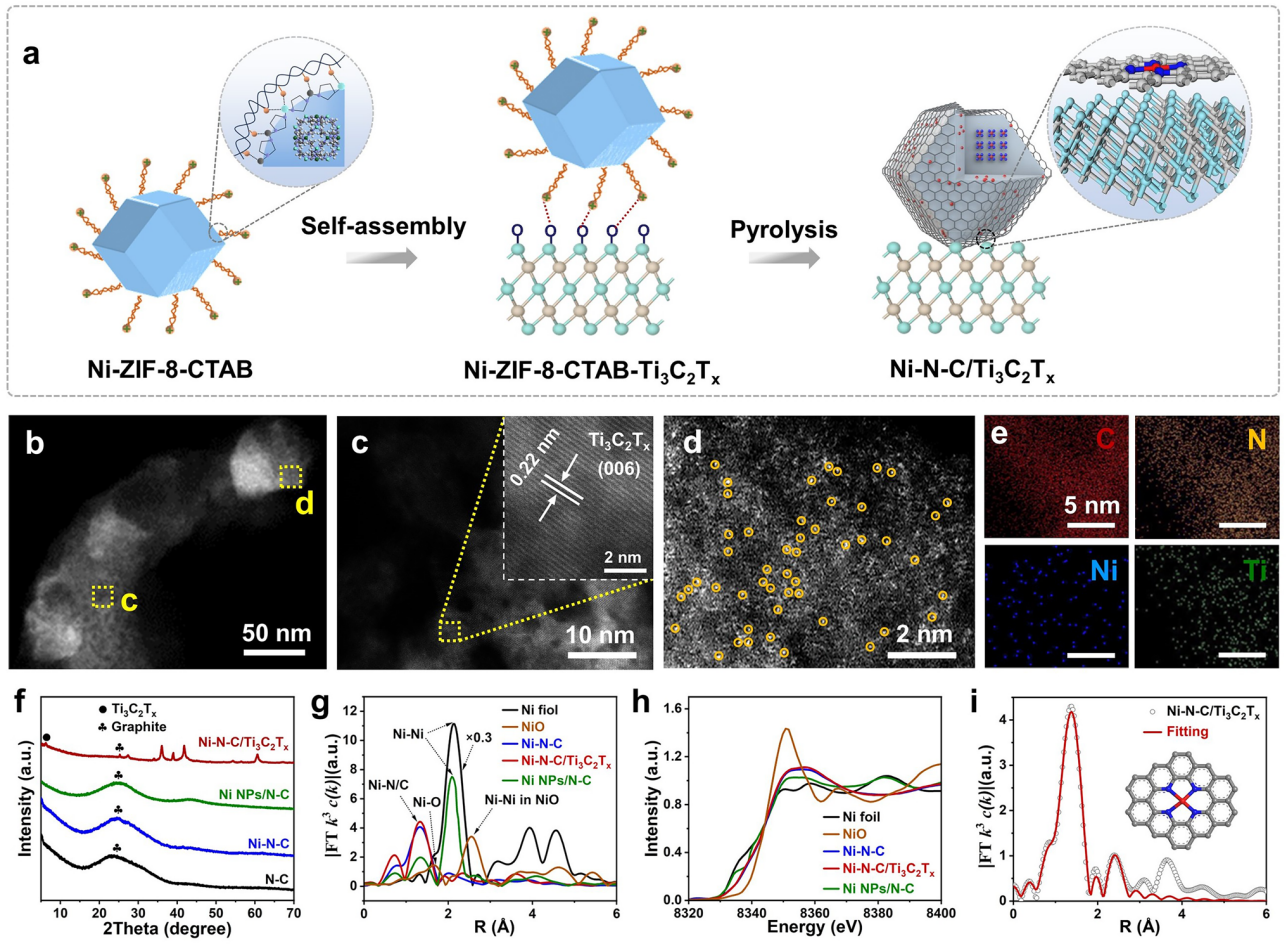
The preparation process and structure characterizations of Ni–N–C/Ti_3_C_2_T_x_. **a** Preparation process of Ni–N–C/Ti_3_C_2_T_x_. **b–d** High-angle annular dark-field scanning transmission electron microscopy (HAADF-STEM) images of Ni–N–C/Ti_3_C_2_T_x_ compound. **e** The corresponding energy-dispersive X-ray spectroscopy (EDS) for C, N, Ni, and titanium (Ti) elements of Ni–N–C/Ti_3_C_2_T_x_ compound. **f** X-ray diffraction (XRD) of N–C, Ni–N–C, Ni NPs/N–C, and Ni–N–C/Ti_3_C_2_T_x_ compounds. **g** Fourier transform (FT) *k*^3^-weighted *c*(*k*) function of the extended X-ray absorption fine structure (EXAFS) spectra. **h** Ni K-edge X-ray absorption near-edge structure (XANES) spectra. **i** Corresponding EXAFS fitting curves for Ni–N–C/Ti_3_C_2_T_x_. Inset is the proposed NiN_4_ architecture

A scheme illustrating the Ni–N–C/Ti_3_C_2_T_x_ compound formation process is summarized in Fig. 1a. The organometallic reaction of zinc ions (Zn^2+^), Ni^2+^, 2-methylimidazole (MeIM) ligands, and cetyl trimethyl ammonium bromide (CTAB) capping agent initially drove the formation of Ni-Zeolitic imidazolate framework-8 (ZIF-8)-CTAB precursor. The high crystallinity and ultrafine porosity of Ni–ZIF–8 polyhedrons provide abundant Zn^2+^ and Ni^2+^ sites that easily coordinate with the hydrophilic groups of the CTAB surfactants. Since the Ti_3_C_2_T_x_ flakes are negatively charged and hydrophilic due to the surface groups (–O, –OH, and –COOH), the positive group of CTAB-surfactant molecules in Ni–ZIF–8–CTAB precursor is electrostatically adsorbed onto the Ti_3_C_2_T_x_ flakes, resulting in effectively capping the Ti_3_C_2_T_x_ surface.

The subsequent high-temperature pyrolysis in an inert atmosphere was performed, where the carbonization of CTAB and organic linkers as well as the evaporation of Zn species occurred, resulting in N, C coordination Ni single atoms interfacially confined on the surface of MXene nanosheets. In particular, the cohesive interactions between the CTAB surfactant and the Ni–ZIF–8 nanocrystals create a unique confinement effect and prevent the collapse of the internal microporous carbonized structures derived from Ni–ZIF–8 polyhedrons, as well as reducing the agglomeration of neighboring Ni single atomic sites. Furthermore, the CTAB surfactant capped on the Ti_3_C_2_T_x_ surface was transformed into carbon coating, as shielding to minimize the unstable of Ti_3_C_2_T_x_ and promote the direct transmission of electrons. According to previous literature, high-temperature calcination of Ti_3_C_2_T_x_–MXene in an inert atmosphere is anticipated to reduce the presence of –OH, –O, and other functional groups on its surface, thereby enhancing gas-sensing stability [18]. Simultaneously, this process is expected to increase the interlayer spacing of Ti_3_C_2_T_x_–MXene, which should improve gas adsorption and desorption ability [18]. Importantly, Ti_3_C_2_T_x_–MXene maintains its hexagonal crystal structure even after undergoing high-temperature calcination [26]. Furthermore, thermogravimetric analysis (TGA) was performed to track the pyrolysis of Ni–ZIF–8 and Ni–ZIF–8–CTAB–Ti_3_C_2_T_x_ (Fig. S2). The actual loading of Ni in Ni–N–C and Ni–N–C/Ti_3_C_2_T_x_ was 0.10% and 0.25% (Table S1), respectively, as measured by inductively coupled plasma optical emission spectroscopy (ICP-OES).

Transmission electron microscopy (TEM), high-angle annular bright-field scanning (HAABF-STEM), and HAADF-STEM images were carried out to reveal the specific structures and morphologies of the samples (Figs. 1 and S3–S6). Notably, the Ni–ZIF–8-CTAB–Ti_3_C_2_T_x_ exhibited distinctive core–shell morphology in Fig. S6a, b, with the core derived from the Ni–ZIF–8 nanocrystal, and the shell derived from the CTAB surfactant layers. The high-temperature pyrolysis process generated mesopores at the edge of the polyhedron due to the partial decomposition of the CTAB, which increased the availability of active sites and improved gas diffusion (Figs. 1b and S6c, d). Furthermore, the cohesive interactions between the Ti_3_C_2_T_x_ flakes and the Ni–ZIF–8 nanocrystals give rise to a unique confinement effect at high temperatures, leading to the N, C coordinated Ni single atoms material becoming confined exclusively to the surface of the Ti_3_C_2_T_x_. The HAADF-STEM images (Fig. 1c, d) indicate that the lattice fringe spacings of 0.22 nm correspond to the (006) plane of Ti_3_C_2_T_x_ and the atomical N, C, and Ni species interfacially confined on the surface of Ti_3_C_2_T_x_. Additionally, combined with EDS analysis (Fig. 1e), the uniformly distributed Ni, N, C, and Ti signals were detected, which confirmed that the neighboring Ni^2+^ existed in a monodisperse state.

As for the Ni–ZIF–8–CTAB–Ti_3_C_2_T_x_ hybrids, the XRD peaks located at 6.2°, 7.4°, 12.8°, and 18.2° were observed, corresponding to the (002) plane of Ti_3_C_2_T_x_ gas sensing material [27], the (011), (112), and (222) planes of ZIF-8 [28], respectively (Fig. S7a, b), which would ensure the successful transformation of hybrid precursor to Ni–N–C/Ti_3_C_2_T_x_ heterostructures after pyrolysis at 850 °C under argon gas flow (Fig. 1f). Nitrogen (N_2_) adsorption/desorption analysis indicated that the Ni–N–C/Ti_3_C_2_T_x_ composite exhibited significantly higher fractions of mesopores and a larger specific surface area compared to the Ni–N–C sample, which would facilitate gas adsorption/diffusion properties (Fig. S8 and Table S2) [29].

The chemical bonding and composition evolution of species in the Ni NPs/N–C, Ni–N–C, and Ni–N–C/Ti_3_C_2_T_x_ materials were analyzed using X-ray photoelectron spectroscopy (XPS) (Fig. S9). The N 1*s* spectra of three materials exhibited three primary components corresponding to pyridinic-N (398.4 eV), graphitic-N (400.6 eV), and oxidized graphitic-N (403.0 eV) [10]. Additionally, a peak at 399.1 eV was observed, which was attributed to pyrrole-N bonded to Ni (NiN_4_), and this was found to be the main anchoring active site for single atomic Ni due to the strong coordination affinity [10]. Moreover, the Ni–N–C/Ti_3_C_2_T_x_ compound had the largest peak area for pyrrole-N (Fig. S9h), which indicated a good agreement with the Ni doping content [10]. The binding energy of the Ni 2*p*_3/2_ peak in the three materials was higher than that of Ni^0^ (852.5 − 853.0 eV) and lower than that of Ni^2+^ (855.7 eV), revealing the valence of Ni species in these samples was usually situated between Ni (0) and Ni (II) (Fig. S10) [30]. This confirms that the *d*-orbitals of Ni hybridize with *s-* and *p*-orbitals of N, resulting in unfilled *d* electrons, which exhibited similar characteristics with noble metals [9].

Further analysis using XANES and EXAFS spectroscopy revealed the electronic structure and coordination environment of Ni–N–C/Ti_3_C_2_T_x_. Interestingly, the Fourier transform *k*^3^-weighted *c*(*k*) function of the EXAFS spectra for Ni–N–C and Ni–N–C/Ti_3_C_2_T_x_ composites showed a main peak located at 1.31 Å, which was attributed to Ni–N/C coordination (Fig. 1g), confirming Ni single-atom active sites coordinate with N and C. Conversely, the major peak for Ni NPs/N–C was located at 2.15 Å, belonging to the Ni–Ni coordination [30]. As demonstrated in the XANES curves of the Ni K-edge for the three samples, the position of the absorption edge was located between that of Ni foil and NiO (Fig. 1h), which indicated that the valence state of Ni in these samples was in agreement with the XPS results. The first shell coordination number of Ni in Ni–N–C/Ti_3_C_2_T_x_ was approximately 3.8, with an average bond length of Ni–N of 1.85 Å (Table S3). Least-squares EXAFS fitting results revealed that the local atomic structure around Ni in Ni–N–C/Ti_3_C_2_T_x_ involved coordination by four N, forming the NiN_4_ structure interfacially confined on the Ti_3_C_2_T_x_ supports, which further confirms the coordination environment of Ni active sites (Fig. 1i). The coordination number of Ni in Ni–N–C was about 3.4, implying that the structure of N, C coordinated Ni single atoms consisted of two components, NiN_3_ and NiN_4_ (Fig. S11 and Table S3) [31].

### Synthesis and Characterization of MXene-Based Electrodes

**Fig. 2:**
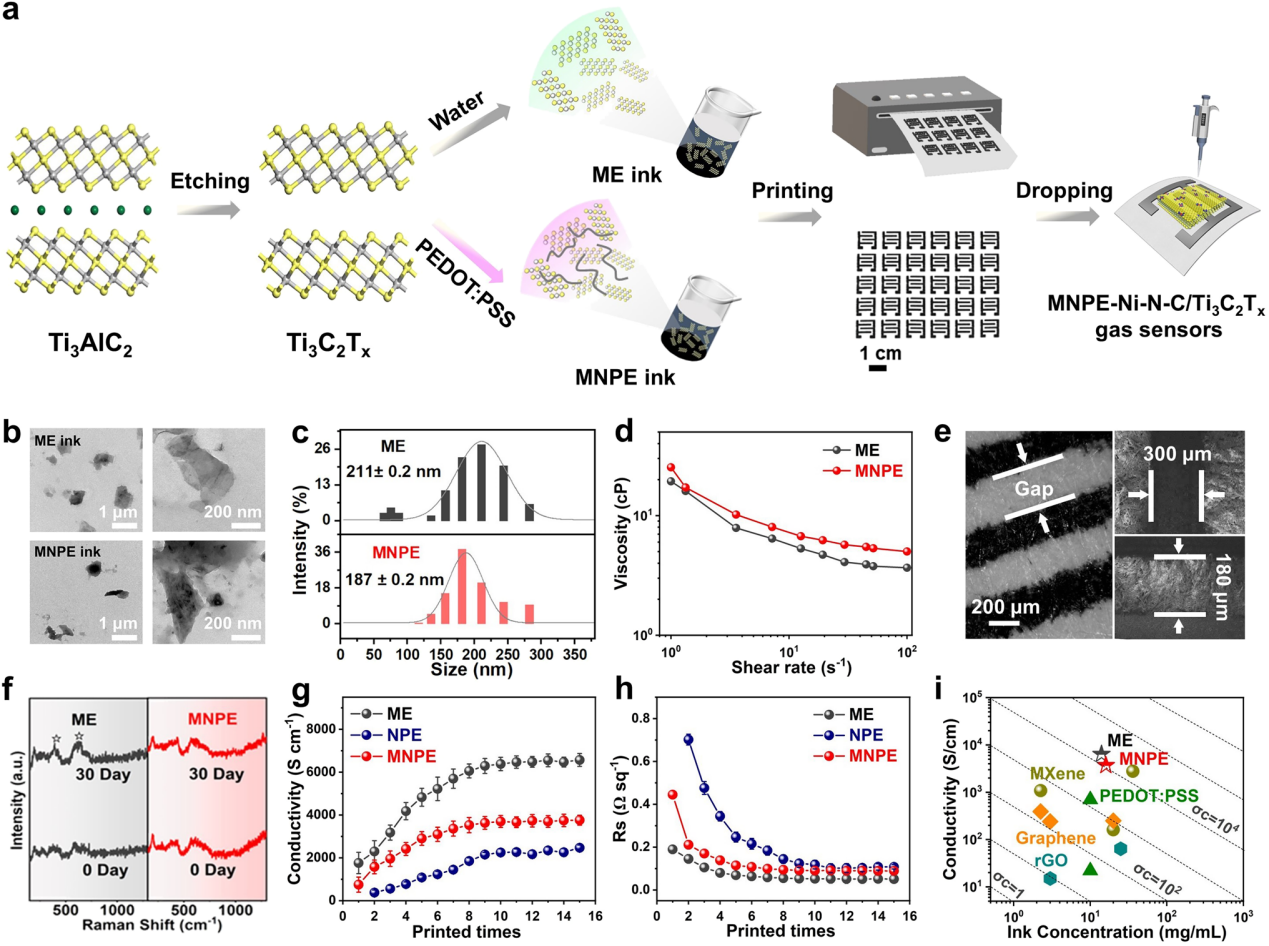
Schematic illustration of MXene-based inkjet-printing and characterizations of MXene-based ink. **a** Schematic illustration of the alternate inkjet-printing of ME ink and MNPE ink set-up used in this work to produce MNPE and MNPE-based gas sensors. **b** TEM images of ME ink and MNPE ink. **c** Corresponding lateral flake size histograms of ME ink and MNPE ink. **d** Viscosity as a function of shear rate. **e** Optical microscope and scanning electron microscopy (SEM) images of MNPE on paper with different magnifications. **f** Raman spectra of ME and MNPE fresh as well as aged for 30 days at room temperature. **g** Electrical conductivity of ME, MNPE, and NPE films as a function number of printing times. **h** Sheet resistance changes of three films as a function of printed times. **i** Comparison of ink conductivity and concentration of the prepared MXene-based ink with other reported printable ink systems

Furthermore, the inkjet-printing process for fabricating gas sensor electrodes involves the use of two types of inks: aqueous Ti_3_C_2_T_x_–MXene electrode (ME) ink and hybrid Ti_3_C_2_T_x_–MXene/NMP/PEDOT:PSS electrode (MNPE) ink (Fig. 2a). The primary objective of this approach is to improve the device stability by employing the conjugated hydrogen bond network end-sealing effect of conductive polymer to passivate the MXene edge defects. The process started with the production of highly delaminated Ti_3_C_2_T_x_ precipitates through a lithium fluoride-hydrochloric acid (LiF–HCl) mixture (Fig. S12a), followed by dispersion in different solvents (deionized (DI) water or NMP/PEDOT:PSS) through an improved ultrasonic hierarchical route to prepare two inks (ME ink or MNPE ink). The ME and NPE were directly printed onto paper by continuous inkjet printing using the aqueous ME and MNPE inks, respectively. In addition, the ME ink consisted of two primary constituents: delaminated Ti_3_C_2_T_x_ nanosheets which served as the active material, and DI water which acted as the solvent. However, this composition renders the ME vulnerable to oxidative degradation reactions when exposed to water and/or oxygen, and the absence of secondary solvents reduces the stability of the ME ink. Moreover, the MNPE was prepared by alternating inkjet printing of the ME and MNPE inks onto paper 10 times to achieve the desired conductivity levels and improve the stability of the device electrodes. Subsequently, ethanol dispersions containing various gas-sensing materials were applied onto the ME and MNPE to construct a range of gas sensors.

This simple ME ink (14 mg mL^−1^) formulation had a size distribution of the Ti_3_C_2_T_x_ flakes around 211 nm (Fig. 2b, c), owing to the hydrophilic nature and negative surface charge of the Ti_3_C_2_T_x_ nanosheets at neutral pH [32]. Furthermore, the MNPE hybrid ink (16 mg mL^−1^, 187 nm) had a smaller Ti_3_C_2_T_x_ nanosheet size than the ME ink, avoiding nozzle blockage, as the NMP/PEDOT:PSS mixed solvent in the ink prevented reaccumulation of Ti_3_C_2_T_x_ layers. Both ME and MNPE inks displayed non-Newtonian and shear-thinning (pseudoplastic) behavior during viscosity-shear rate testing, which facilitated the successive jetting and fast curing of the inks during printing (Fig. 2d) [33].

In contrast to ME ink (− 32.5 mV), the presence of sulfonic acid groups in PEDOT:PSS in the MNPE ink increased electrostatic repulsion between adjacent Ti_3_C_2_T_x_ nanosheets, resulting in a lower zeta potential of − 39.1 mV (Fig. S12b). Both ME and MNPE inks exhibited electrostatically stable suspensions with zeta potential below − 30 mV, which were essential for inkjet printing [34]. The *Z* values of ME and MNPE inks (Table S4), used to describe printability, were 10.44 and 4.18, respectively. Generally, the *Z* value of inks suitable for inkjet printing is regarded as between 1 and 14, indicating that both inks can be sprayed stably during printing [35].

Appropriate substrate wettability and ink drying were crucial for the formation of uniform films [36]. Notably, MNPE ink has a lower surface tension of 15.3 mN m^−1^ due to the addition of NMP/PEDOT:PSS solvent (Fig. S12c), which interfered with weak marangoni flow and improved print resolution [33]. Consequently, the inkjet-printed lines using MNPE ink have a width of 180 µm and a gap of 300 µm (Fig. 2e). In contrast, the lines printed produced with ME ink have a width of 300 µm and a gap of 500 µm (Fig. S12d). Furthermore, the optical images of Ti_3_C_2_T_x_-based electrode inks and films exhibited excellent printing adaptability (Figs. S13–S18). Furthermore, the digital multimeter was utilized to record the resistance of the ME and MNPE during the mechanical deformations in real-time in Fig. S19, which proved that the MNPE had excellent bending resistance stronger than that of ME, as the effect of NMP in the ink changed the conformation of the PEDOT chains, then the positively charged PEDOT chains and the negatively charged PSS chain, interacted with MXene nanosheets, forming a conjugated hydrogen bond network and creating conductive tunnels that facilitated charge transfer [37].

Moreover, after aging the ME for 30 days, peaks at 393 and 620 cm^−1^ assigned to B_1g(1)_ and E_g(3)_ vibrational modes of anatase titanium dioxide (TiO_2_) were detected in its Raman spectrum (Fig. 2f). In contrast, MNPE had well preserved and there were no characteristic peaks corresponding to anatase TiO_2_ in the Raman spectrum of MNPE even after 30 days of storage because the interaction between Ti_3_C_2_T_x_–MXene nanosheets with the rich surface groups and PEDOT:PSS, helped to form hydrogen bond network that protected the edge of the Ti_3_C_2_T_x_–MXene nanosheets and improved the environmental stability of the MNPE [38].

Notably, increasing the number of printing cycles led to higher conductivity (up to 6380 and 3700 S cm^−1^ in Fig. 2g, h) and lower square resistance for both the ME and MNPE, respectively. However, when printing the MNPE ink at room temperature to form an NPE, the conductivity of the NPE (2250 S cm^−1^) is relatively lower compared to the ME and MNPE. The conductivity and square resistance of the two electrodes stabilized after 10 cycles, making 10 cycles the preferred number of printing cycles for the manufacture of gas sensor electrodes. The ME and MNPE at room temperature were found to have the same order of magnitude conductivity as metals such as Zn and silver (Ag), and higher metallic conductivity compared to previous Ti_3_C_2_T_x_-based electrodes (Table S5). The ME and MNPE possess distinct advantages over other materials, particularly in eliminating the requirement for ultraviolet (UV) curing or annealing processes. This feature makes them highly suitable for low-temperature printed electronics on surfaces like paper and flexible plastic bases. To evaluate the electronic network properties of printable ink, the key figure of merit (FoM = σc) was used, where a higher FoM means higher conductivity and faster printing speed when the thickness of the film is constant [39]. The ME and MNPE inks reached higher FoM values of 89,320 and 59,200 S cm^−1^ mg mL^−1^, respectively (Fig. 2i), compared to other reported printable inks (Table S6).

### NH_3_ Detection Based on the Ni–N–C/Ti_3_C_2_T_x_ Gas Sensors

**Fig. 3:**
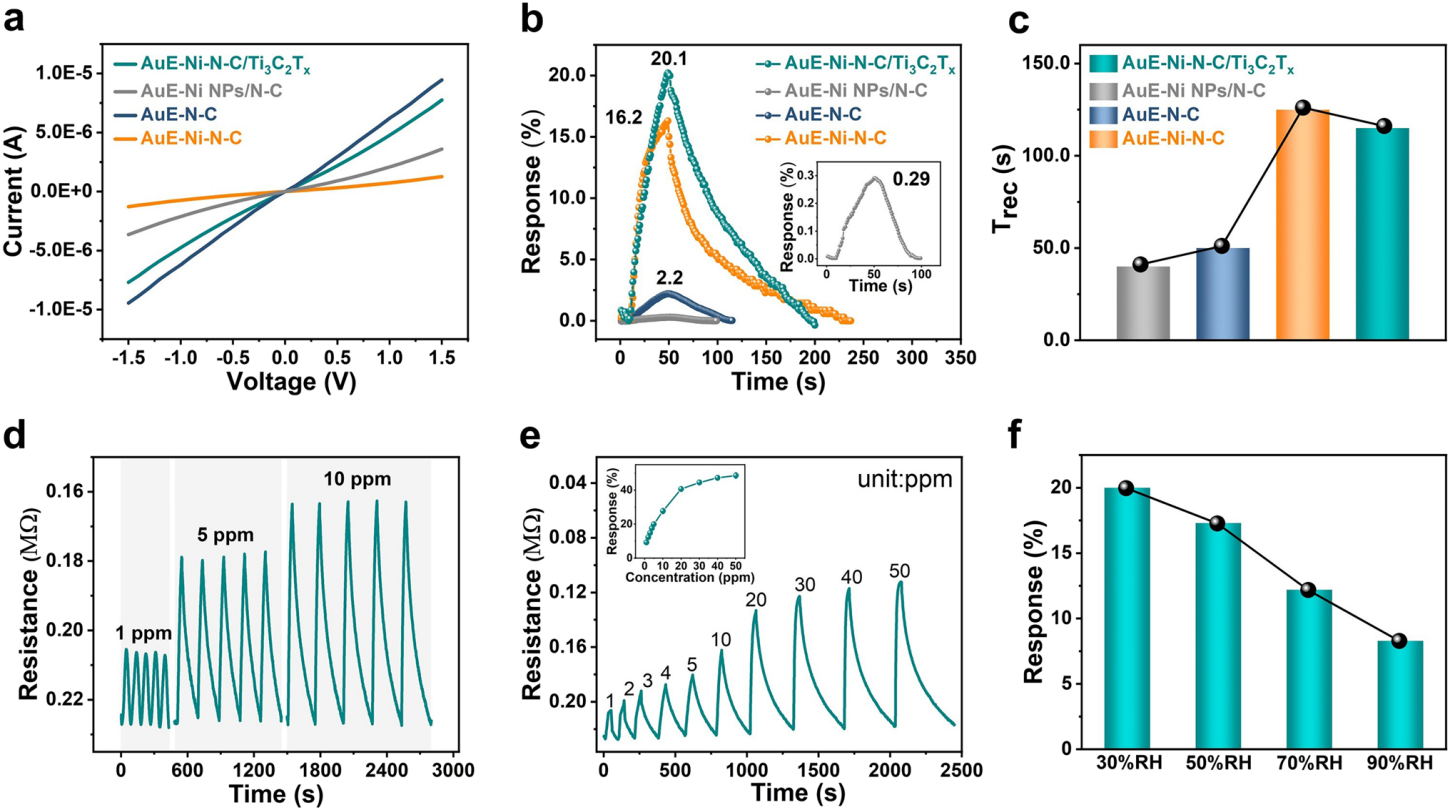
Performance of AuE–Ni–N–C/Ti_3_C_2_T_x_ gas sensor for low-concentration NH_3_ detection. **a** *I-V* curves of AuE–N–C, AuE–Ni–N–C, AuE–Ni NPs/N–C, and AuE–Ni–N–C/Ti_3_C_2_T_x_ sensors. **b** Sensing transients of the four sensors mentioned above exposed to 5 ppm NH_3_ at room temperature. **c** Comparison of the recovery time of the four sensors mentioned above to 5 ppm NH_3_ at room temperature. **d** Repeatability of AuE–Ni–N–C/Ti_3_C_2_T_x_ sensor toward 1, 5, and 10 ppm NH_3_. **e** Experimental real-time gas response curve of AuE–Ni–N–C/Ti_3_C_2_T_x_ sensor at different NH_3_ concentrations. The inset is the responses as a function of the gas concentration to different NH_3_ concentrations from 1 to 50 ppm at room temperature for the AuE–Ni–N–C/Ti_3_C_2_T_x_ sensor. **f** Comparison of the response of the AuE–Ni–N–C/Ti_3_C_2_T_x_ sensor to 5 ppm NH_3_ under different relative humidity (RH) at room temperature

The Ni–N–C/Ti_3_C_2_T_x_ gas sensor has the potential to detect the NH_3_ molecules in exhaled breath. Having prepared and characterized samples, we then set out to evaluate their gas sensing performances towards NH_3_ in a laboratory self-made gas sensing test system (Fig. S1). The gas sensors were integrated by the Aurum electrode (AuE) and the gas-sensing films (N–C, Ni NPs/N–C, Ni–N–C, and Ni–N–C/Ti_3_C_2_T_x_). The contact mode between AuE and N–C film was demonstrated to be ohmic-type for the linear current–voltage (*I-V*) curves (Fig. 3a). However, the AuE–N–C sensor exhibited a low response to 5 ppm NH_3_ (2.2% in Figs. 3b and S20). Compared to the AuE–Ni NPs/N–C and AuE–Ni–N–C gas sensors, the AuE–Ni–N–C/Ti_3_C_2_T_x_ sensor exhibited the best response to 5 ppm NH_3_ (20.1% in Fig. 3b) and the shortest recovery time (115 s in Fig. 3c). This enhancement can be attributed to the catalytic activation achieved by Ni–N–C/Ti_3_C_2_T_x_. Firstly, the catalytic effect can induce a low energy barrier for the sensing elemental reaction, while the electronic structure of Ni–N–C increases the number of chemisorbed oxygen species at the gas–solid interface, thereby promoting the sensitivity of the sensor to NH_3_ [10].

Furthermore, the interfacial confinement of Ni–N–C and Ti_3_C_2_T_x_–MXene also contributes a dual-channel sensing mechanism of both chemical and electronic sensitization, which facilitates efficient electron transfer to the 2D MXene conductive network, resulting in enhancing the NH_3_ gas molecule sensing signal. To understand the dual-channel sensing mechanism, the gas adsorption–desorption models in addition to the surface reactions on the surface of the Ni–N–C/Ti_3_C_2_T_x_ should be considered, thus pure N_2_ was used as the carrier gas for NH_3_ to remove the effect of oxygen in Fig. S21. The response of the Ni–N–C/Ti_3_C_2_T_x_ sensor to 5 ppm NH_3_ in an N_2_ atmosphere was 8.4% in Fig. S21, which was lower than in air atmosphere (20.1%), suggesting that while the redox response dominates the sensing mechanism, the adsorption–desorption process also plays a role in the gas performance. The gas sensing mechanism in the N_2_ atmosphere is different from that in air conditions, for which the charge can directly transfer between NH_3_ and the surface of gas sensing material with the changes of resistance.

The repeatability of the AuE–Ni–N–C/Ti_3_C_2_T_x_ gas sensor in detecting 1, 5, and 10 ppm of NH_3_ over five cycles, respectively, was excellent (Fig. 3d). Under cyclic exposure to NH_3_ ranging from 1 to 50 ppm, the time-related dynamic response of the AuE–Ni–N–C/Ti_3_C_2_T_x_ gas sensor exhibited a stable response and recovery features in Fig. 3e. With the calculated limit of detection (LOD) = 3 × S_*Standard error*_/K_*Slope*_, the AuE–Ni–N–C/Ti_3_C_2_T_x_ gas sensor was predicted to have a lower detection limit of 29.3 ppb (Fig. S22b), compared to the AuE–N–C and AuE–Ni–N–C devices (824.0 and 50.9 ppb, respectively, in Fig. S23).

Moreover, RH was found to have a significant impact on the gas sensing performance of the AuE–Ni–N–C/Ti_3_C_2_T_x_ sensor at room temperature. With increasing humidity, the baseline resistance of the sensor gradually decreases. This is due to the reaction between surface oxygen species and H_2_O molecules, producing OH^−^ and H^+^ ions. This reaction releases more electrons back to the conduction band of the sensing material, as described by Eq. (1), contributing to the reduction in baseline resistance (Fig. S24a). When NH_3_ is introduced, it donates electrons to the sensitive material, causing a further decline in resistance (Fig. S24b). However, it is notable that as the RH increases, the response of the AuE–Ni–N–C/Ti_3_C_2_T_x_ sensor decreases (Fig. 3f). This can be attributed to the competition between H_2_O molecules and O_2_ molecules for capturing electrons from the Ni–N–C/Ti_3_C_2_T_x_ films, as described by Eq. (1), which interferes with the reaction between surface oxygen species and the target gas [40]. Additionally, the AuE–Ni–N–C/Ti_3_C_2_T_x_ sensor exhibited noticeable baseline drift and incomplete recovery behavior under 5 ppm NH_3_ with 90% RH (Fig. S24b). This is primarily because the gas desorption energy is difficult to achieve at room temperature, making it challenging for H_2_O molecules to desorb from the surface of the gas-sensing material. Consequently, this leads to imperfect recovery behavior and baseline drifts.

1 $$2\text{H}_{2} \text{O} \, + \, \text{O}_{2}^{ - } \to \, 4\text{H}^{ + } + \, 4\text{OH}^{ - } + \text{e}^{ - }$$

**Fig. 4:**
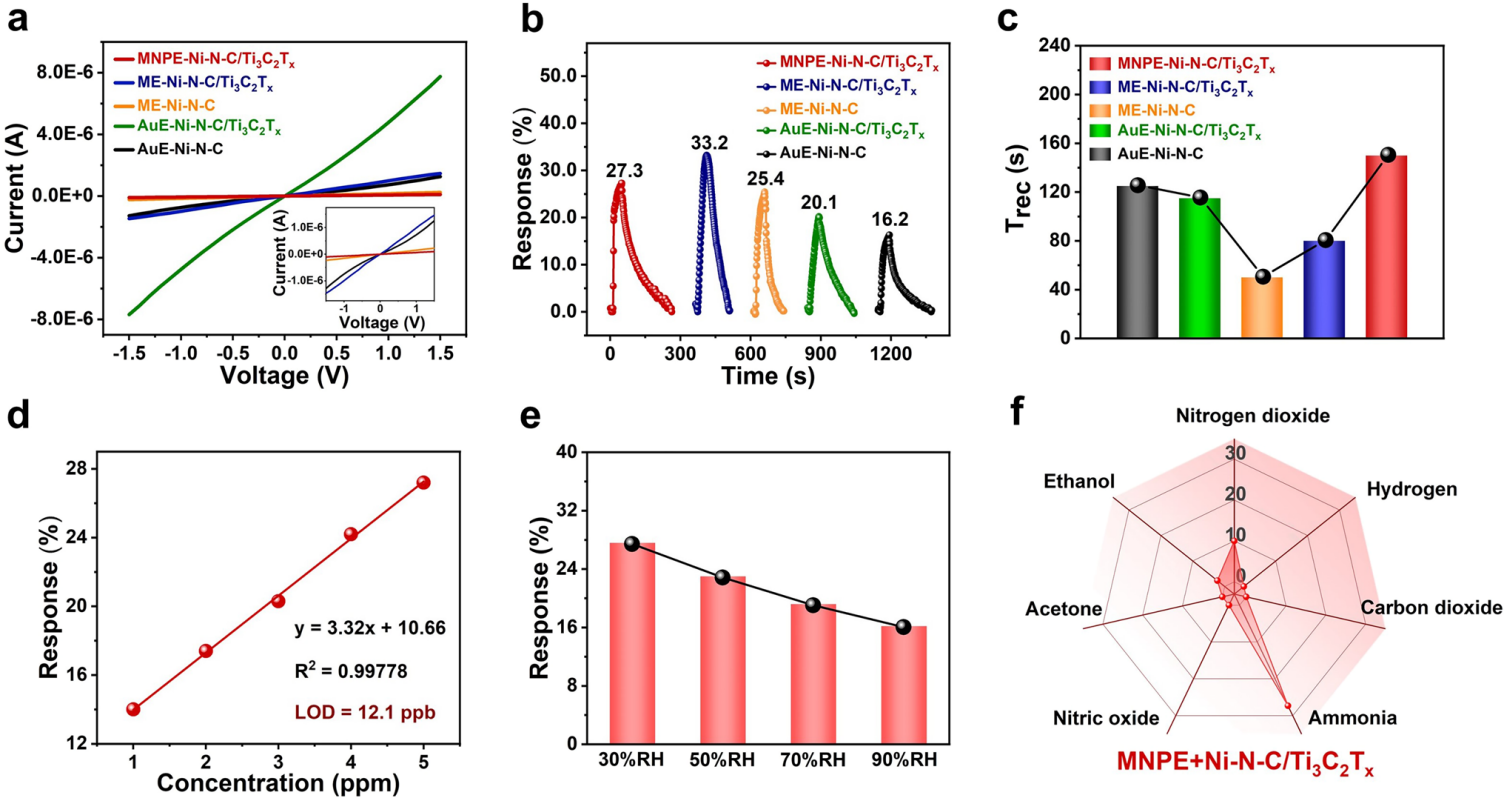
Performance of ME–Ni–N–C/Ti_3_C_2_T_x_ and MNPE–Ni–N–C/Ti_3_C_2_T_x_ gas sensors for low-concentration NH_3_ detection. **a** *I-V* curves of AuE–Ni–N–C, AuE–Ni–N–C/Ti_3_C_2_T_x_, ME–Ni–N–C, ME–Ni–N–C/Ti_3_C_2_T_x_, and MNPE–Ni–N–C/Ti_3_C_2_T_x_ sensors. The inset is the amplified *I-V* curve of AuE–Ni–N–C, ME–Ni–N–C, ME–Ni–N–C/Ti_3_C_2_T_x_, and MNPE–Ni–N–C/Ti_3_C_2_T_x_ sensors. **b** Sensing transients of the five sensors mentioned above exposed to 5 ppm NH_3_ at room temperature. **c** Comparison of the recovery time of the five sensors mentioned above to 5 ppm NH_3_ at room temperature. **d** Response curves versus NH_3_ concentration for MNPE–Ni–N–C/Ti_3_C_2_T_x_ sensor. **e** Comparison of the response of the MNPE–Ni–N–C/Ti_3_C_2_T_x_ sensor to 5 ppm NH_3_ under different RH at room temperature. **f** Selectivity of MNPE–Ni–N–C/Ti_3_C_2_T_x_ sensor to different gases (5 ppm NH_3_, 50 ppm nitrogen dioxide (NO_2_), carbon dioxide (CO_2_), nitric oxide (NO), hydrogen (H_2_), acetone-saturated steam, and ethanol-saturated steam)

To prove the unique advantages of flexible electrodes in gas sensing, the ME–Ni–N–C, ME–Ni–N–C/Ti_3_C_2_T_x_ and MNPE–Ni–N–C/Ti_3_C_2_T_x_ gas sensors were integrated by MXene-based electrodes (ME and MNPE) and the gas-sensing films (Ni–N–C and Ni–N–C/Ti_3_C_2_T_x_) for evaluating NH_3_ gas sensing performance at room temperature. The nonrectifying characteristics of the ME–Ni–N–C sensor (Fig. 4a), compared to the Schottky-type AuE–Ni–N–C sensor (Fig. 3a), demonstrated that the barrier between the ME and Ni–N–C material had a negligible effect on the Schottky barrier height (SBH). The gas sensing response of the ME–Ni–N–C/Ti_3_C_2_T_x_ sensor to 5 ppm NH_3_ (33.2% in Fig. 4b) was about 1.3 times higher than that of the ME–Ni–N–C gas sensor (25.4%). The enhanced gas-sensing performance resulted from the catalytic activation of the atomically dispersed NiN_4_ active sites and Ti_3_C_2_T_x_–MXene as a conductive network to improve the transmission efficiency of sensing charges. The ME–Ni–N–C/Ti_3_C_2_T_x_ sensor had a shorter recovery time of 5 ppm NH_3_ (80 s in Fig. 4c) compared to that of the MNPE–Ni–N–C/Ti_3_C_2_T_x_ sensor (150 s in Figs. S25 and S26), due to the excellent conductivity of ME, which has a relatively low baseline resistance and a large number of surface groups that can adsorb more target gas.

In addition, the theoretical LOD of the MNPE–Ni–N–C/Ti_3_C_2_T_x_ and ME–Ni–N–C/Ti_3_C_2_T_x_ sensors for NH_3_ was calculated to be 12.1 and 27.0 ppb, respectively (Figs. 4d and S27c), which was lower than the exhaled breath NH_3_ concentration (below 0.96 ppm) for a healthy human [41]. Furthermore, the present sensors signify a notable advancement in sensing performance when compared to previously reported MXene-based NH_3_ sensors (Table S7).

Moreover, during the humid NH_3_ sensing of the MNPE–Ni–N–C/Ti_3_C_2_T_x_ and ME–Ni–N–C/Ti_3_C_2_T_x_ sensors in Figs. 4e, S28, and S29, oxygen-enriched groups of the MNPE and ME offered more active sites for the adsorption of NH_3_ compared to the AuE–Ni–N–C/Ti_3_C_2_T_x_ sensor, thereby resulting in an enhanced response of the two sensors to NH_3_ (Table S8). Notably, the gas response of the MNPE–Ni–N–C/Ti_3_C_2_T_x_ sensor to NH_3_ changes by 40.7% under high humidity (Fig. S28a and Table S8), while the ME–Ni–N–C/Ti_3_C_2_T_x_ sensor shows a larger change of 54.2% (Fig. S29a and Table S8). Furthermore, the MNPE–Ni–N–C/Ti_3_C_2_T_x_ sensor maintains a relatively high response and stability when exposed to a high-humidity environment (90% RH) as shown in Fig. S28b, indicating its potential for use in such conditions. On the one hand, after printing MNPE on the paper surface, the end-sealing effect eliminates hydrophilic groups, thus enabling the sensor to maintain stability under high humidity. On the other hand, the high porosity of the paper substrate allows MNPE ink to cover the surface and penetrate into the cellulose fibers, forming a moisture-resistant protective barrier. In contrast, the ME–Ni–N–C/Ti_3_C_2_T_x_ sensor exhibits significant baseline drift and incomplete recovery behavior towards NH_3_ sensing under high humidity (Fig. S29c). This is mainly due to the absence of hydrogen bond interfacial interactions in the non-end-sealing aqueous Ti_3_C_2_T_x_–MXene electrode. The Ti_3_C_2_T_x_–MXene electrode in the ME–Ni–N–C/Ti_3_C_2_T_x_ sensor has numerous active functional groups, vacancies, and defects that adsorb H_2_O molecules, reducing the sensor’s ability to desorb NH_3_.

Moreover, the selectivity of AuE–Ni–N–C/Ti_3_C_2_T_x_, MNPE–Ni–N–C/Ti_3_C_2_T_x_, and ME–Ni–N–C/Ti_3_C_2_T_x_ gas sensors was tested against various gases including 5 ppm of NH_3_, 50 ppm of NO_2_, H_2_, CO_2_, NO, acetone-saturated steam and ethanol-saturated steam (Figs. 4f and S30). Compared to other gases, the three sensors showed a higher response to NH_3_, confirming that selecting specific Ni metal atoms coupled with adjustments to the spatial structure surrounding the Ni single atoms, can induce specific targeted adsorption of NH_3_, thereby significantly enhancing the selectivity of the sensors [42].

**Fig. 5:**
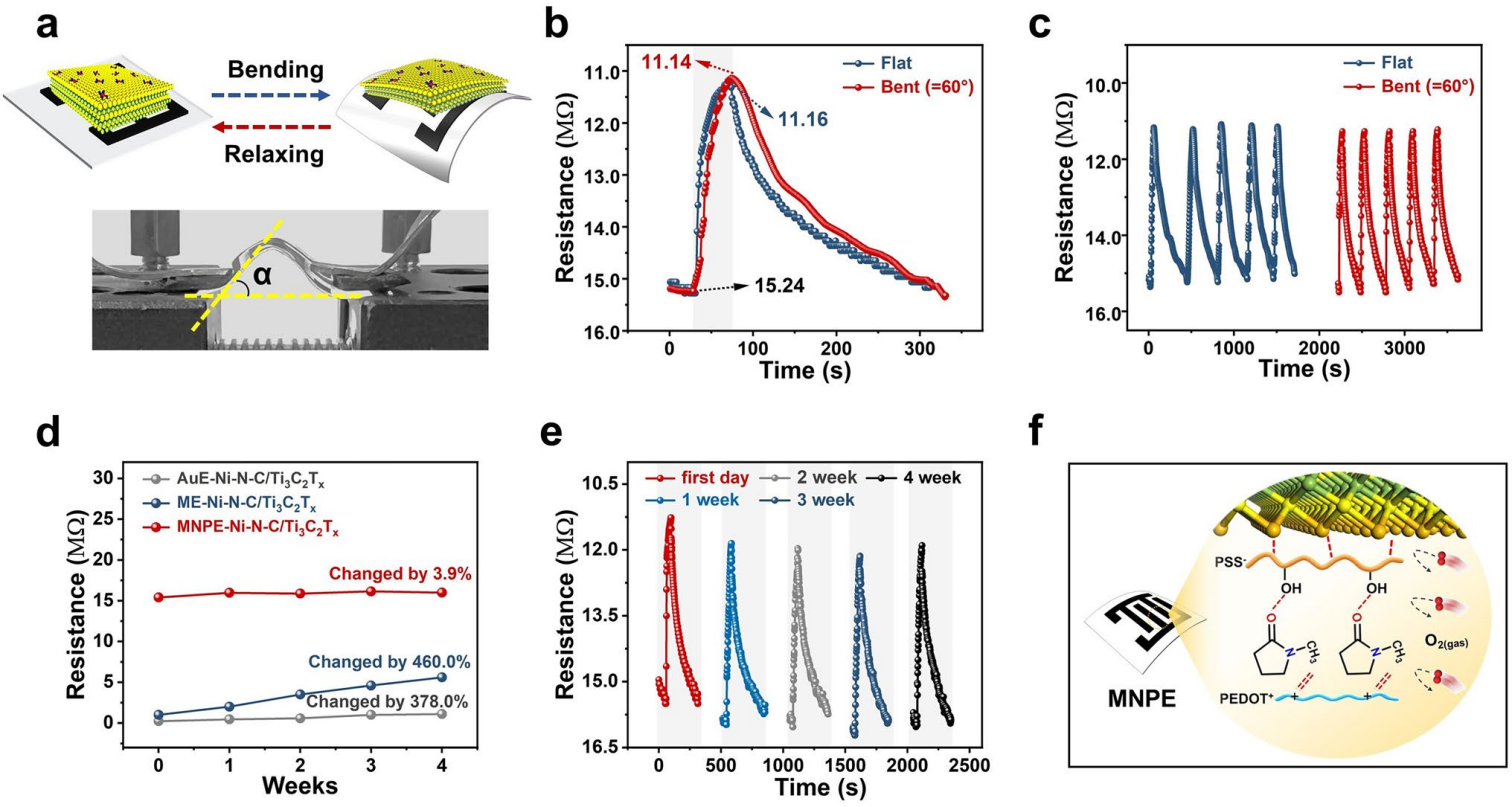
Stability analysis of MNPE–Ni–N–C/Ti_3_C_2_T_x_ gas sensor for NH_3_ detection. **a** The optical photos of the as-prepared MNPE–Ni–N–C/Ti_3_C_2_T_x_ flexible sensor after bending. **b** Resistance curves of the flexible sensor to 5 ppm NH_3_ when tested under 60° bending angles. **c** Repeatability of MNPE–Ni–N–C/Ti_3_C_2_T_x_ sensor toward 5 ppm NH_3_ before and after bending 500 times. **d** Changes in resistance of AuE–Ni–N–C/Ti_3_C_2_T_x_, ME–Ni–N–C/Ti_3_C_2_T_x_, and MNPE–Ni–N–C/Ti_3_C_2_T_x_ sensors to 5 ppm NH_3_ within 4 weeks. **e** Resistance curve of MNPE–Ni–N–C/Ti_3_C_2_T_x_ sensor to 5 ppm NH_3_ at different weeks. **f** Schematic diagram of the long-term stability mechanism for the MNPE–Ni–N–C/Ti_3_C_2_T_x_ sensor

Furthermore, the performance of flexible gas sensors can be affected by mechanical deformations, which can impact their sensitivity to gas detection (Figs. 5a–c and S31). The ME–Ni–N–C/Ti_3_C_2_T_x_ sensor demonstrated favorable mechanical performance under bending conditions, remaining stable even after five cyclic exposures to 5 ppm NH_3_ gas before and after repeated bending of 60° (500 times in Figs. 5a and S31a). However, a slight increase in baseline resistance was observed after bending (Fig. S31b), which can be attributed to the absence of interconnected conducting pathways caused by the bending condition for ME–Ni–N–C/Ti_3_C_2_T_x_ sensor. In contrast, the MNPE–Ni–N–C/Ti_3_C_2_T_x_ sensor exhibited both stable resistance and gas-sensing performance toward 5 ppm NH_3_ even after repeated bending (Figs. 5b, c, and S31d).

Furthermore, the MNPE–Ni–N–C/Ti_3_C_2_T_x_ sensor did not crack, and the sensing material exhibited positive adhesion to the electrode (Fig. S32a–c) after repeated bending, indicating a strong interaction between the gas sensing film and the MXene-based electrode. These results demonstrate the effectiveness of utilizing a conductive polymer as an end-sealing agent for MXene, which forms a robust and homogeneous conjugated hydrogen bond network formed by the neighboring MXene nanosheets and conductive PEDOT:PSS, including covalent bonds, hydrogen bonds, and physical entanglement among polymer chains and/or MXene sheets. This approach enables stronger interfacial interactions and efficient electron transfer pathways [43].

Notably, maintaining the long-term stability of the flexible gas sensor remains a significant challenge. To evaluate the gas sensing performance, we conducted measurements during exposure to 5 ppm NH_3_ gas once a week for a total of 4 weeks. The results demonstrated that the resistance and response of the AuE–Ni–N–C/Ti_3_C_2_T_x_ sensor changed significantly by 378.0% and 54.3%, respectively (Figs. 5d, S33, and S35a). Similarly, the ME–Ni–N–C/Ti_3_C_2_T_x_ sensor also had poor long-term stability. The resistance and response of the ME–Ni–N–C/Ti_3_C_2_T_x_ sensors changed significantly by 460.0% and 35.8%, respectively (Figs. 5d, S34, and S35a). In contrast, the MNPE–Ni–N–C/Ti_3_C_2_T_x_ gas sensor exhibited exceptional long-term stability, with only a minor reduction in resistance (3.9%) and response (9.0%) over the 4 weeks (Figs. 5e and S35a, b). Furthermore, the repeatability of the MNPE–Ni–N–C/Ti_3_C_2_T_x_ sensor after five consecutive cycles of exposure to 5 ppm NH_3_ gas after four weeks confirmed its recoverability (Fig. S35c).

The excellent stability of the MNPE–Ni–N–C/Ti_3_C_2_T_x_ sensor can be attributed to two factors. Firstly, the addition of NMP and PEDOT:PSS solution results in an end-sealing passivation effect through the formation of a conjugated hydrogen bond network (Fig. 5f). Specifically, the addition of NMP to the PEDOT:PSS solution induces a conformational change in the PEDOT chains, reducing Coulomb interaction between the positively charged PEDOT chains and negatively charged PSS chains [38]. This conformational change enhances the structural stability of the solution. Additionally, the MXene nanosheets possess abundant surface groups that interact with PEDOT:PSS, leading to the formation of a conjugated hydrogen bond network [44]. This end-sealing passivation effect between MXene nanosheets and PEDOT:PSS inhibits the oxidative degradation reaction of the MXene nanosheets. Secondly, the paper substrate has high porosity, allowing the electrode inks to coat the surface and permeate through the cellulose fibers of the paper, forming antioxidant barriers. When the gas-sensing material evenly permeates into the paper fibers, the end-sealing effect protects the materials from degradation reactions with water and oxygen. Thirdly, the CTAB coating and intercalation effects shielded the Ti_3_C_2_T_x_ structure and prevented the diffusion of Ti atoms from the intermediate layer of the Ti_3_C_2_T_x_ sheet to the surface, protecting Ti atoms on the surface and enhancing structural stability [45].

### Sensing Mechanism

**Fig. 6:**
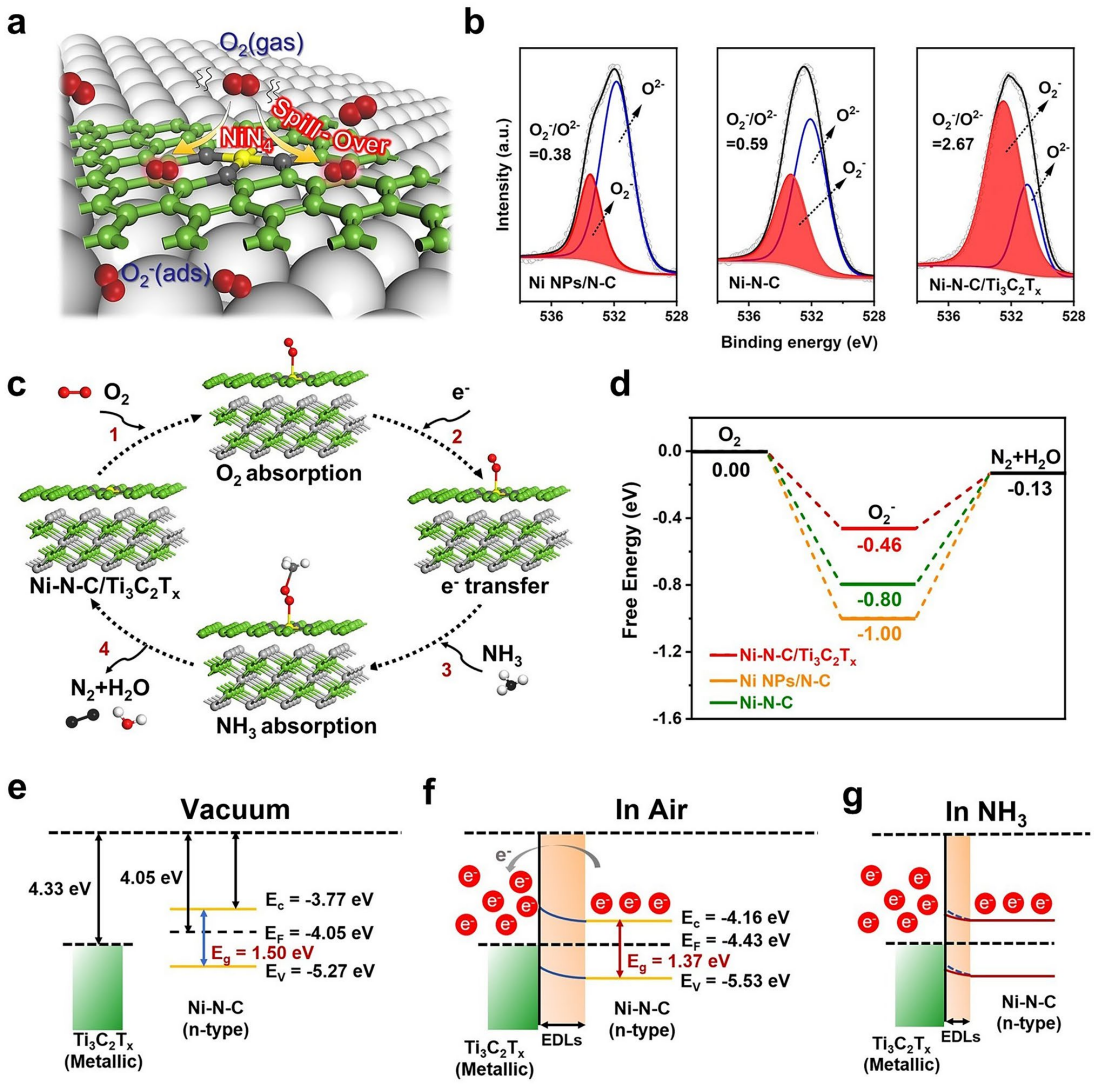
Mechanism of MNPE–Ni–N–C/Ti_3_C_2_T_x_ gas sensor for NH_3_ detection. **a** Schematic illustration of O_2_ (gas) dissociation over NiN_4_ on Ni–N–C/Ti_3_C_2_T_x_. **b** XPS spectra of Ni NPs/N–C, Ni–N–C, and Ni–N–C/Ti_3_C_2_T_x_ in the vicinity of O 1*s*. **c** Proposed structures for the redox reaction process on Ni–N–C/Ti_3_C_2_T_x_. **d** Gibbs free energy profile along the pathway from O_2_ to N_2_ and H_2_O. Schematic diagrams of work function and Fermi level position for metallic Ti_3_C_2_T_x_ and n-type Ni–N–C semiconductor **e** before contact, **f** in the air, and **g** NH_3_ at room temperature

The Ni–N–C/Ti_3_C_2_T_x_ gas-sensing material can enhance the gas-sensing performances from three aspects, that is the chemical and electronic sensitization effects as well as NH_3_ adsorption enhancement. For chemical sensitization, the electronic structure of NiN_4_ active sites in Ni–N–C facilitates the dissociation of oxygen through the spill-over effect, leading to an increase in the concentration of active O_2_^–^ species at the gas–solid interface, as seen in Eq. (2). Subsequently, these active oxygen species rapidly move towards the C and Ti_3_C_2_T_x_ supports (Fig. 6a). This has been confirmed through XPS analysis, where the O_2_^–^ to O^2–^ ratio (chemisorbed oxygen species to lattice oxygen species in Fig. 6b and Table S9) of the O 1*s* peak is higher for Ni–N–C/Ti_3_C_2_T_x_ (O_2_^–^/O^2–^ = 2.67) and Ni–N–C (O_2_^–^/O^2–^ = 0.59) compared to Ni NPs/N–C (O_2_^–^/O^2–^ = 0.38), indicating that the spill-over effect of NiN_4_ active center can increase the number of chemisorbed oxygen species on the surface of Ni–N–C/Ti_3_C_2_T_x_ and Ni–N–C [11]. Especially, the large specific surface area of Ti_3_C_2_T_x_ sheets facilitates efficient chemisorbed oxygen species transfer to the 2D MXene conductive network, resulting in increasing the number of adsorption and reaction sites for NH_3_. Moreover, when exposed to electron-donating NH_3_ gas, the chemically adsorbed oxygen on the gas-sensing materials undergoes a redox reaction with NH_3_ gas molecules, producing N_2_ and H_2_O represented by the following Eq. (3) [46].

2 $${\text{O}}_{{2}} \left( {{\text{gas}}} \right) \, + {\text{ e}}^{ - } \to {\text{ O}}_{{2}}^{ - } \left( {{\text{ads}}} \right)$$

3 $${\text{4NH}}_{{3}} \left( {{\text{gas}}} \right) \, + {\text{ 3O}}_{{2}}^{ - } \left( {{\text{ads}}} \right) \to {\text{2N}}_{{2}} + {\text{ 6H}}_{{2}} {\text{O}} + {\text{3e}}^{ - }$$

DFT calculations were performed to deepen the insight into the reaction pathways and the corresponding energy profiles of Ni NPs/N–C, Ni–N–C, and Ni–N–C/Ti_3_C_2_T_x_ in the oxidation–reduction reaction of NH_3_ (Fig. 6c, d). The reaction involves two steps, the adsorption of O_2_, and the generation of N_2_ and H_2_O in Eqs. (2 and 3). The adsorption energy of O_2_ for Ni–N–C and Ni NPs/N–C were calculated to be − 0.80 and − 1.00 eV, respectively, confirming that the O_2_ adsorption is a barrier-less step. For the redox reaction in Eq. (3) which is determined to be the rate-limiting step, Ni NPs/N–C requires a high energy barrier of 0.87 eV, while Ni–N–C only needs to overcome an energy barrier of 0.67 eV. These calculated results provide evidence that the catalytic activation effect of the NiN_4_ active sites effectively reduces the Gibbs free energy of the sensing elemental reaction, making it easier for O_2_^–^ to react with NH_3_. Furthermore, after O_2_ adsorption on Ni–N–C/Ti_3_C_2_T_x_ (− 0.46 eV), the energy barrier on the NiN_4_ active sites to yield N_2_ and H_2_O, and regenerate the active site is only 0.33 eV. Thus, compared to Ni–N–C and Ni NPs/N–C, the interfacial confinement effect of the NiN_4_ active sites and the Ti_3_C_2_T_x_ supports results in lower activation energy for the redox reaction and higher gas-sensing performances for NH_3_.

For electronic sensitization, according to our previous work [17], the Ti_3_C_2_T_x_ gas sensing materials own work function of 4.33 eV, which was higher than that of Ni–N–C (4.05 eV) confirmed by ultraviolet photoelectron spectroscopy (UPS) analysis in Fig. S36a, suggested that the Ni–N–C transferred electron density to the Ti_3_C_2_T_x_ until the Femi energy level reached equilibrium, forming a Schottky barrier in the Ni–N–C/Ti_3_C_2_T_x_ heterojunction with a work function of 4.43 eV (Figs. 6e and S36b). From the UV–visible spectroscopy (UV–vis in Fig. S37), the Ni–N–C and the Ni–N–C/Ti_3_C_2_T_x_ materials own band gap of 1.50 and 1.37 eV, respectively. When the Ni–N–C/Ti_3_C_2_T_x_ sensor was placed in ambient air, oxygen molecules catalyzed by the NiN_4_ active sites would take electrons from the conduction band of the Ni–N–C/Ti_3_C_2_T_x_ material and the electron depletion layers (EDLs) may occur in Fig. 6f, which would hinder the transfer of charge carriers and increase the baseline resistance of the sensor. However, when the nanocomposite was exposed to electron-donating NH_3_ gas, the redox reaction would release electrons back into the nanocomposite, causing a decrease in the width of the EDLs and reducing the upward band bending, ultimately leading to a decrease in the resistance of the sensor (Fig. 6g).

To understand the gas sensing mechanism, the gas adsorption–desorption models in addition to the redox reactions on the surface of the Ni–N–C/Ti_3_C_2_T_x_ should be considered, in which the charge transfer process between NH_3_ and the gas-sensing materials changes the resistance of the gas sensor. The DFT calculations in Fig. S38 provide insight into the adsorption models of NH_3_ on the N–C, Ni NPs/N–C, Ni–N–C, and Ni–N–C/Ti_3_C_2_T_x_ structures confirmed by the EXAFS results. The results indicate that NH_3_ molecules are more readily adsorbed on the surface of Ni–N–C/Ti_3_C_2_T_x_ due to the highest adsorption energy and charge transfer to NH_3_ (Table S10), reflecting the higher gas-sensing performance, which agrees with the experimental results. Moreover, Bader charge analysis indicates that the Ni and its neighboring N atoms in Ni–N–C/Ti_3_C_2_T_x_ lose 1.98 e and 2.95 e, respectively, while the C atom and Ti_3_C_2_T_x_ gain 2.76 e and 2.33 e simultaneously (Fig. S39a). More charges (5.09 e) are transferred from the NiN_4_ active centers to the supports for Ni–N–C/Ti_3_C_2_T_x_ compared with Ni–N–C (4.89 e in Fig. S39b). Therefore, the confinement effect between the NiN_4_ active sites and the supports promotes more electron transfer to the 2D MXene conductive network, resulting in the enhanced NH_3_ gas molecule sensing signal.

Furthermore, to elucidate the selectivity of the gas adsorption, the adsorption capability of various gases on Ni–N–C/Ti_3_C_2_T_x_ was also calculated (Table S11). The results reveal that NH_3_ molecules exhibit a higher propensity for adsorption on the surface of Ni–N–C/Ti_3_C_2_T_x_ compared to other gases, attributed to their highest adsorption energy and charge transfer. This observation aligns with experimental results. The pronounced adsorption capability of NH_3_ on the Ni–N–C/Ti_3_C_2_T_x_ surface validates the specific adsorption of NH_3_ by NiN_4_ active sites, thereby effectively enhancing NH_3_ detection performance.

Additionally, the response of the ME–Ni–N–C sensor to 5 ppm NH_3_ (25.4% in Fig. 4b) was higher than that of the AuE–Ni–N–C sensor (16.2%), and the recovery time (50 s) was shorter than that of AuE–Ni–N–C sensor (125 s in Fig. 4c). As a result, the use of a non-metallic electrode made of Ti_3_C_2_T_x_–MXene reduced the SBH between the homogeneous electrode and the gas sensing material, resulting in ohmic contact and improved charge transfer ability across the metal–semiconductor interface of the gas sensors (Fig. S40). Moreover, the large specific surface area of the non-metallic electrodes improved gas adsorption and reaction sites. In addition, the excellent conductivity of Ti_3_C_2_T_x_ nanosheets facilitated faster electron transport and recovery during sensing. In summary, the catalytic activation of N, C coordinated Ni single atoms efficiently reduces the Gibbs free energy of the sensing elemental reaction, while its electronic structure enhances the spill-over effect of reactive oxygen species. Furthermore, this sensor operates via a dual-channel sensing mechanism involving both chemical and electronic sensitization by the interfacial confinement of the Ti_3_C_2_T_x_–MXene with N, C coordinated Ni single atoms. And the use of a Ti_3_C_2_T_x_–MXene non-metallic electrode has been confirmed as an efficient strategy for matching the work function of gas-sensing material to enhance gas detection performances.

## Conclusions

In summary, a fully flexible MNPE combined Ni–N–C/Ti_3_C_2_T_x_ paper-based gas sensor was proposed with high sensitivity, selectivity, and stability for NH_3_ detection. Given these unique advantages, the Ni–N–C/Ti_3_C_2_T_x_ can serve as an ideal candidate for the early detection of biomarkers in exhaled breath. Compared with the traditional Au metal electrode-based gas sensors, the MNPE–Ni–N–C/Ti_3_C_2_T_x_ gas sensors exhibited a 1.7-fold higher gas sensing performance to 5 ppm NH_3_ with a response of 27.3% at room temperature and achieved a LOD of 12.1 ppb NH_3_ gas. This performance improvement can be attributed to the catalytic activation effect effectively reducing the Gibbs free energy of the sensing elemental reaction, while facilitating oxygen species transfer through the spill-over effect. Furthermore, the dual-channel sensing mechanism of chemical and electron sensitization of the Ti_3_C_2_T_x_–MXene interfacially confined with N, C coordinated Ni single atoms promotes effective electron transfer to the 2D MXene conductive network, thereby amplifying the sensing signal of NH_3_ gas molecules. In addition, the MNPE–Ni–N–C/Ti_3_C_2_T_x_ gas sensor also demonstrates exceptional stability, especially with only a minor reduction in resistance and response (3.9% and 9.0%, respectively) over the 4 weeks compared to the AuE–Ni–N–C/Ti_3_C_2_T_x_ gas sensor (378.0% and 54.3%, respectively). This remarkable stability is primarily attributed to the end-sealing passivation effect on MXene edge defects induced by a conjugated hydrogen bond network of the organic solvent NMP and PEDOT:PSS. Moreover, the interface between the homogeneous MNPE and the Ni–N–C/Ti_3_C_2_T_x_ film exhibits excellent ohmic contacts, thereby reducing the SBH and improving the charge transfer ability. This study presents a groundbreaking approach for developing highly sensitive, selective, stable, and flexible gas sensors, which hold great promise for the early non-invasive diagnosis of respiratory diseases.

## Supplementary Information

Below is the link to the electronic supplementary material.

Supplementary file1 (DOCX 7671 KB)
