# Supplementary material for: Highly Sensitive Ammonia Gas Sensors at Room Temperature Based on the Catalytic Mechanism of N, C Coordinated Ni Single-Atom Active Center
Source: Nanomicro Lett. 2024 Aug 27;16:277. doi: 10.1007/s40820-024-01484-4 (PMC11349725; doi:10.1007/s40820-024-01484-4)
Supplement: Supplementary file 1 — Supplementary file1 (DOCX 7671 KB) [file 40820_2024_1484_MOESM1_ESM.docx]

Supporting Information for

**Highly Sensitive Ammonia Gas Sensors at Room Temperature Based on the Catalytic Mechanism of N, C Coordinated Ni Single-Atom Active Center**

Wenjing Quan^1, 2^, Jia Shi^1, 2^, Min Zeng^1,^ *, Wen Lv^1, 2^, Xiyu Chen^1, 2^, Chao Fan^1, 2^, Yongwei Zhang^1, 2^, Zhou Liu^1, 2^, Xiaolu Huang^1, 2^, Jianhua Yang^1, 2^, Nantao Hu^1, 2^, Tao Wang^3^, Zhi Yang^1,^ *

^1^National Key Laboratory of Advanced Micro and Nano Manufacture Technology, Shanghai Jiao Tong University, Shanghai, 200240, China

^2^Department of Micro/Nano Electronics, School of Electronic Information and Electrical Engineering, Shanghai Jiao Tong University, Shanghai, 200240, China

^3^Shanghai Key Laboratory of Intelligent Sensing and Detection Technology, School of Mechanical and Power Engineering, East China University of Science and Technology, Shanghai, 200237, China.

*Corresponding authors. E-mail: minzeng@sjtu.edu.cn (M. Zeng) and zhiyang@sjtu.edu.cn (Z. Yang)

**S1 Preparation Methods of Different Gas-Sensing Materials, MXene-Based Electrodes, and Fully Flexible Gas Sensors**

**S1.1 Preparation Methods of Different Gas-Sensing Materials**

***S1.1.1 Synthesis of ZIF-8, Ni-ZIF-8 and Ni_500_-ZIF-8 Precursors***

In a typical procedure, 2.19 g of MeIM (Bidepharm Co., Ltd., 98%) was dispersed in 67 mL methanol (Sinopharm Chemical Reagent Co., Ltd., ≥ 99.5%) and sonicated for 30 min, which was slowly dropped into 67 mL methanol containing 0.96 g of Zn(NO_3_)_2_·6H_2_O (Sinopharm Chemical Reagent Co., Ltd., ≥ 99.0%) under stirring at room temperature, followed by the ZIF-8 was grown under stirring treatment for 24 h. The dispersion was centrifuged and washed at 8000 rpm for 3 min with methanol several times and dried in a freeze dryer for 12 h, thus the ZIF-8 precursor was collected. Furthermore, for the preparation of Ni-N-C and Ni-NPs/NC, we also synthesized Ni-ZIF-8 and Ni_500_-ZIF-8, respectively, with different adsorbed Ni content using the same procedure, but an extra 49.43 mg of Ni(NO_3_)_2_·6H_2_O or 500.00 mg of Ni(NO_3_)_2_·6H_2_O was dissolved in 67 mL Zn(NO_3_)_2_·6H_2_O/methanol mixing solution under ultrasonic mixing for 30 min to form Ni-ZIF-8 or Ni_500_-ZIF-8, respectively.

***S1.1.2 Synthesis of*** ***Ti_3_C_2_T_x_ Gas-Ssensing Material***

The Ti_3_C_2_T_x_ gas sensing material was prepared by the liquid etching method. 1.0 g of LiF salt (Meryer Co., Ltd., 99.99%) was dispersed in 10 mL of 12.0 M HCl (Sinopharm Chemical Reagent Co., Ltd., 36%) and stirred for 30 min, then 1.0 g of Ti_3_AlC_2_ powder (400 mesh, purchased from 11 Technology Co., Ltd.) was slowly added to the mixture solution. After stirring at 35 °C for 24 h under a water bath, the obtained Ti_3_C_2_T_x_ suspension was centrifugated and washed at 8500 rpm for 5 min with DI water several times. The Ti_3_C_2_T_x_ precipitate was collected and dried in a freeze-dryer overnight to form the Ti_3_C_2_T_x_ gas sensing material.

***S1.1.3 Synthesis of Ni-ZIF-8-CTAB-Ti_3_C_2_T_x_ Precursor***

Briefly, 0.40 g of hexadecyl trimethyl ammonium bromide (CTAB, Sinopharm Chemical Reagent Co., Ltd., 99.0%) was dissolved in 20 mL methanol and sonicated for 30 min, which was added into 47 mL methanol containing 0.96 g of Zn(NO_3_)_2_·6H_2_O and 49.43 mg of Ni(NO_3_)_2_·6H_2_O (Sinopharm Chemical Reagent Co., Ltd., ≥ 99.0%) under stirring at room temperature to form solution A. In the meantime, 2.19 g of MeIM was dissolved into 67 mL methanol and sonicated for 30 min to form clear solution B. Then, solutions A and B were mixed under magnetic stirring. Stirring was continued after 2 h, and the 35 mL component solutions were collected to form solution C. 0.2 g of Ti_3_C_2_T_x_ gas sensing material was dissolved in 5 mL methanol under ultrasonic mixing for 30 min to form solution D. Then, solution D was slowly added into solution C with stirring for 24 h at room temperature. The obtained product was separated by centrifugation and washed subsequently with methanol three times and finally dried in a freeze dryer for 12 h.

***S1.1.4 Synthesis of*** ***N-C, Ni NPs/N-C, Ni-N-C and Ni-N-C/Ti_3_C_2_T_x_ Materials***

The ZIF-8, Ni_500_-ZIF-8, Ni-ZIF-8, and Ni-ZIF-8-CTAB-Ti_3_C_2_T_x_ precursors were placed in a tube furnace and subsequently heated to 850 °C (heating rate 5 ℃/min) in a stream of argon gas for 2 h to yield the final N-C, Ni NPs/N-C, Ni-N-C, and Ni-N-C/Ti_3_C_2_T_x_ materials, respectively.

**S1.2 Preparation Methods of Different MXene-Based Electrodes**

***S1.2.1 Synthesis of the Aqueous Ti_3_C_2_T_x_-MXene Electrode (ME) Ink and the Hybrid Ti_3_C_2_T_x_-MXene/NMP/PEDOT:PSS Electrode (MNPE) Ink***

The etchant consisted of 1.6 g of LiF salt, which was dissolved in 40 mL of 9.0 M HCl and pre-stirred for 30 min. Subsequently, 1.0 g of Ti_3_AlC_2_ powder was gradually introduced into the etchant. The resulting mixture was stirred for 24 h in a 35 °C water bath. The as-prepared Ti_3_C_2_T_x_ dispersion was then subjected to centrifugation and washed with DI water at 8500 and 3500 rpm repeatedly until the pH of the supernatant reached 6, yielding the Ti_3_C_2_T_x_ electrode material. After decanting the supernatant, 9 mL of NMP (Meryer Co., Ltd., 99.5%) and 1 mL PEDOT:PSS (CLEVIOS PH1000, from Heraeus) was added to the Ti_3_C_2_T_x_ sediment, and the dispersion was sonicated for 2 h. The well-dispersed flakes were then separated from the aggregated platelets by centrifugation at a low speed (1500 rpm, 30 min). The supernatant was left overnight and then continued further collection of the supernatant. After that, the supernatant was vigorously sonicated at 400 W for 4 h to obtain the MNPE ink. Furthermore, we also synthesized the ME ink with 10 mL of DI water through the same procedure.

***S1.2.2 Inkjet-Printing of ME and MNPE***

The two inks were inkjet printed using a Hewlett-Packard Laserjet P1007 printer on the paper substrate. The printer was fitted with the cartridge, preparing the flexible paper-based ME by dropping the ME ink into it and continuously inkjet-printing 10 times. What’s more, the flexible MNPE was printed by alternate inkjet-printing of ME ink and MNPE hybrid ink on paper for a total of 10 times.

**S1.3 Fabrication of Fully Flexible Gas Sensors**

After that, 20 μL ethanol dispersion containing 10 mg of different gas sensing materials was dropped on the AuE, ME, and MNPE to fabricate a variety of gas sensors. These devices were dried at 60 °C for 12 h in a vacuum drying oven.

**S1.4 Materials Characterization**

The morphology of the samples was determined by SEM (Carl Zeiss Ultra Plus, Germany) and TEM (HT7000 Hitachi, Tokyo, Japan). The element compositions and structure of each sample were identified using XRD (D8 ADVANCE, Bruker, Germany) with Cu Kα diffraction (λ = 0.154056 nm). The elemental composition and bond structures of synthesized samples were analyzed using a Kratos Axis Ultra DLD (Kratos Analytical, UK) XPS system equipped with a monochromatic Al Kα source. The surface area and the pore size distribution of the samples were obtained by the N_2_ isothermal adsorption/desorption at 77 K on a Micromeritics TriStar II 3020 analyzer (Micromeritics, USA). The HAADF-STEM and EDS images of dispersed Ni over the N-C framework were captured on a JEOL ARM200F (JEOL, Japan) with a 200 kV operation voltage. The XAFS spectra at the Ni K-edge were conducted at the X-ray absorption fine structure for catalysis (XAFCA) beamline at the Singapore Synchrotron Light Source (SSLS) in fluorescence mode. Ni foil and NiO served as reference materials. The EXAFS data were acquired following standard procedures based on the ATHENA module within the IFEFFIT software packages. Quantitative analysis of the samples was performed through least-squares curve fitting in the R space, employing a Fourier transform k space range from 2.4 to 10.4 Å⁻^1^, with the ARTEMIS module of the IFEFFIT programs. Fourier transform infrared (FTIR) spectroscopy was performed on Nicolet 6700 FTIR spectrometers (Thermo Nicolet, Madison, WI) with a spectral range of 400 to 4000 cm^−1^. Raman analysis was obtained by using a confocal InVia Raman microscope (England, Renishaw) with the 633 nm laser. TGA analyses of samples were conducted on an SDT-Q600 thermogravimetric analyzer (New Castle, DE, USA) at a heating rate of 5 °C min⁻^1^ in Ar. The contents of Ni in the samples were obtained by the ICP-MS iCAP Q (Thermo Fisher Scientific, Waltham, USA). The rheological properties of ME, as well as MNPE inks, were studied on the rheometer (Anton Paar Physica MCR 301, Graz, Austria). The electrical conductivity of the as-printed electrodes was tested through a four-point probe technique (RTS-11, Guangzhou Four Probe Co., Ltd.). The band gap of samples was assessed via UV-vis spectroscopy (Lamda 950, PerkinElmer, USA). The hydrodynamic diameter and the zeta potential measurements of the Ti_3_C_2_T_x_-based electrode inks were carried out by NanoBrook 90Plus PALS analyzer (Brookhaven, USA). The static contact angles and the surface tension of the electrode inks were evaluated using a Kruss DSA30S analyzer (Kruss, Germany). The valence band and the position of the Fermi level were investigated using UPS (Kratos Axis Ultra DLD, UK).

**S2 Supplementary Figures and Tables**


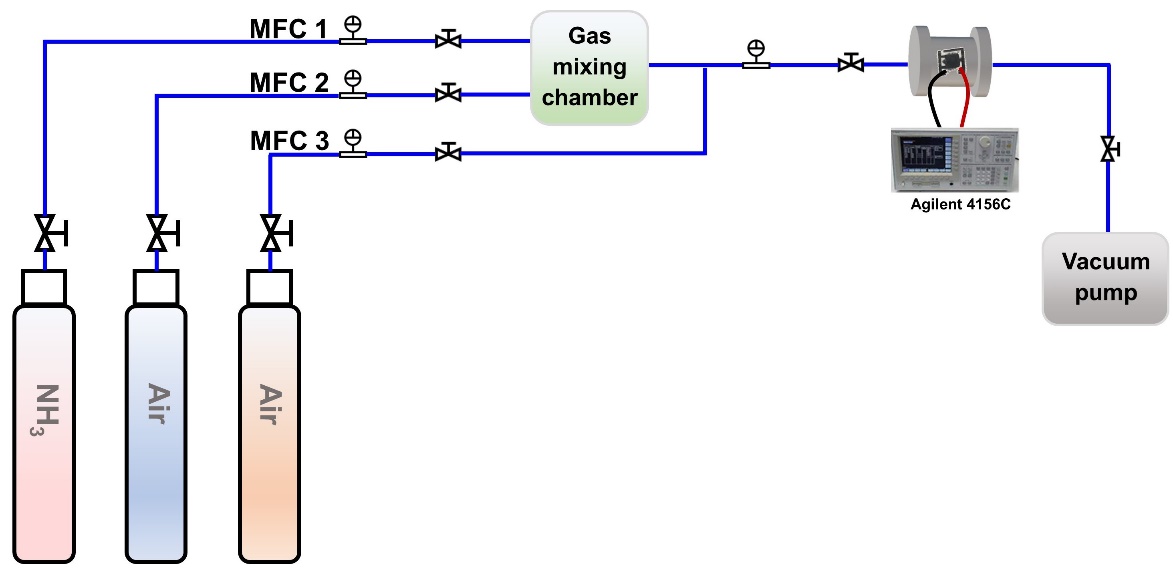


**Fig. S1** Schematic diagram of the gas sensing performance test system.


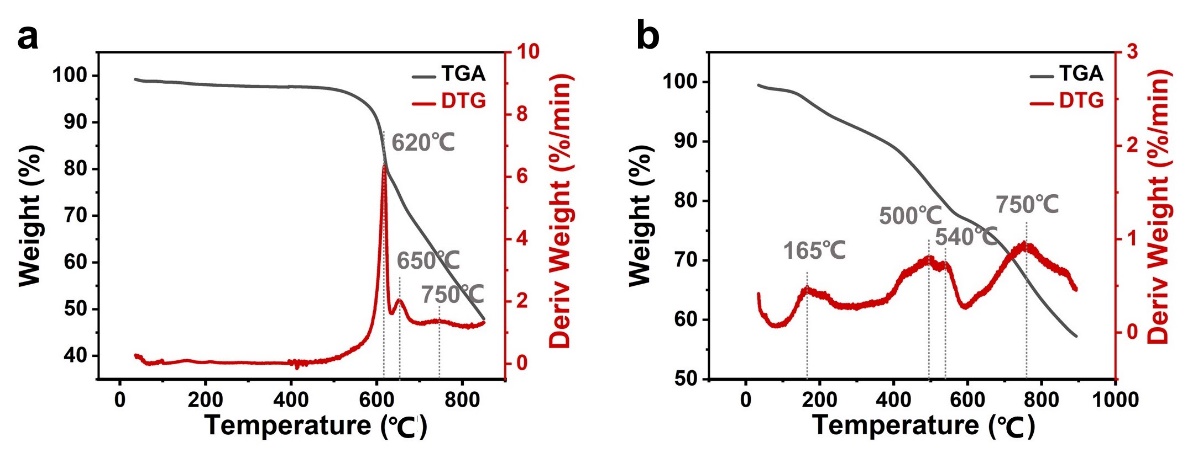


**Fig. S2 The TGA–DTG of Ni-ZIF-8 and Ni-ZIF-8-CTAB-Ti_3_C_2_T_x_ precursor.** TGA (grey) and its corresponding first-order differential curve (red) of **a** Ni-ZIF-8 and **b** Ni-ZIF-8-CTAB-Ti_3_C_2_T_x_ precursors under Ar.

The typical weight losses at about 500–650 °C were attributed to the organic linkers decomposition of Ni-ZIF-8 and Ni-ZIF-8-CTAB-Ti_3_C_2_T_x_ which were transformed into N-C skeletons. When the temperature rose to over 750 °C, the Zn species with a low boiling point were released leaving N-rich defects [S1]. It is worth noting that in contrast to Ni-ZIF-8, there was a typical weight loss at about 165 °C in Ni-ZIF-8-CTAB-Ti_3_C_2_T_x_ which was attributed to the carbonization of CTAB [S2].


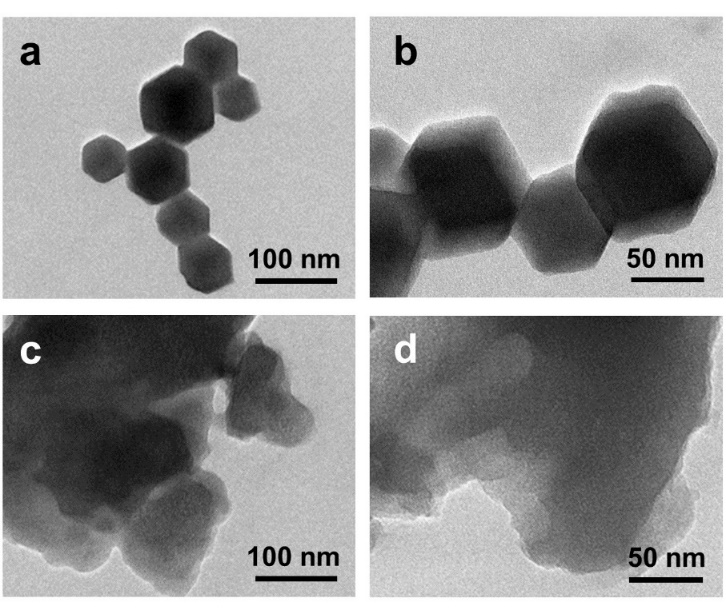


**Fig. S3 The structure characterizations of ZIF-8 and N-C catalysts.** **a–b** TEM images of the ZIF-8 material. **c–d** TEM images of the N-C material.

The initial ZIF-8 and Ni-ZIF-8 precursors exhibited a uniform rhombododecahedral shape and size distribution with an average size of 80 nm (**Figs. S3a–b and S4a–b**).


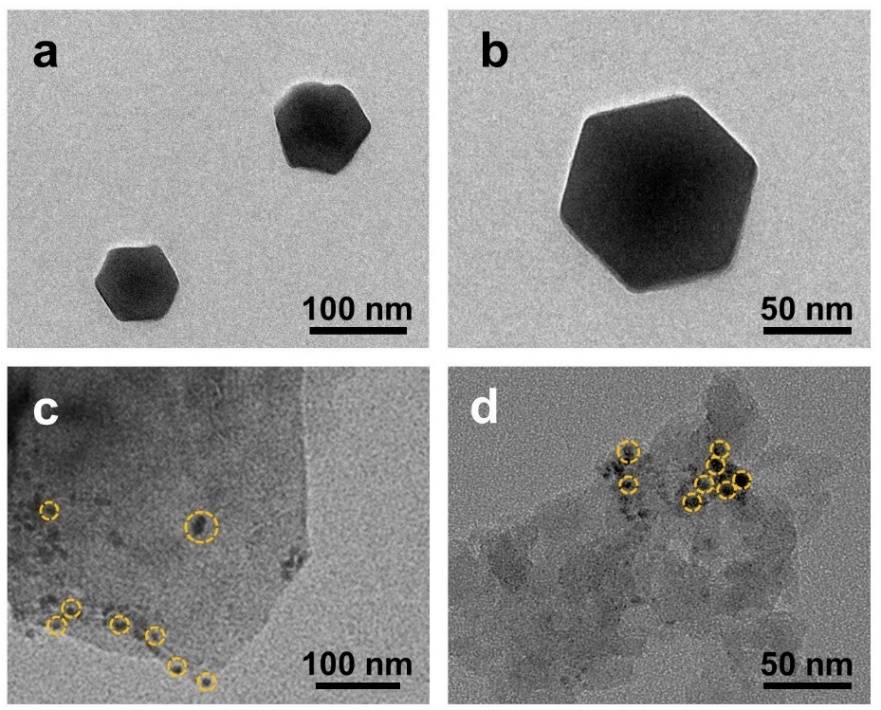


**Fig. S4 The structure characterizations of Ni-ZIF-8 and Ni NPs/N-C catalysts.** **a–b** TEM images of the Ni-ZIF-8 material. **c–d** TEM images of the Ni NPs/N-C material.


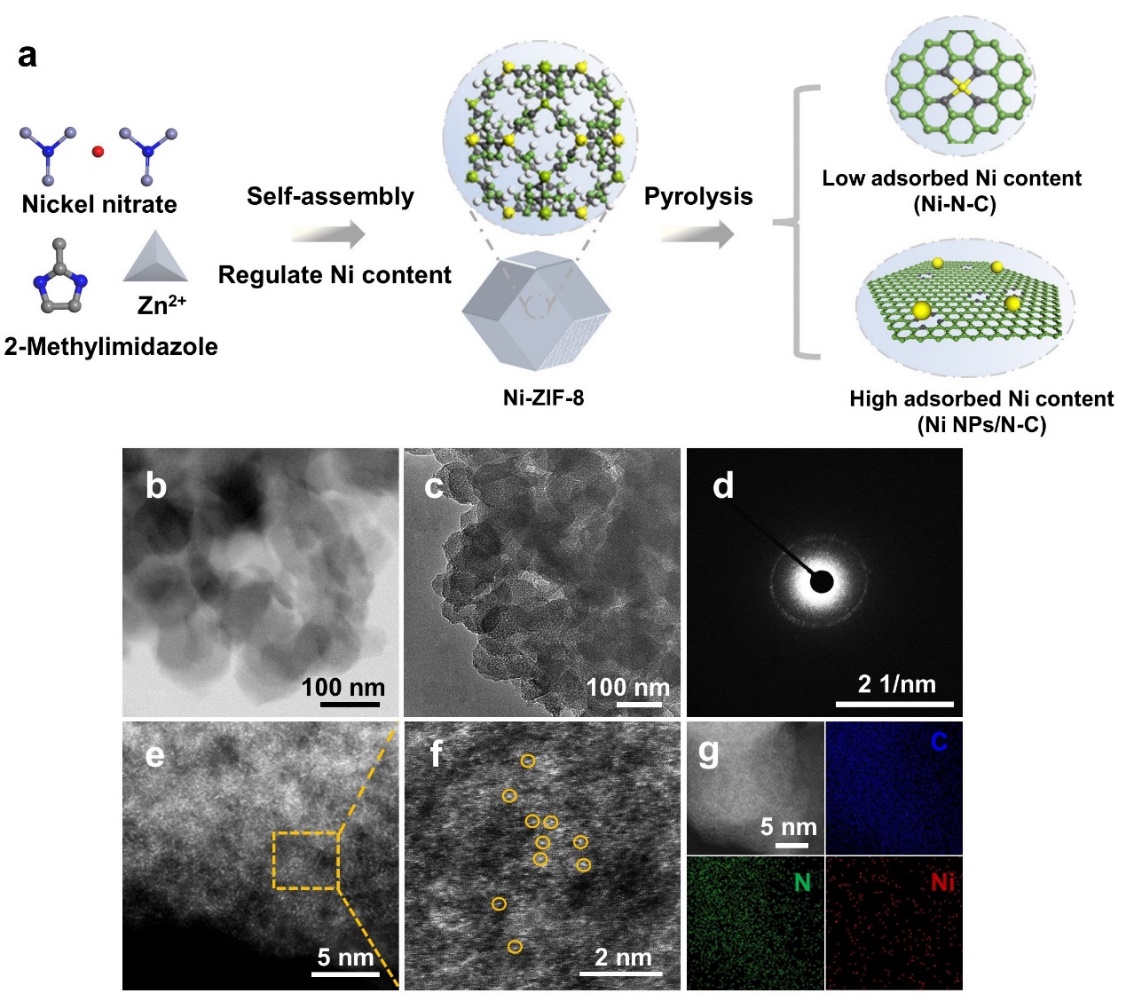


**Fig. S5** **The preparation process and** **structure characterizations** **of Ni-N-C.** **a** The preparation process of Ni-N-C. **b–c** The HAABF-STEM and TEM images of Ni-N-C catalyst. **d** Corresponding selected-area electron diffraction (SAED) pattern of Ni-N-C catalyst. **e–f** The HAADF-STEM images of Ni-N-C catalyst. **g** The corresponding EDS for carbon (C), nitrogen (N), and nickel (Ni) elements of Ni-N-C catalyst.

Furthermore, by regulating the content of adsorbed Ni^2+^ and the number of anchoring sites, thus the Ni NPs/N-C and Ni-N-C formed during the pyrolysis (**Figs. S4 and S5**). The resulting Ni-NPs/N-C and Ni-N-C catalysts lose the protection of CTAB, and the dense and homogeneous rhombododecahedral morphology of the Ni-ZIF-8 precursor cannot be well-retained (**Fig. S4c**). There are significant Ni clusters on the N-C substrate surface in the sample of Ni NPs/N-C displayed in **Fig. S4d**. Furthermore, the Ni-N-C structure has poor crystallinity demonstrated in the SAED pattern (**Fig. S5d**). The HAADF-STEM images (**Fig. S5e, f**) and the EDS mapping results (**Fig. S5g**) of Ni-N-C suggest that the Ni species on the N-C surface dominantly exhibit atomic dispersion and Ni, N, and C are homogeneously dispersed on the whole structure.


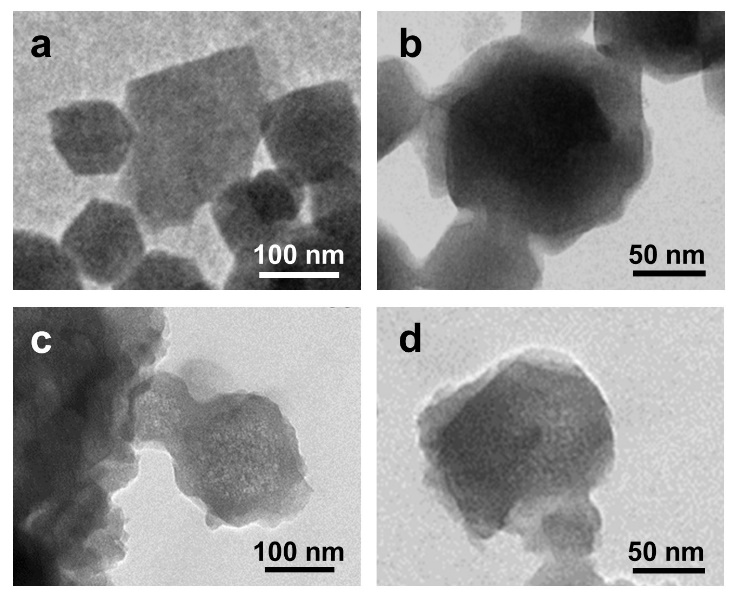


**Fig. S6 The structure characterizations of** **Ni-ZIF-8-CTAB-Ti_3_C_2_T_x_ precursor and** **Ni-N-C/Ti_3_C_2_T_x_ catalyst. a–b** TEM images of the Ni-ZIF-8-CTAB-Ti_3_C_2_T_x_ precursor. **c–d** TEM images of the Ni-N-C/Ti_3_C_2_T_x_ catalyst.

Compared to the shape of the ZIF-8 and Ni-ZIF-8 precursor, a hybrid nanostructure is observed in the Ni-ZIF-8-CTAB-Ti_3_C_2_T_x_ precursor (**Fig. S6a and b**), which consisted of the stacked 2D structure of the Ti_3_C_2_T_x_ membrane, a surfactant CTAB polymer and Ni-ZIF-8 structure with rhombododecahedral shape, indicating that the addition of CTAB-surfactant did not influence the crystalline structure of the Ni-ZIF-8 and Ti_3_C_2_T_x_. It is worth noting that due to the capping ability of CTAB-surfactant, the particle sizes of the Ni-ZIF-8 particles in the Ni-ZIF-8-CTAB-Ti_3_C_2_T_x_ precursor decreased obviously.


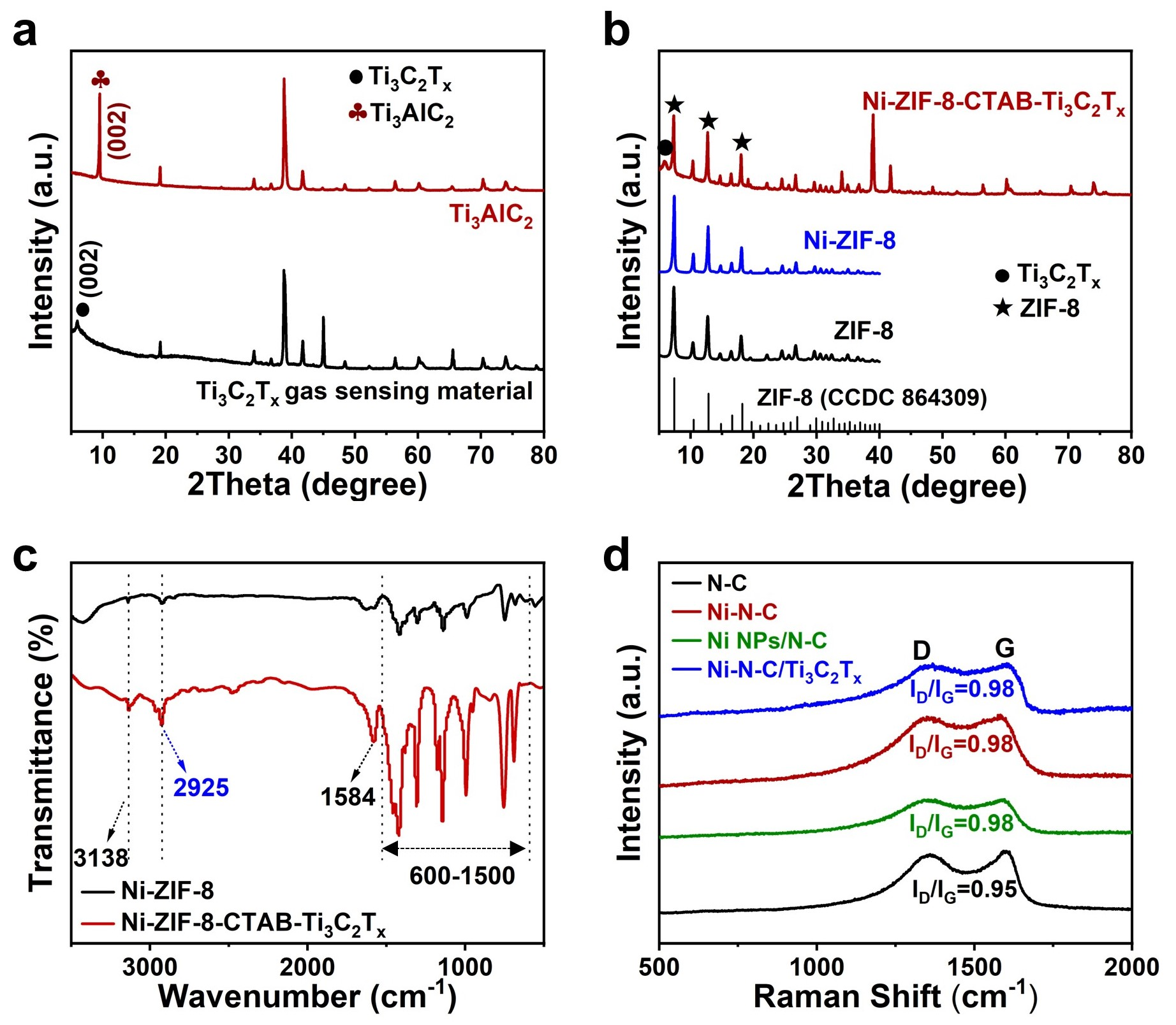


**Fig. S7 The structural and Raman analysis of Ti_3_C_2_T_x_ gas sensing material, Ni-ZIF-8-CTAB-Ti_3_C_2_T_x_ precursor, N-C, Ni-N-C, Ni NPs/N-C, and Ni-N-C/Ti_3_C_2_T_x_ catalysts. a** XRD spectra of the Ti_3_AlC_2_ and Ti_3_C_2_T_x_ gas sensing material. **b** XRD spectra of ZIF-8, Ni-ZIF-8, and Ni-ZIF-8-CTAB-Ti_3_C_2_T_x_ precursors. **c** The Fourier transform infrared (FTIR) spectra of Ni-ZIF-8 and Ni-ZIF-8-CTAB-Ti_3_C_2_T_x_ precursors. **d** Raman spectra of N-C, Ni-N-C, Ni NPs/N-C, and Ni-N-C/Ti_3_C_2_T_x_ samples.

After etching with HCl and LiF, the XRD peak of Ti_3_C_2_T_x_ gas sensing material corresponding to the (002) plane moved from 9.5° to 6.0° in **Fig. S7a**, due to the etched Al atom layers and the introduction of the surface functional group [S3], which indicated the successful synthesis of Ti_3_C_2_T_x_ gas sensing material. Furthermore, the XRD pattern of Ni-ZIF-8 was matched with that of ZIF-8 and observed no obvious characteristic peaks corresponding to Ni, since the content of Ni was too low to be detected (**Fig. S7b**). Furthermore, the absorption in Fourier Transform Infrared Spectroscopy (FTIR) at 600–1500 cm^−1^, 1584 cm^−1^, and 3138 cm^−1^, which could be attributed to the vibration of the imidazole units, the stretching vibration of C=N band, and C–H bond of imidazole rings, respectively, were present in Ni-ZIF-8 and Ni-ZIF-8-CTAB-Ti_3_C_2_T_x_ samples (**Fig. S7c**) [S4]. Furthermore, the fluctuation in the intensity of the 2925 cm^−1^ characteristic peak in the absorption in FTIR spectra assigned to the stretching vibration signals of C–H bonds of methyl in the samples showed that compared to the Ni-ZIF-8 precursor, Ni-ZIF-8-CTAB-Ti_3_C_2_T_x_ had distinct long-chain alkanes structure, corresponding to the CTAB surfactant [S5]. Raman spectra (**Fig. S7d**) indicated that all catalysts exhibited similar carbon structures with dominant D and G bands at 1350 and 1590 cm^−1^, regardless of the Ni doping level, associated with the disordered carbons and sp^2^ hybridized graphitic carbons, respectively. The Raman spectra of Ni-N-C, Ni-N-C/Ti_3_C_2_T_x_, and Ni NPs/N-C demonstrated a relatively high I_D_/I_G_ value of 0.98, indicating larger amounts of defects than N-C material [S6].


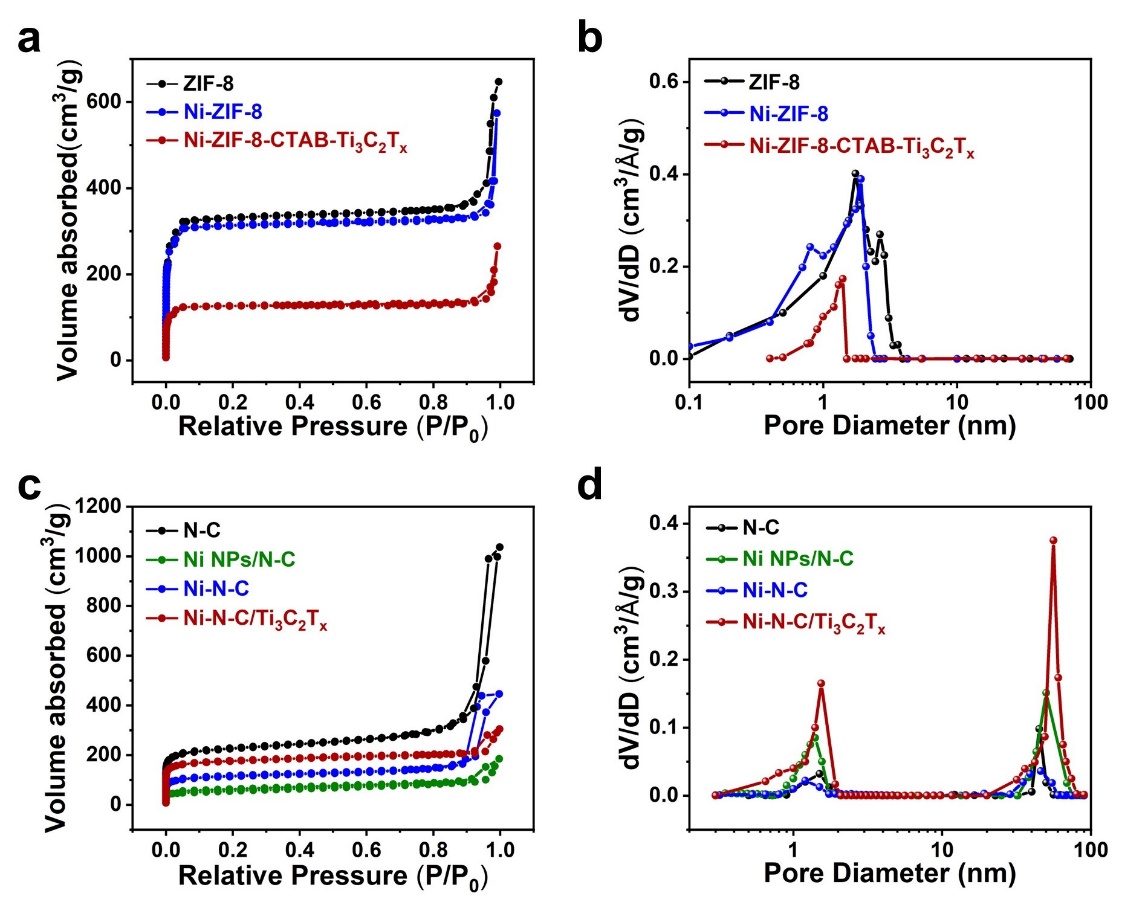


**Fig. S8 The nitrogen adsorption-desorption analysis of precursors and calcined products.** **a** N_2_ adsorption-desorption curves of ZIF-8, Ni-ZIF-8, and Ni-ZIF-8-CTAB-Ti_3_C_2_T_x_ precursors. **b** The corresponding pore size distributions of ZIF-8, Ni-ZIF-8, and Ni-ZIF-8-CTAB-Ti_3_C_2_T_x_ precursors. **c** N_2_ adsorption-desorption curves of N-C, Ni-N-C, Ni NPs/N-C, and Ni-N-C/Ti_3_C_2_T_x_ samples. **d** The corresponding pore size distributions of N-C, Ni-N-C, Ni NPs/N-C, and Ni-N-C/Ti_3_C_2_T_x_ samples.

N_2_ adsorption/desorption analysis indicated that, for all precursors, the adsorption isotherm generated resembled type I isotherm, which suggested a microporous structure with a similar Brunauer-Emmett-Teller (BET) surface area of around 300–1000 m^2^g^−1^ (**Fig. S8a, b, and Table S2**) [S7]. After pyrolysis, the surface areas of all compounds showing type IV isotherm decreased to 180–700 m^2^g^−1^ with increased mesopores and macropores (**Fig. S8c and d**) [S8].


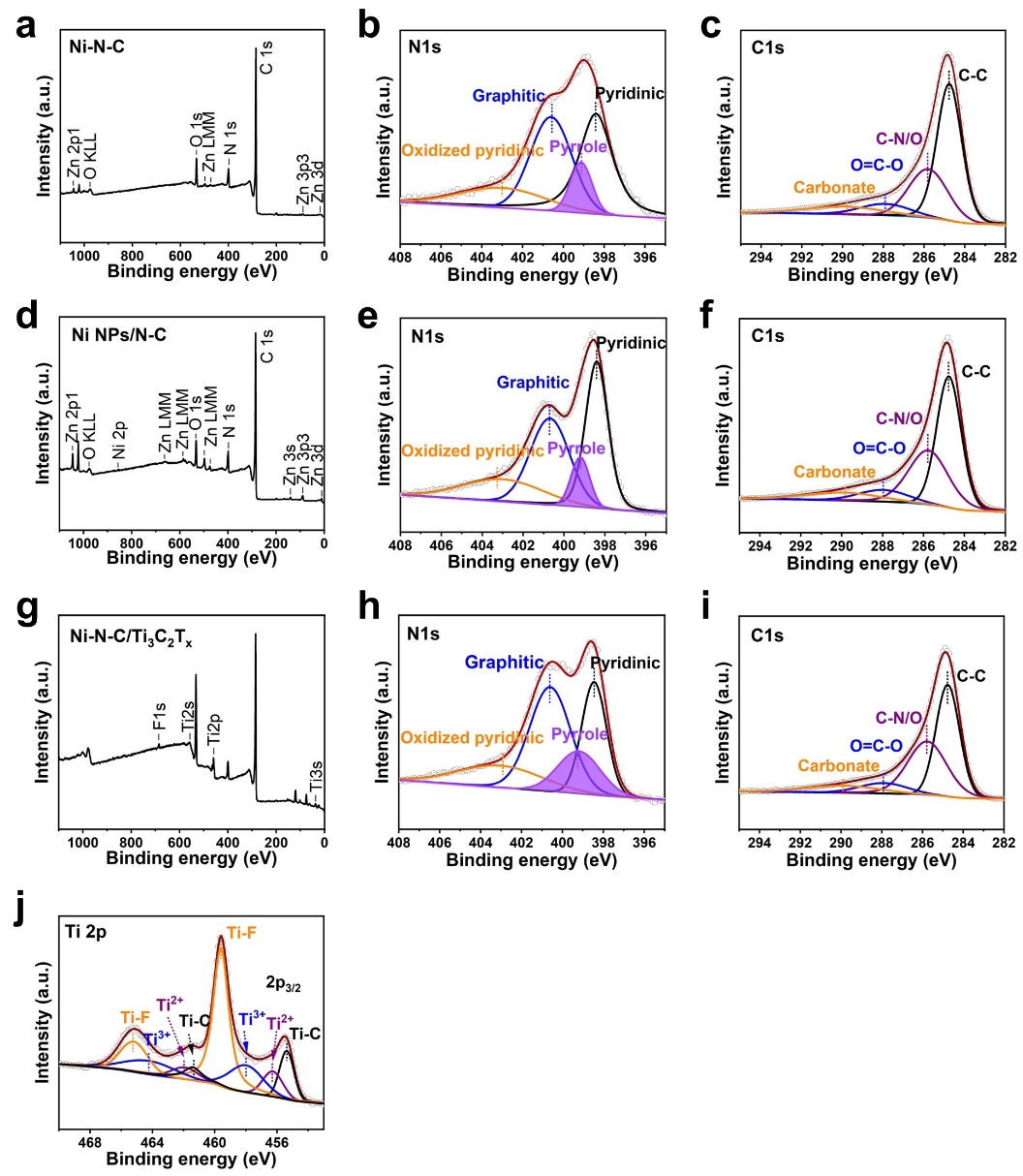


**Fig. S9 The XPS analysis of Ni-N-C, Ni NPs/N-C, and Ni-N-C/Ti_3_C_2_T_x_ materials.** XPS spectra for **a** survey scan, **b** N 1s, and **c** C 1s regions of Ni-N-C. XPS spectra for the **d** survey scan, **e** N 1s, and **f** C 1s regions of Ni NPs/N-C. XPS spectra for the **g** survey scan, **h** N 1s, **i** C 1s, and **j** Ti 2p regions of Ni-N-C/Ti_3_C_2_T_x_.

The C 1s spectra of the three catalysts exhibited four main components corresponding to C-C (284.8 eV), C-N/O (285.8 eV), O=C-O (287.9 eV), and carbonate (289.7 eV) in **Fig. S9c, f, and i**. From the Ti 2p spectrum of Ni-N-C/Ti_3_C_2_T_x_ nanohybrids, the binding energies of Ti 2p_3/2_ for Ti-C, Ti^2+^, Ti^3+^, and Ti-F are 455.4, 456.3, 458.0, and 459.6 eV, respectively, in agreement with previous XPS studies (**Fig. S9j**) [S9].


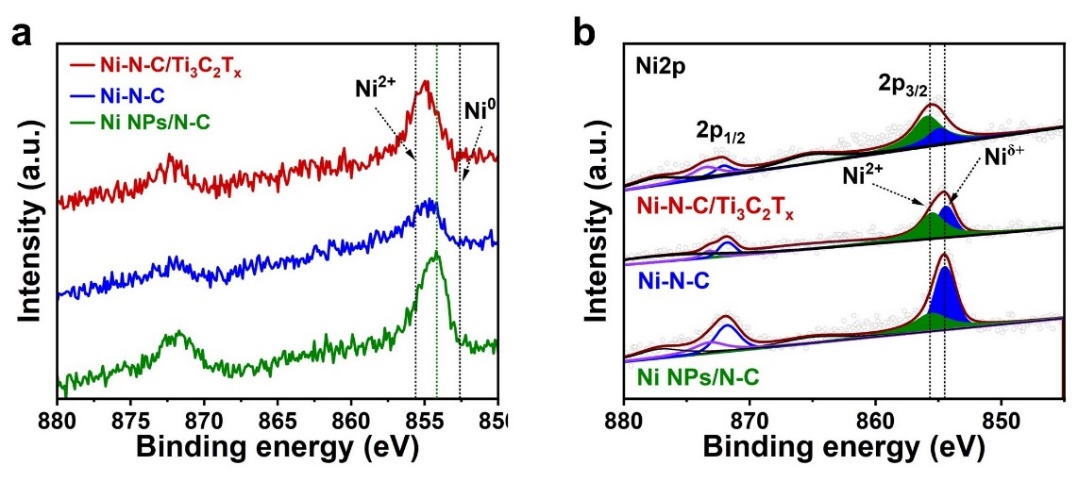


**Fig. S10 The XPS analysis of Ni-N-C, Ni NPs/N-C, and Ni-N-C/Ti_3_C_2_T_x_ materials.** **a–b** XPS spectra for the Ni 2p regions of Ni-N-C, Ni NPs/N-C, and Ni-N-C/Ti_3_C_2_T_x_ catalysis.


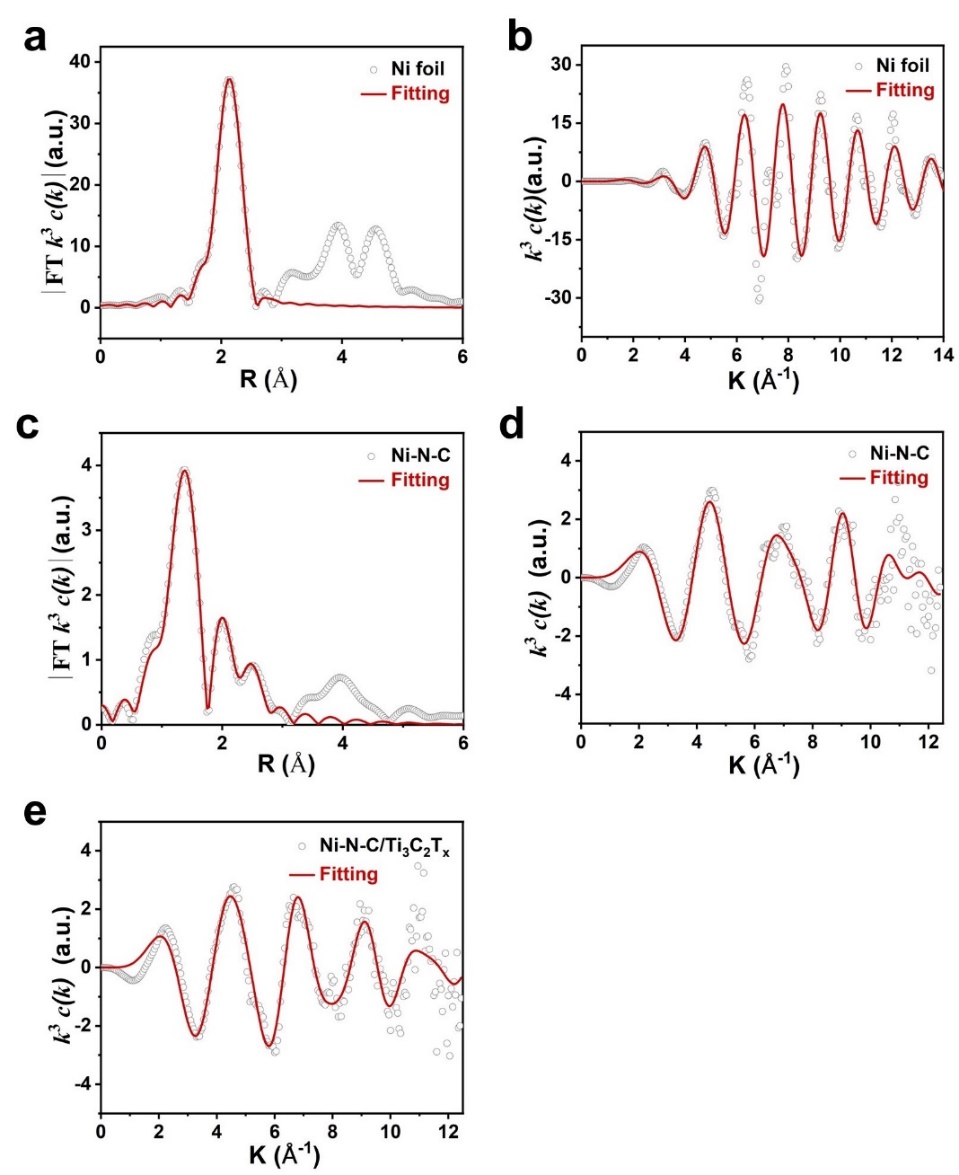


**Fig. S11 The EXAFS analysis of Ni foil, Ni-N-C, and Ni-N-C/Ti_3_C_2_T_x_ materials.** Corresponding EXAFS fitting curves for Ni foil **a** R-space and **b** K-space. Corresponding EXAFS fitting curves for Ni-N-C **c** R-space and **d** K-space. **e** Corresponding EXAFS fitting curves for Ni-N-C/Ti_3_C_2_T_x_ K-space.


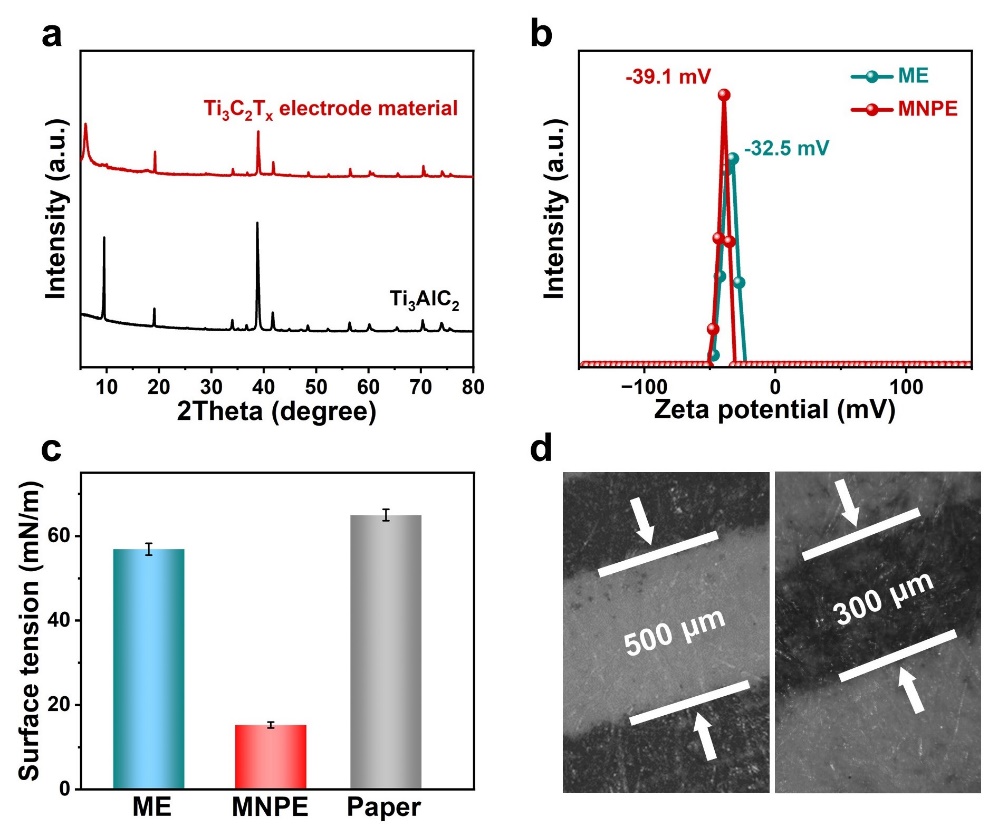


**Fig. S12 The Ti_3_C_2_T_x_-based inks analysis.** **a** XRD of Ti_3_AlC_2_ and Ti_3_C_2_T_x_ electrode materials. **b** zeta potential of ME and MNPE inks. **c** The surface tension of paper substrate, ME ink, and MNPE ink. **d** Optical microscope images of the ME lines.

The paper substrate surface energy of 62 mN/m was higher than the surface tension of ME and MNPE inks, emphasizing the importance of substrate selection in achieving high-resolution printing of Ti_3_C_2_T_x_-based inks (**Fig. S12c**).


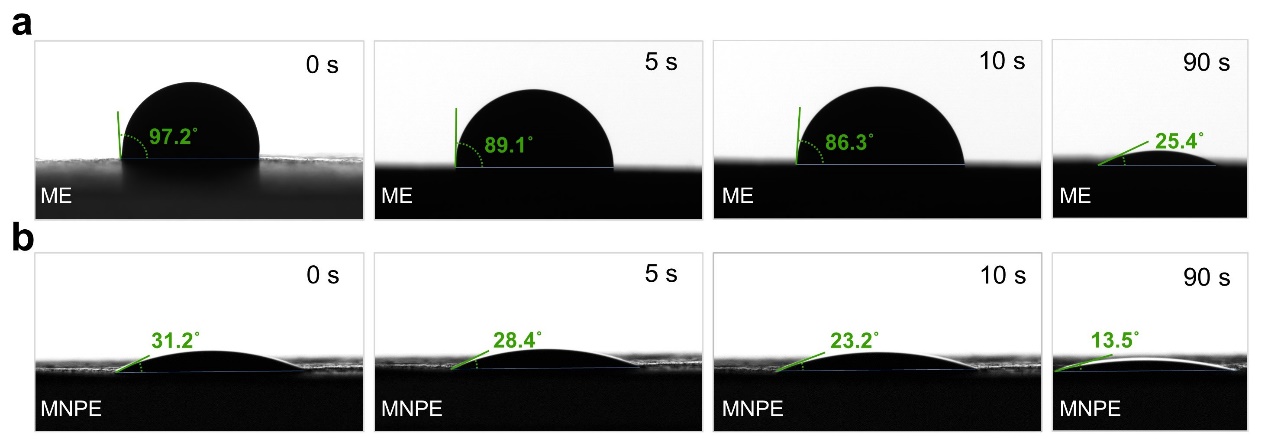


**Fig. S13 The contact angles analysis of Ti_3_C_2_T_x_-based inks.** **a** Contact angles of ME ink and paper substrate. **b** Contact angles of MNPE ink and paper substrate.

Furthermore, the small contact angles (≪ 90°) with increasing infiltration treatment time indicated good wetting properties between the inks and paper substrate (**Fig. S13**).


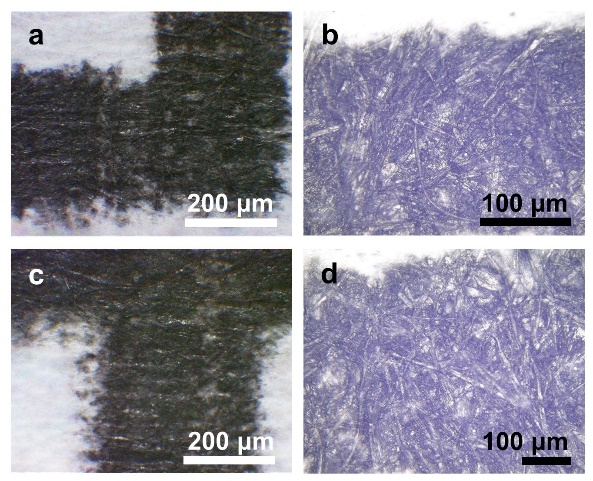


**Fig. S14 The optical images of Ti_3_C_2_T_x_-based electrode films.** Optical microscope images of the **a–b** ME and **c–d** MNPE films.

Both Ti_3_C_2_T_x_-based printed films were uniform without the coffee-ring effect (**Fig. S14**), demonstrating good printability of ME and MNPE inks.


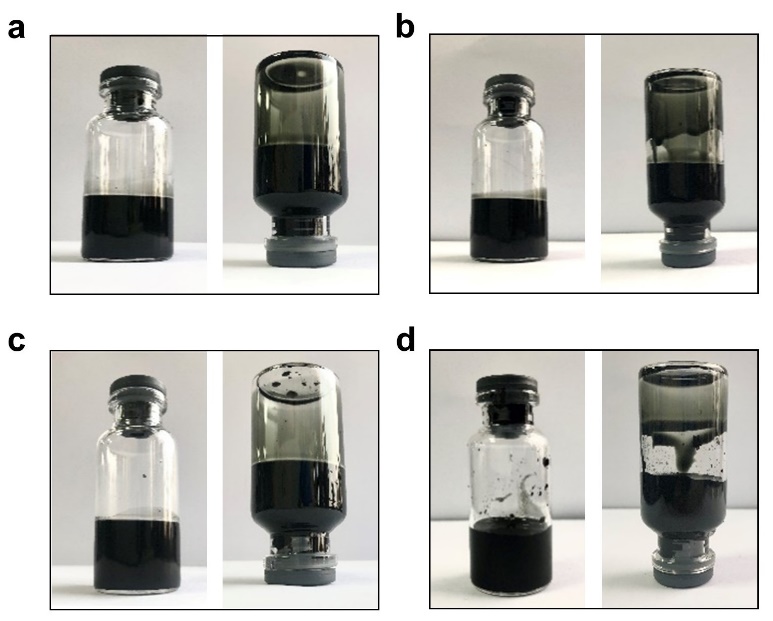


**Fig. S15 The stability analysis of ME and MNPE inks.** Dispersions of ME ink were monitored at time points **a** after ultrasound treatment and **b** after 10 days. Dispersions of MNPE ink were monitored at time points **c** after ultrasound treatment and **d** after 10 days.

The two Ti_3_C_2_T_x_-based inks which had excellent solution processibility, appropriate viscosity, and surface energy well-matched with the substrates, had a great degree of stability that could be stable in air for 10 days, excellent conductivity, good printing adaptability, and enough versatility to design ideal patterns (**Figs. S15–17**).


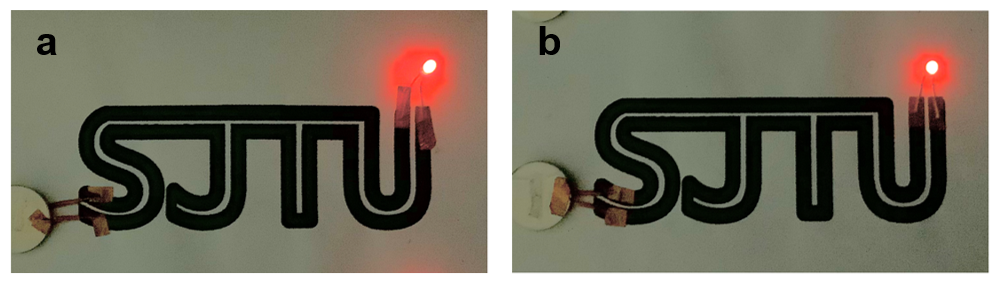


**Fig. S16 The conductivity and printing adaptability analysis of ME and MNPE films.** Inkjet-printed school abbreviation SJTU (Shanghai Jiao Tong University) by **a** ME and **b** MNPE inks on paper substrate.

Two different inks were printed using an inkjet printer on paper substrates to form the “SJTU” shape and light-emitting diodes (LEDs) could be lit by both two films (**Fig. S16a and b**).


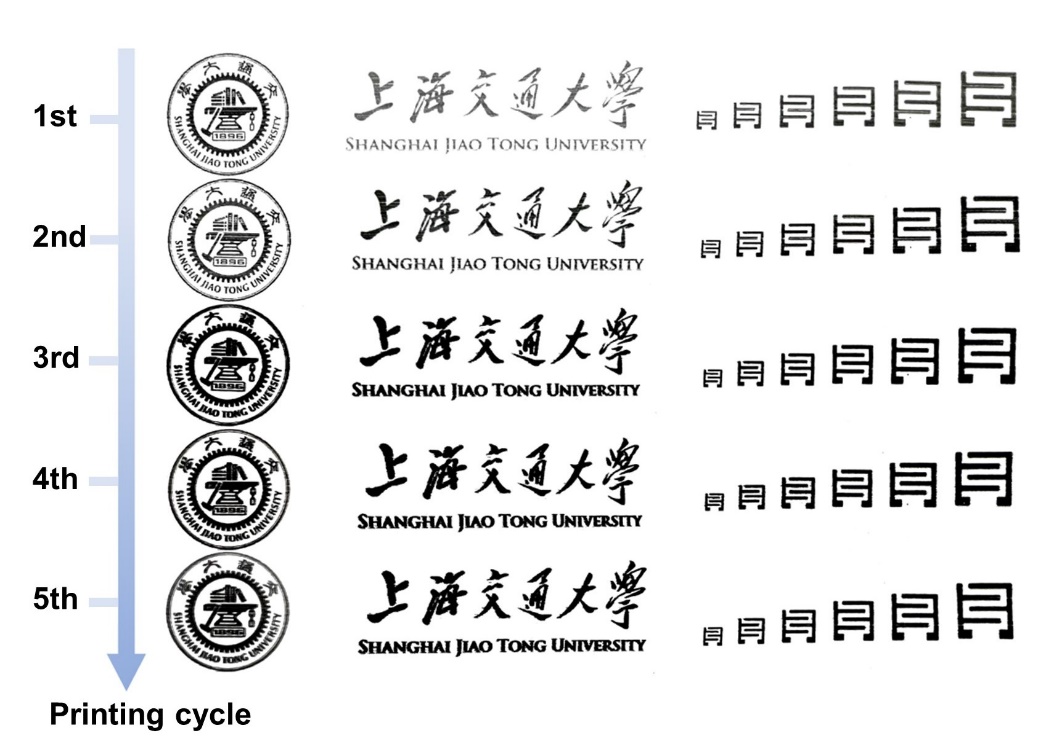


**Fig. S17 The optical images of different patterns.** Optical images of the school badges and interdigital electrodes printed by inkjet under different printing cycles.

This maskless inkjet printing technique supports a variety of pattern fabrication, paving the way for customizable geometries for gas sensors.


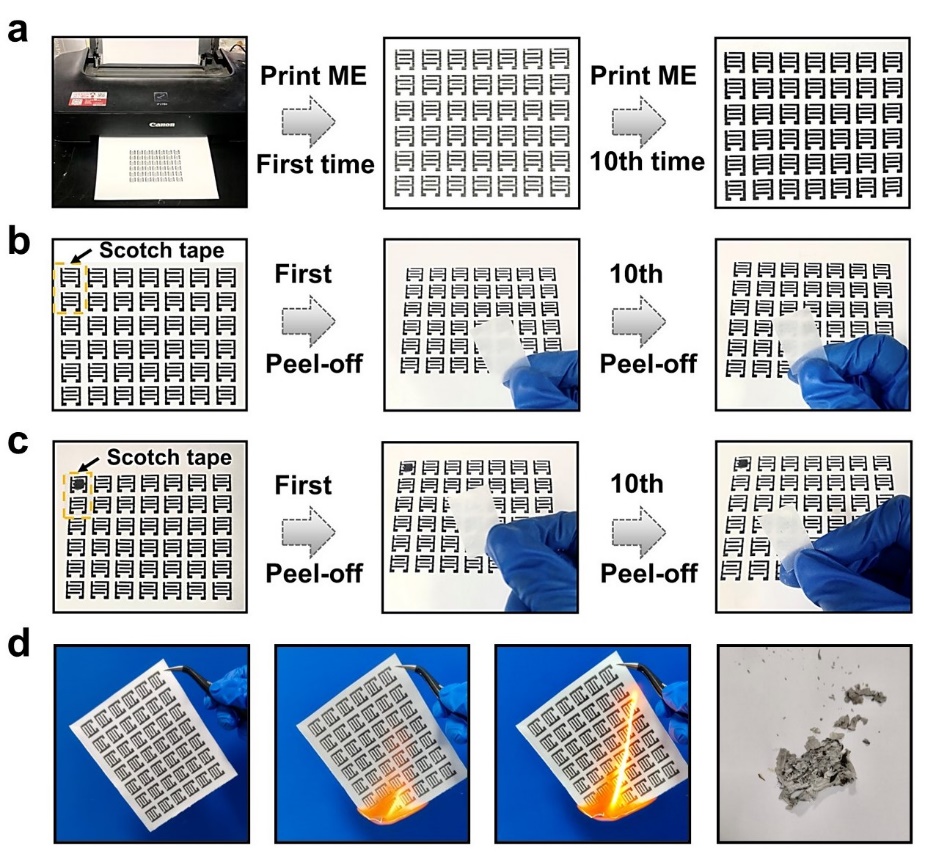


**Fig. S18 The optical images of different patterns.** **a** Optical images of paper-based ME array with the increase of printing cycles. The adhesivity of the paper-based **b** ME array and **c** integrated ME-Ni-N-C/Ti_3_C_2_T_x_ gas sensor tested by scotch tape. **d** The schematic diagram of burning the paper-based ME array.

The optical images of the paper-based ME array depicted an increase in the proportion of the surface ME ink as the printing cycles increased, finally, ME ink was evenly distributed over the paper surface (**Fig. S18a**). Additionally, the printed ME and ME-Ni-N-C/Ti_3_C_2_T_x_ gas sensor exhibited excellent structural integrity without any delamination from the substrate under sticking by cellophane tapes (**Fig. S18b and c**), and the combustion process proved that the flexible ME was eco-friendly (**Fig. S18d**).


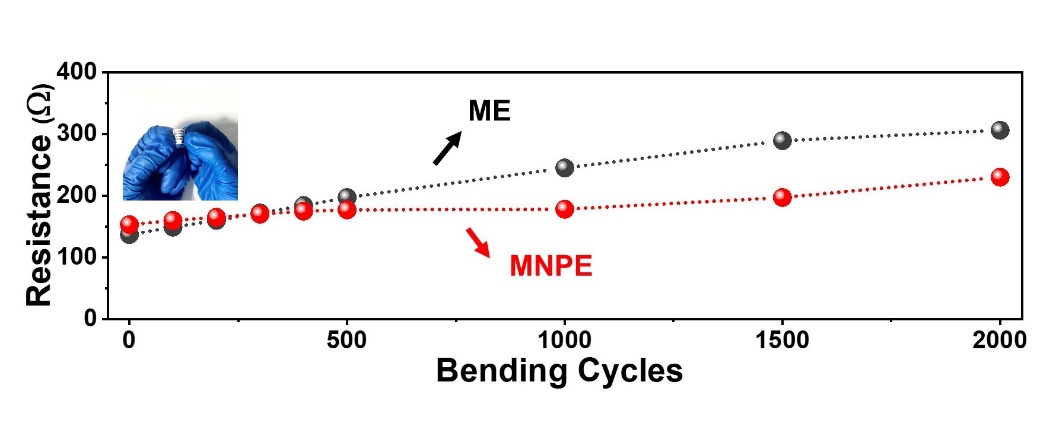


**Fig. S19 The mechanical deformation analysis of ME and MNPE inks.** The functional relationship between the relative change of resistance and the bending cycle of the ME and MNPE.


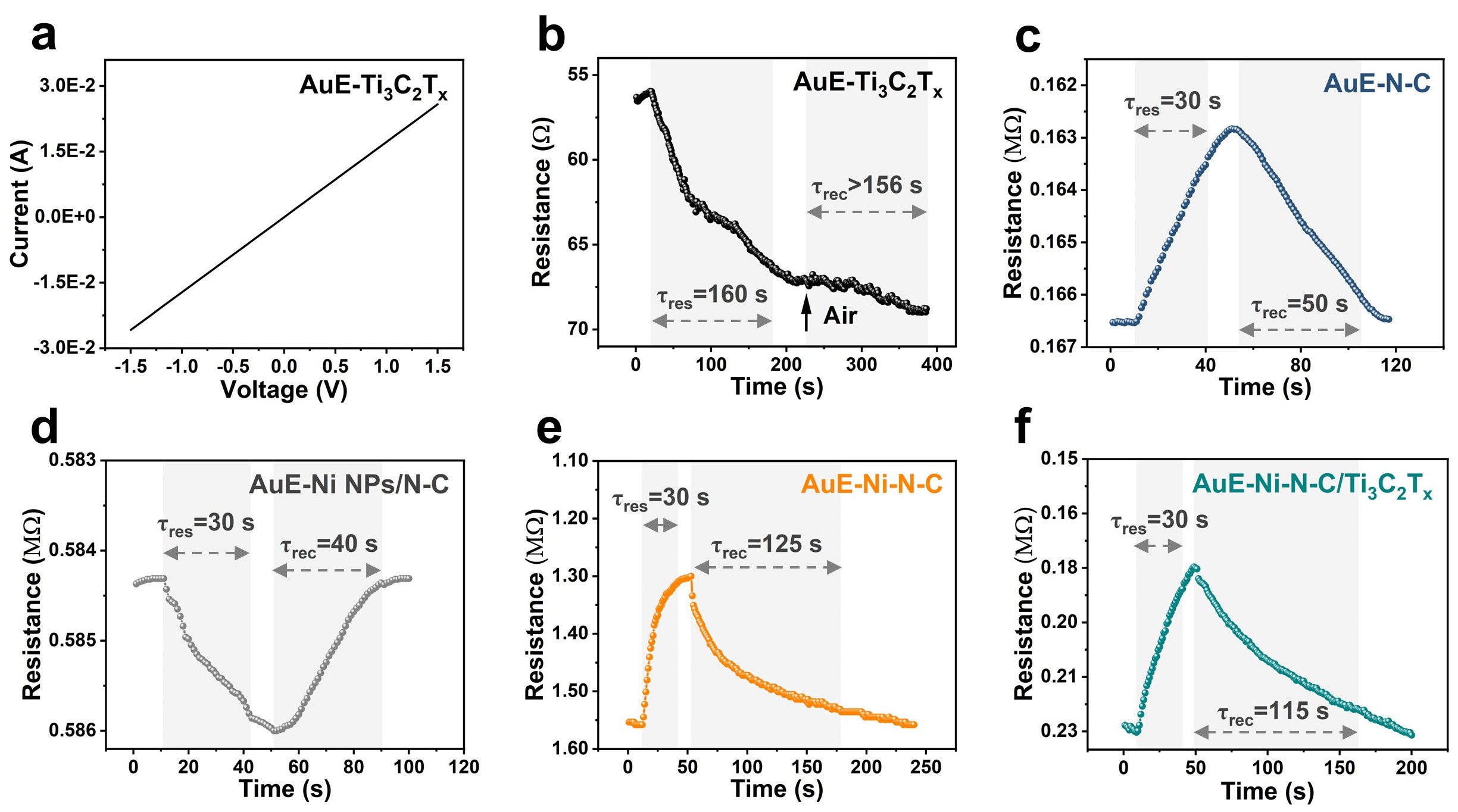


**Fig. S20 Performance of different gas sensors for low-concentration NH_3_ detection.** **a** *I-V* curves of AuE-Ti_3_C_2_T_x_ sensor. **b** AuE-Ti_3_C_2_T_x_, **c** AuE-N-C, **d** AuE-Ni NPs/N-C, **e** AuE-Ni-N-C, and **f** AuE-Ni-N-C/Ti_3_C_2_T_x_ sensors exposed to 5 ppm NH_3_ at room temperature.

All gas sensing materials mentioned above had been constructed onto the conventional Au interdigital electrode (AuE) type gas sensors to form the AuE-N-C, AuE-Ni NPs/N-C, AuE-Ni-N-C, AuE-Ti_3_C_2_T_x_, and AuE-Ni-N-C/Ti_3_C_2_T_x_ gas sensors for all the subsequent testing at room temperature. Although the conductivity of the AuE-Ti_3_C_2_T_x_ device was higher than that of the four sensors (**Fig. S20a**), it was to be noted that the NH_3_ gas sensing curve of the AuE-Ti_3_C_2_T_x_ sensor cannot be restored (**Fig. S20b**), which was mainly due to the large stacking density of synthetic Ti_3_C_2_T_x_. In addition, the slope of the *I-V* curve for the AuE-Ni-N-C/Ti_3_C_2_T_x_ sensor was enhanced in comparison with that of the AuE-Ni-N-C and AuE-Ni NPs/N-C sensors, which indicated that the conductivity of the Ni-N-C/Ti_3_C_2_T_x_ hybrid film was higher than that of the two sensors. Furthermore, the AuE-Ni NPs/N-C sensor was dominated by different sensing mechanisms (positive response in **Fig. S20d**) compared to the AuE-Ni-N-C and AuE-Ni-N-C/Ti_3_C_2_T_x_ sensors. The recovery rate of N-C, Ni NPs/N-C, Ni-N-C, and Ni-N-C/Ti_3_C_2_T_x_ sensitive materials was evaluated by fixing the response time of all sensors to 30 s. Although the surface gas diffusion rate and surface reaction rate of all sensitive materials did not reach equilibrium within 30 s, the change in the slope of all response curves indicates that the selected 30-second response time is appropriate. The recovery time of the AuE-Ni NPs/N-C sensor (40 s, **Fig. S20d**) to NH_3_ is shorter than that of the AuE-N-C sensor (50 s, **Fig. S20c**), indicating that the number of surface-active sites affect the desorption rate of NH_3_. Furthermore, compared to the AuE-Ni-N-C gas sensor (125 s, **Fig. S20e**), the AuE-Ni-N-C/Ti_3_C_2_T_x_ sensor exhibits a shorter recovery time for 5 ppm NH_3_ (115 s, **Fig. S20f**). This improvement can be attributed to the high charge transfer efficiency achieved by the Ti_3_C_2_T_x_ conductive network, thereby decreasing the recovery time of the sensor to NH_3_.


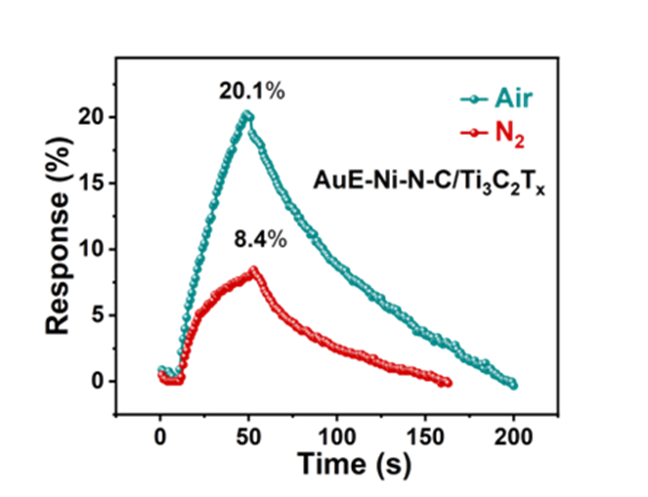


**Fig. S21** The response curves of AuE-Ni-N-C/Ti_3_C_2_T_x_ sensor to 5 ppm NH_3_ in the air and N_2_.


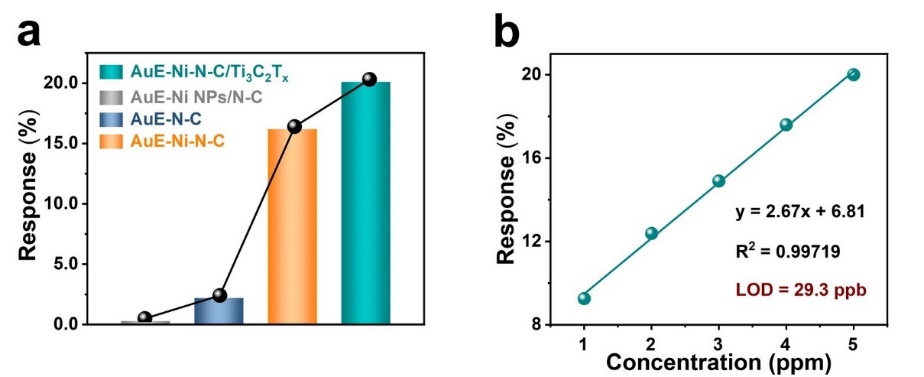


**Fig. S22 Performance of different gas sensors for low-concentration NH_3_ detection.** **a** Comparison of the response of the AuE-N-C, AuE-Ni-N-C, AuE-Ni NPs/N-C, and AuE-Ni-N-C/Ti_3_C_2_T_x_ sensors to 5 ppm NH_3_ at room temperature. **b** Response curves versus NH_3_ concentration for AuE-Ni-N-C/Ti_3_C_2_T_x_ sensor.

The response of low concentration NH_3_ (from 1 to 5 ppm) of the AuE-N-C, AuE-Ni-N-C, and AuE-Ni-N-C/Ti_3_C_2_T_x_ sensors was almost linear (**Figs. S22b, S23c, and S23f**).


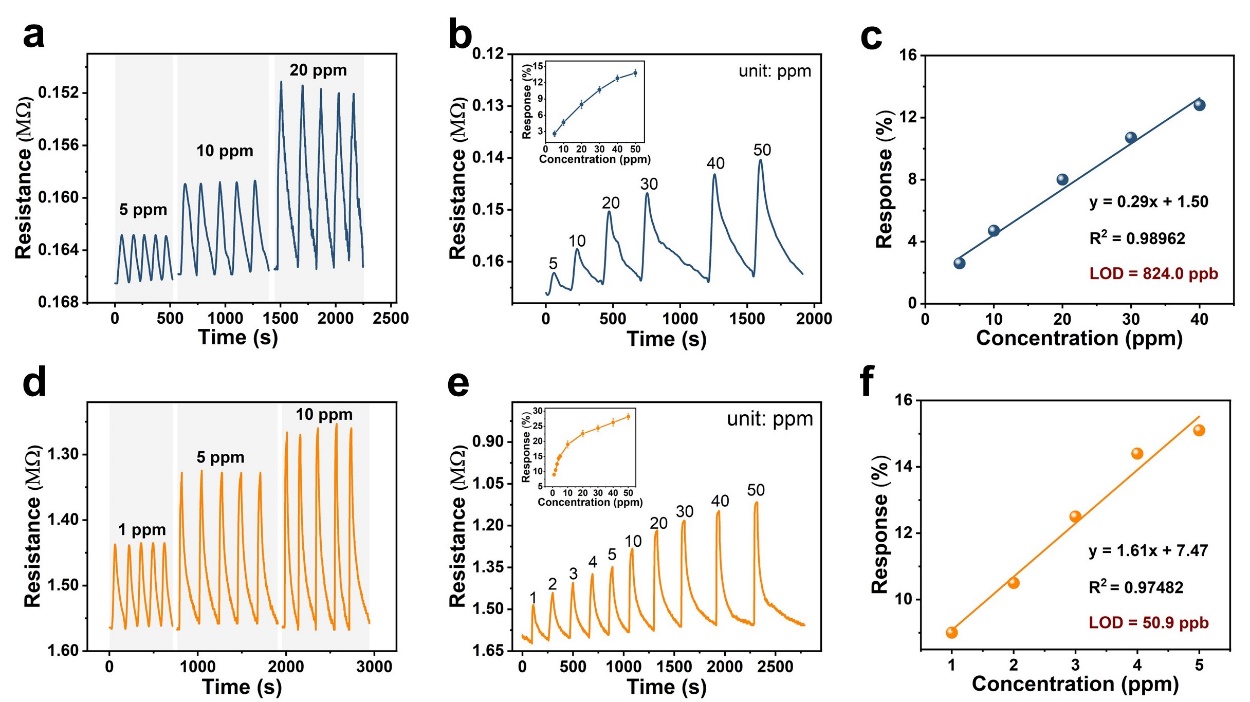


**Fig. S23 Performance of AuE-N-C and AuE-Ni-N-C gas sensors for low-concentration NH_3_ detection.** **a** Repeatability of AuE-N-C sensor toward 1, 5, and 10 ppm NH_3_. **b** The experimental real-time gas response curve of AuE-N-C sensor at different NH_3_ concentrations. The inset is the responses as a function of the gas concentration to different NH_3_ concentrations from 1 to 50 ppm at room temperature for AuE-N-C sensor. **c** Response curves versus NH_3_ concentration for AuE-N-C sensor. **d** Repeatability of AuE-Ni-N-C sensor toward 1, 5, and 10 ppm NH_3_. **e** The experimental real-time gas response curve of AuE-Ni-N-C sensor at different NH_3_ concentrations. The inset is the responses as a function of the gas concentration to different NH_3_ concentrations from 1 to 50 ppm at room temperature for AuE-Ni-N-C sensor. **f** Response curves versus NH_3_ concentration for AuE-Ni-N-C sensor.

The repeatability of the AuE-N-C and AuE-Ni-N-C gas sensors to 1 ppm, 5 ppm, and 10 ppm NH_3_ for five consecutive cycles demonstrated excellent repeatability (**Fig. S23a and d**). Upon cyclic exposure to NH_3_ ranging from 1 to 50 ppm, the time-related dynamic response of the three gas sensors exhibited a stable response and recovery features in **Fig. S23b and e**. Notably, standard deviations of the measured response values for the AuE-N-C, AuE-Ni-N-C, and AuE-Ni-N-C/Ti_3_C_2_T_x_ gas sensors for the various NH_3_ concentrations were only 1.8% at most, suggesting that N-C, Ni-N-C, and Ni-N-C/Ti_3_C_2_T_x_ materials provided favorable stability of gas sensing (**Fig. S23b and e**).


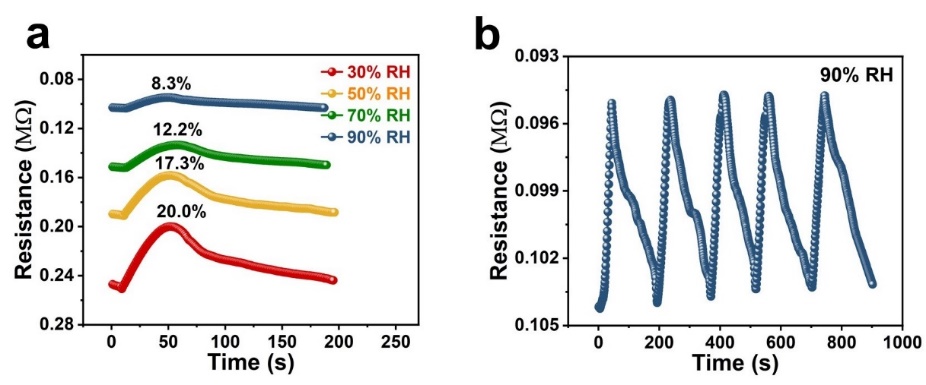


**Fig. S24 Relative humidity analysis of AuE-Ni-N-C/Ti_3_C_2_T_x_ gas sensor for low-concentration NH_3_ detection.** **a** The resistance curve of AuE-Ni-N-C/Ti_3_C_2_T_x_ sensor to 5 ppm NH_3_ under different RH. **b** Repeatability of AuE-Ni-N-C/Ti_3_C_2_T_x_ sensor toward 5 ppm NH_3_ under 90% RH.

Prior to the introduction of NH_3_ gas, the AuE-Ni-N-C/Ti_3_C_2_T_x_ sensor was exposed to moist air with specified RH until a steady-state baseline arrived, then 5 ppm NH_3_ mixed with the specific wet air was purged into the test chamber.


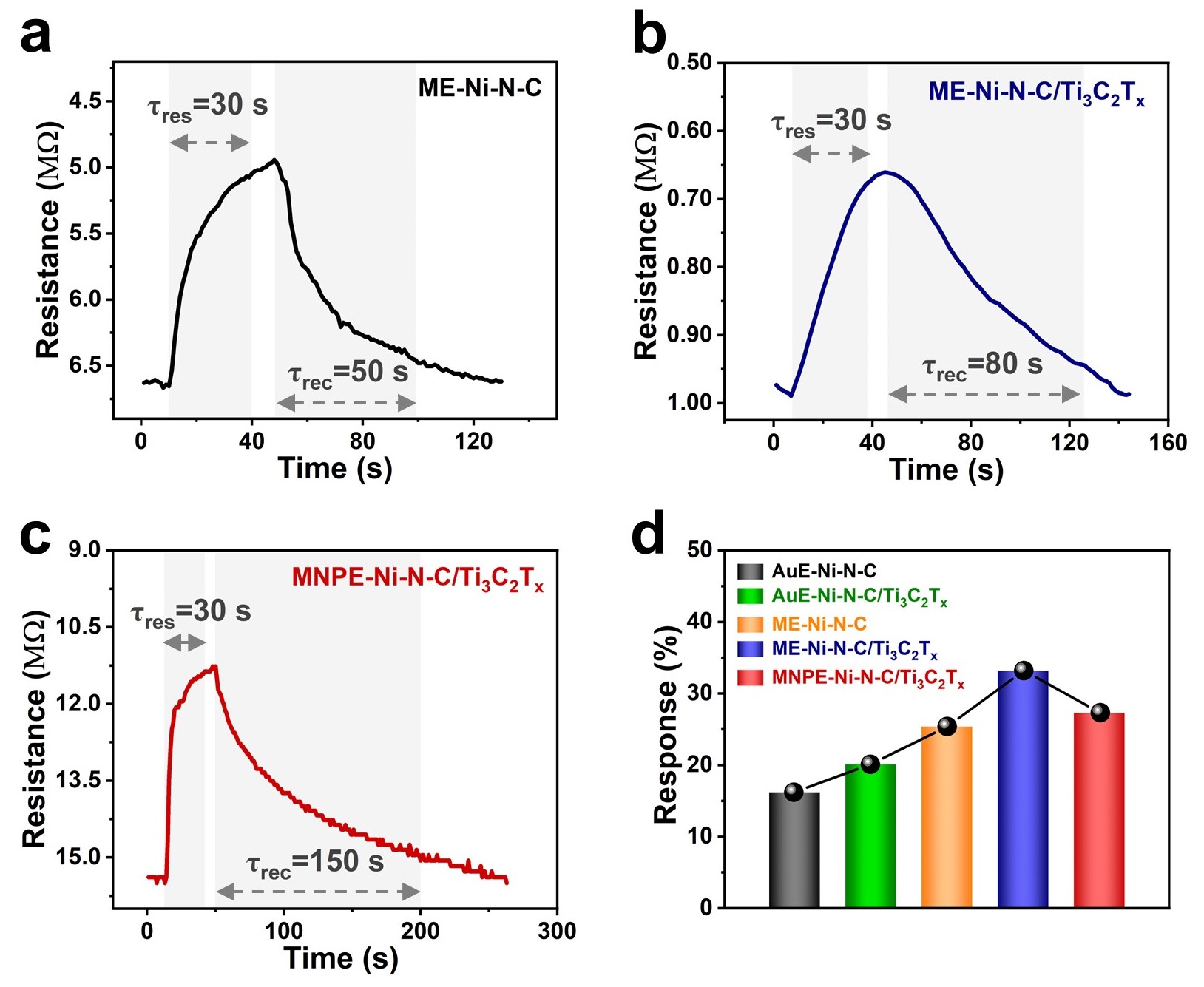


**Fig. S25 Performance of different gas sensors for low-concentration NH_3_ detection.** **a** ME-Ni-N-C, **b** ME-Ni-N-C/Ti_3_C_2_T_x_, and **c** MNPE-Ni-N-C/Ti_3_C_2_T_x_ sensors exposed to 5 ppm NH_3_ at room temperature. **d** Comparison of the response of the AuE-Ni-N-C, AuE-Ni-N-C/Ti_3_C_2_T_x_, ME-Ni-N-C, ME-Ni-N-C/Ti_3_C_2_T_x_, and MNPE-Ni-N-C/Ti_3_C_2_T_x_ sensors to 5 ppm NH_3_ at room temperature.

Furthermore, the recovery time of the ME-Ni-N-C/Ti_3_C_2_T_x_ sensor (80 s, **Fig. S25b**) to NH_3_ is shorter than that of the MNPE-Ni-N-C/Ti_3_C_2_T_x_ sensor (150 s, **Fig. S25c**). This difference is due to the higher conductivity of ME, which accelerates the desorption rate of NH_3_ from the surface of the sensors.


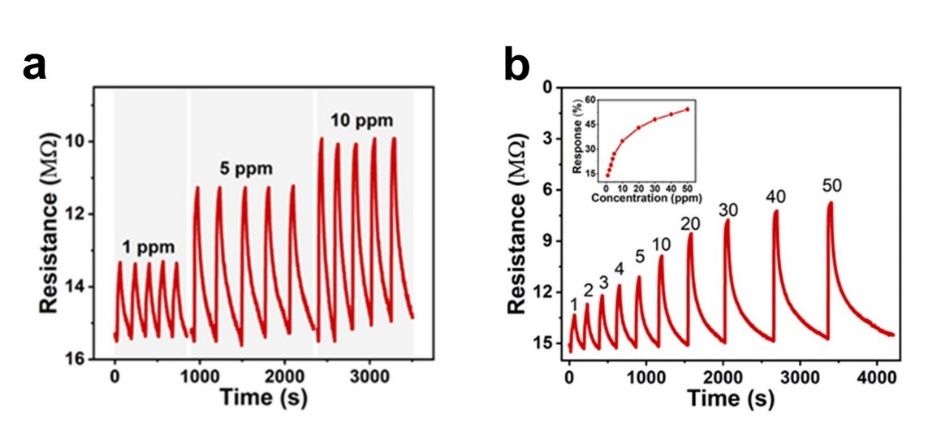


**Fig. S26 Performance of MNPE-Ni-N-C/Ti_3_C_2_T_x_ gas sensor for low-concentration NH_3_ detection.** **a** Repeatability of MNPE-Ni-N-C/Ti_3_C_2_T_x_ sensor toward 1, 5, and 10 ppm NH_3_. **b** The experimental real-time gas response curve of MNPE-Ni-N-C/Ti_3_C_2_T_x_ sensor at different NH_3_ concentrations. The inset is the responses as a function of the gas concentration to different NH_3_ concentrations from 1 to 50 ppm at room temperature for the MNPE-Ni-N-C/Ti_3_C_2_T_x_ sensor.

The exposure of the MNPE-Ni-N-C/Ti_3_C_2_T_x_, ME-Ni-N-C/Ti_3_C_2_T_x,_ and ME-Ni-N-C sensors to a range of ppm NH_3_ for several cycles demonstrated excellent reproductivity without major changes (**Figs. S26a, S27a, and S27d**). The response/recovery curves of the MNPE-Ni-N-C/Ti_3_C_2_T_x_, ME-Ni-N-C/Ti_3_C_2_T_x_ and ME-Ni-N-C sensors to different concentrations of NH_3_ from 1 to 50 ppm at room temperature were described in **Figs. S26b, S27b, and S27e**. It was worth noting that under the condition of ensuring that the response time was 40 s, the recovery performance of the ME-Ni-N-C/Ti_3_C_2_T_x_ sensor for different concentrations of NH_3_ was better than that of the MNPE-Ni-N-C/Ti_3_C_2_T_x_ sensor, in line with the recovery time analyze in **Fig. 4c**.


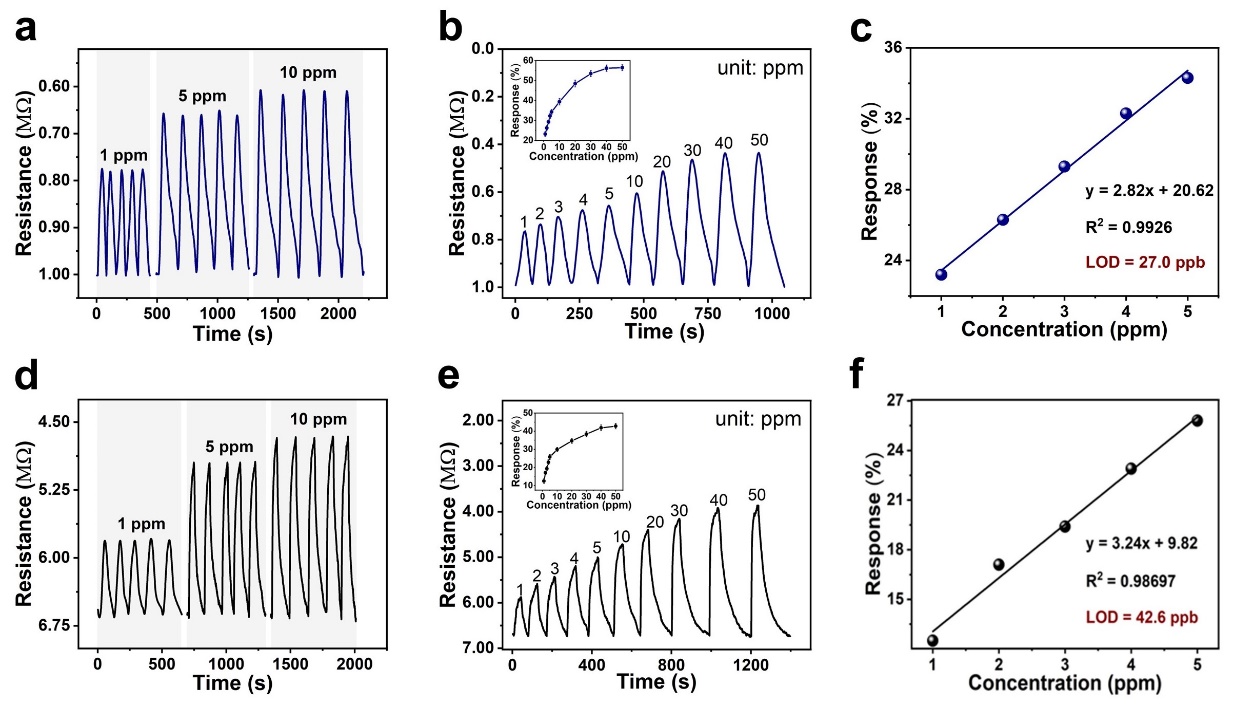


**Fig. S27 Performance of ME-Ni-N-C/Ti_3_C_2_T_x_ and ME-Ni-N-C gas sensors for low-concentration NH_3_ detection.** **a** Repeatability of ME-Ni-N-C/Ti_3_C_2_T_x_ sensor toward 1, 5, and 10 ppm NH_3_. **b** The experimental real-time gas response curve of ME-Ni-N-C/Ti_3_C_2_T_x_ sensor at different NH_3_ concentrations. The inset is the responses as a function of the gas concentration to different NH_3_ concentrations from 1 to 50 ppm at room temperature for ME-Ni-N-C/Ti_3_C_2_T_x_ sensor. **c** Response curves versus NH_3_ concentration for ME-Ni-N-C/Ti_3_C_2_T_x_ sensor. **d** Repeatability of ME-Ni-N-C sensor toward 1, 5, and 10 ppm NH_3_. **e** The experimental real-time gas response curve of ME-Ni-N-C sensor at different NH_3_ concentrations. The inset is the responses as a function of the gas concentration to different NH_3_ concentrations from 1 to 50 ppm at room temperature for ME-Ni-N-C sensor. **f** Response curves versus NH_3_ concentration for ME-Ni-N-C sensor.

In addition, the theoretical LOD of the ME-Ni-N-C sensor for NH_3_ was calculated to be 42.6 ppb (**Fig. S27f**).


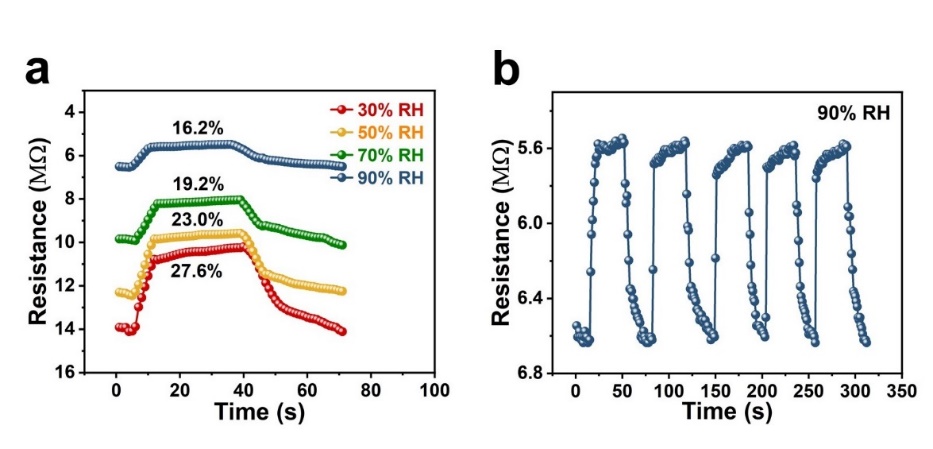


**Fig. S28 Relative humidity analysis of MNPE-Ni-N-C/Ti_3_C_2_T_x_ gas sensor for low-concentration NH_3_ detection.** **a** The resistance curve of MNPE-Ni-N-C/Ti_3_C_2_T_x_ sensor to 5 ppm NH_3_ under different humidity. **b** Repeatability of MNPE-Ni-N-C/Ti_3_C_2_T_x_ sensor toward 5 ppm NH_3_ under 90% RH.

Interestingly, replacing metal electrodes with non-metallic electrodes also reduced the SBH between electrodes and semiconductors, significantly improving the recovery performance of the MNPE-Ni-N-C/Ti_3_C_2_T_x_ and ME-Ni-N-C/Ti_3_C_2_T_x_ sensors (less than 55 s in **Figs. S28a and S29a**).


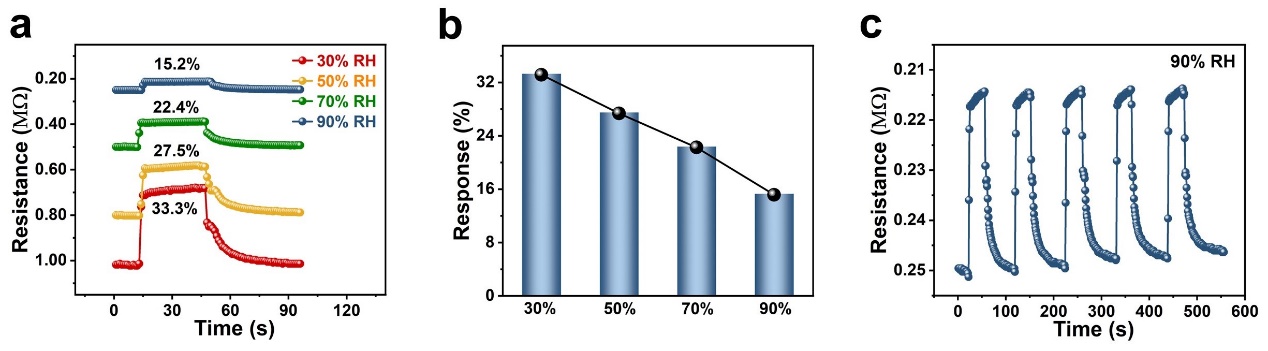


**Fig. S29 Relative humidity analysis of ME-Ni-N-C/Ti_3_C_2_T_x_ gas sensor for low-concentration NH_3_ detection.** **a** The resistance curve of ME-Ni-N-C/Ti_3_C_2_T_x_ sensor to 5 ppm NH_3_ under different RH. **b** Comparison of the response of the ME-Ni-N-C/Ti_3_C_2_T_x_ sensor to 5 ppm NH_3_ under different RH at room temperature. **c** Repeatability of ME-Ni-N-C/Ti_3_C_2_T_x_ sensor toward 5 ppm NH_3_ under 90% RH.

In addition, the response of the MNPE-Ni-N-C/Ti_3_C_2_T_x_ sensor decreased with increasing RH (**Fig. S29b**).


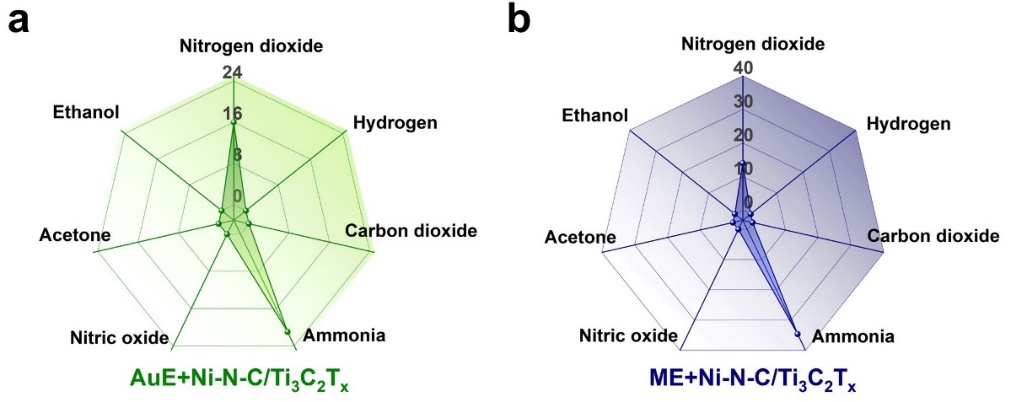


**Fig. S30 Selectivity analysis of AuE-****Ni-N-C/Ti_3_C_2_T_x_ and ME-Ni-N-C/Ti_3_C_2_T_x_ gas sensors.** **a** Selectivity of AuE-Ni-N-C/Ti_3_C_2_T_x_ sensor to different gases (5 ppm NH_3_, 50 ppm NO_2_, CO_2_, NO, H_2_, acetone-saturated steam, and ethanol-saturated steam). **b** Selectivity of ME-Ni-N-C/Ti_3_C_2_T_x_ sensor to different gases.

The MNPE-Ni-N-C/Ti_3_C_2_T_x_ and ME-Ni-N-C/Ti_3_C_2_T_x_ sensors displayed higher sensitivity to NH_3_ compared to the AuE-Ni-N-C/Ti_3_C_2_T_x_ sensor, due to the higher adsorption energy of the flexible MXene-based electrodes for NH_3_ and the match of work function between the metal electrode and semiconductor sensing materials which allowed a fluent transfer of more charge carriers (**Fig. 4f and Fig. S30**).


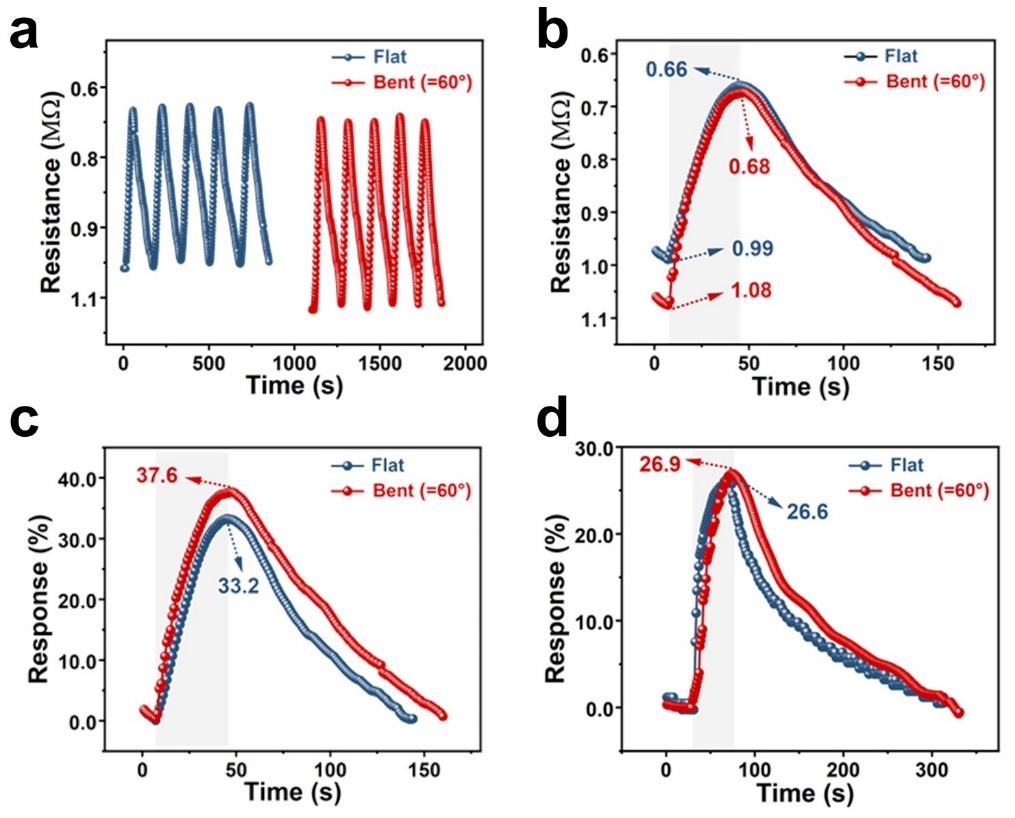


**Fig. S31 Bending stability analysis of ME-Ni-N-C/Ti_3_C_2_T_x_ and MNPE-Ni-N-C/Ti_3_C_2_T_x_ gas sensors. a** Repeatability of ME-Ni-N-C/Ti_3_C_2_T_x_ sensor toward 5 ppm NH_3_ before and after bending 500 times. **b** Resistance and **c** response curves of the ME-Ni-N-C/Ti_3_C_2_T_x_ flexible sensor to 5 ppm NH_3_ when tested under 60° bending angles. **d** Response curves of the MNPE-Ni-N-C/Ti_3_C_2_T_x_ flexible sensor to 5 ppm NH_3_ when tested under 60° bending angles.

In addition, the increase in the response of the ME-Ni-N-C/Ti_3_C_2_T_x_ sensor may be attributed to the formation of more micro cracks and defects at the edge positions of the flexible ME and Ni-N-C/Ti_3_C_2_T_x_ hetero-nanojunction after bending, which can result in increased gas adsorption (**Fig. S31c**).


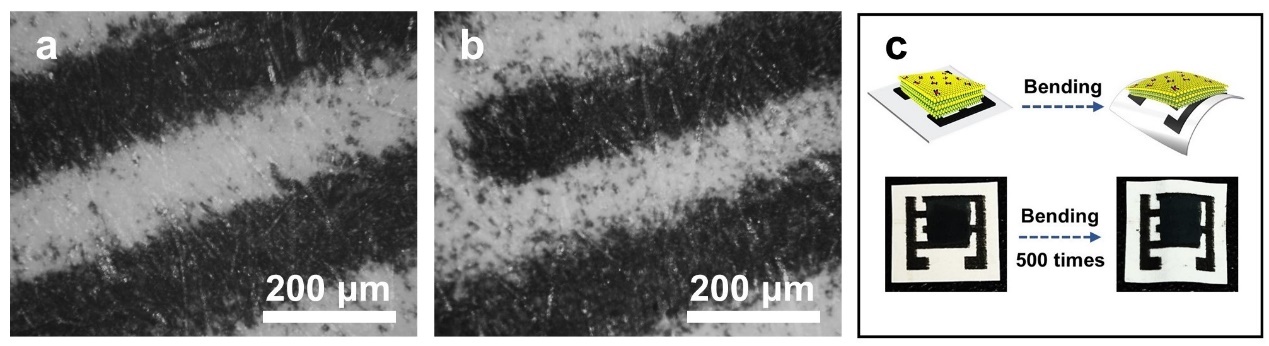


**Fig. S32 Analysis of adhesion between MNPE-Ni-N-C/Ti_3_C_2_T_x_ sensor and paper before and after bending. a** The optical image of MNPE before 500 cycles of 60° bending. **b** The optical image of MNPE after 500 cycles of 60° bending. **c** The optical photos of the MNPE-Ni-N-C/Ti_3_C_2_T_x_ sensor before and after bending (500 times).


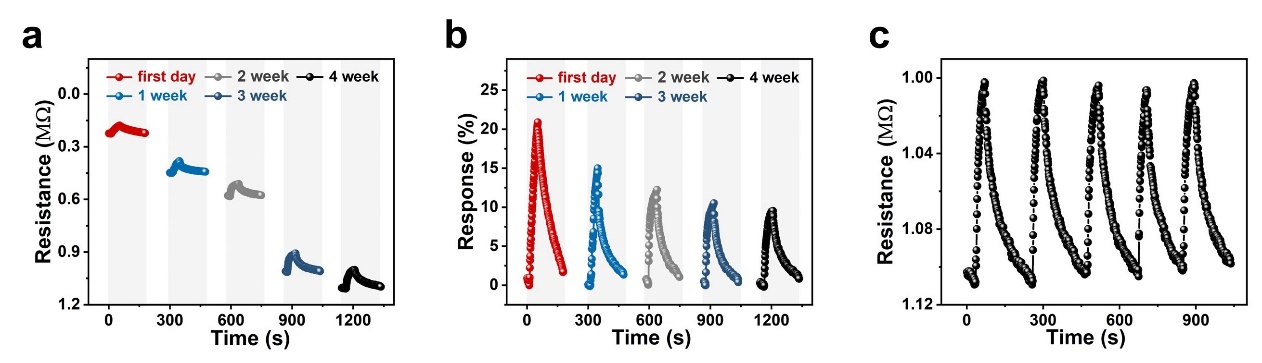


**Fig. S33 Long-term stability analysis of AuE-Ni-N-C/Ti_3_C_2_T_x_ gas sensors.** **a** The resistance and **b** response curves of AuE-Ni-N-C/Ti_3_C_2_T_x_ sensor to 5 ppm NH_3_ at different weeks. **c** Repeatability of AuE-Ni-N-C/Ti_3_C_2_T_x_ sensor toward 5 ppm NH_3_ after 4 weeks.

The repeatability of the two sensors after five consecutive cycles of exposure to 5 ppm NH_3_ gas after four weeks confirmed their recoverability (**Figs. S33c and S34c**).


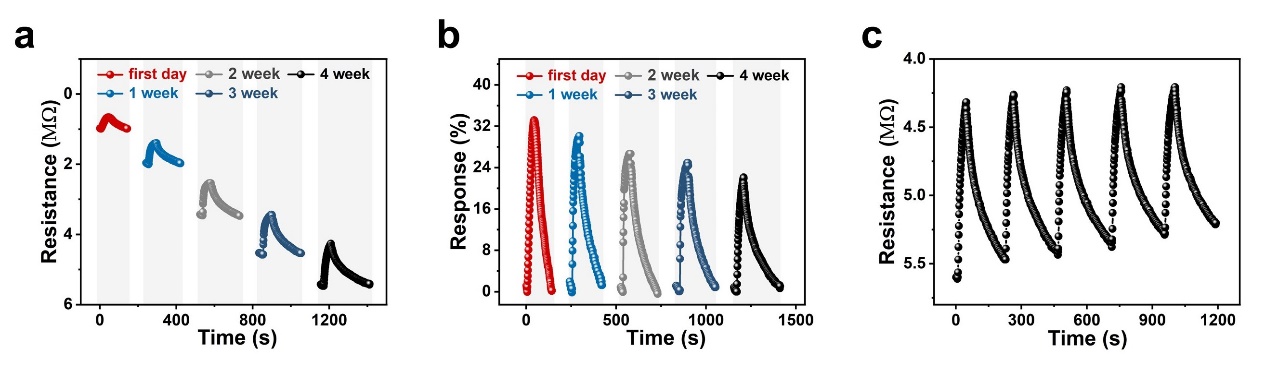


**Fig. S34 Long-term stability analysis of ME-Ni-N-C/Ti_3_C_2_T_x_ gas sensors.** **a** The resistance and **b** response curves of ME-Ni-N-C/Ti_3_C_2_T_x_ sensor to 5 ppm NH_3_ at different weeks. **c** Repeatability of ME-Ni-N-C/Ti_3_C_2_T_x_ sensor toward 5 ppm NH_3_ after 4 weeks.


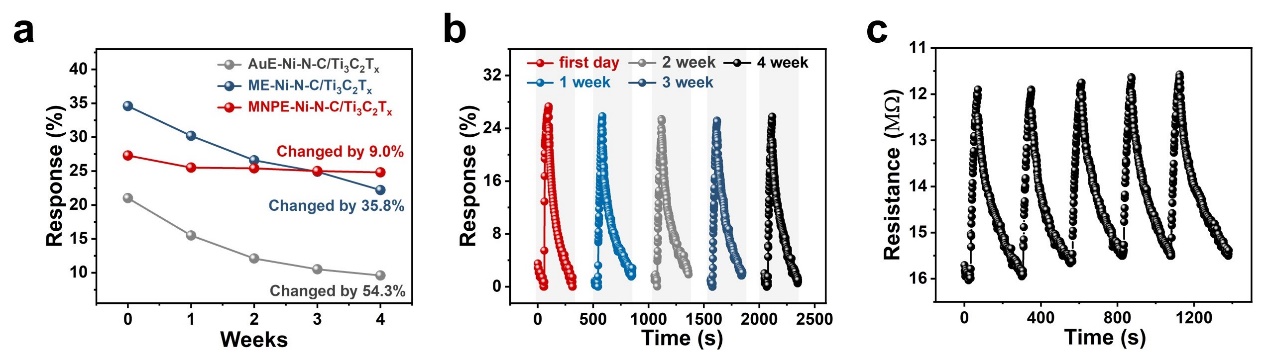


**Fig. S35 Long-term stability analysis of MNPE-Ni-N-C/Ti_3_C_2_T_x_ gas sensors.** **a** The changes in response of AuE-Ni-N-C/Ti_3_C_2_T_x_, ME-Ni-N-C/Ti_3_C_2_T_x_, and MNPE-Ni-N-C/Ti_3_C_2_T_x_ sensors to 5 ppm NH_3_ within 4 weeks. **b** The response curve of MNPE-Ni-N-C/Ti_3_C_2_T_x_ sensor to 5 ppm NH_3_ at different weeks. **c** Repeatability of MNPE-Ni-N-C/Ti_3_C_2_T_x_ sensor toward 5 ppm NH_3_ after 4 weeks.


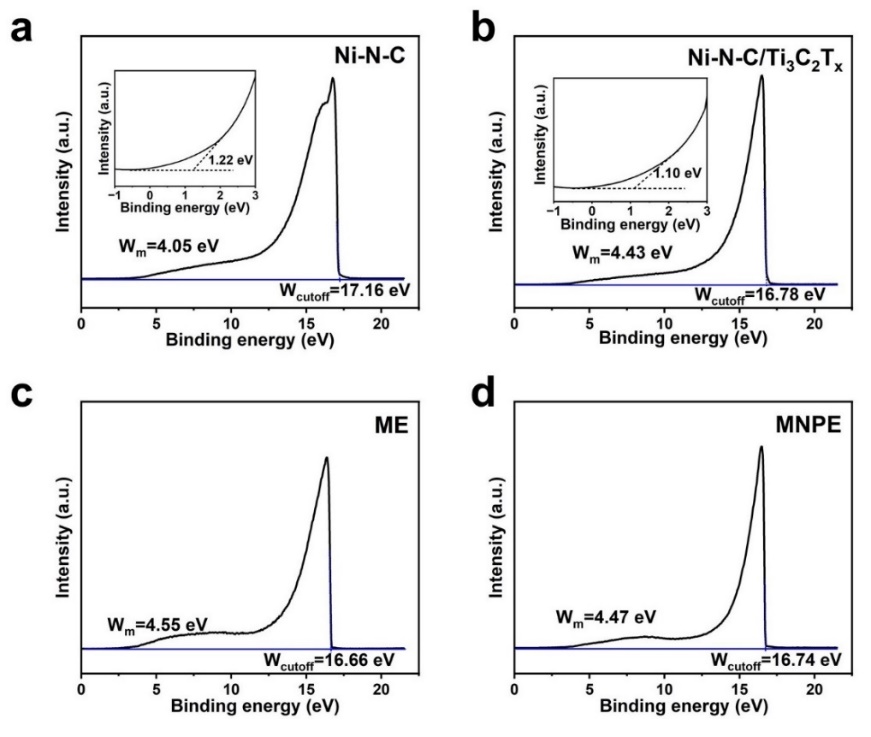


**Fig. S36 Work function analysis of Ni-N-C, Ni-N-C/Ti_3_C_2_T_x_, ME, and MNPE.** UPS spectra of **a** Ni-N-C, **b** Ni-N-C/Ti_3_C_2_T_x_, **c** ME, and **d** MNPE.

The work functions of ME and MNPE materials were demonstrated in **Fig. S36c and d**, with values of 4.55 and 4.47 eV, respectively. The use of a Ti_3_C_2_T_x_-MXene non-metallic electrode in combination with Ni-N-C/Ti_3_C_2_T_x_ had positive effects on gas sensing performance compared to the AuE-Ni-N-C/Ti_3_C_2_T_x_ sensor. When the Ni-N-C/Ti_3_C_2_T_x_ gas sensing material (E_c_ = −4.16 eV, E_v_ = −5.53 eV, and work function is 4.43 eV) was in contact with an Au electrode (with a work function of 5.10 eV), the SBH hindered the efficient transport of charge carriers, resulting in only a small percentage of carriers having sufficient energy to cross the Schottky junction.


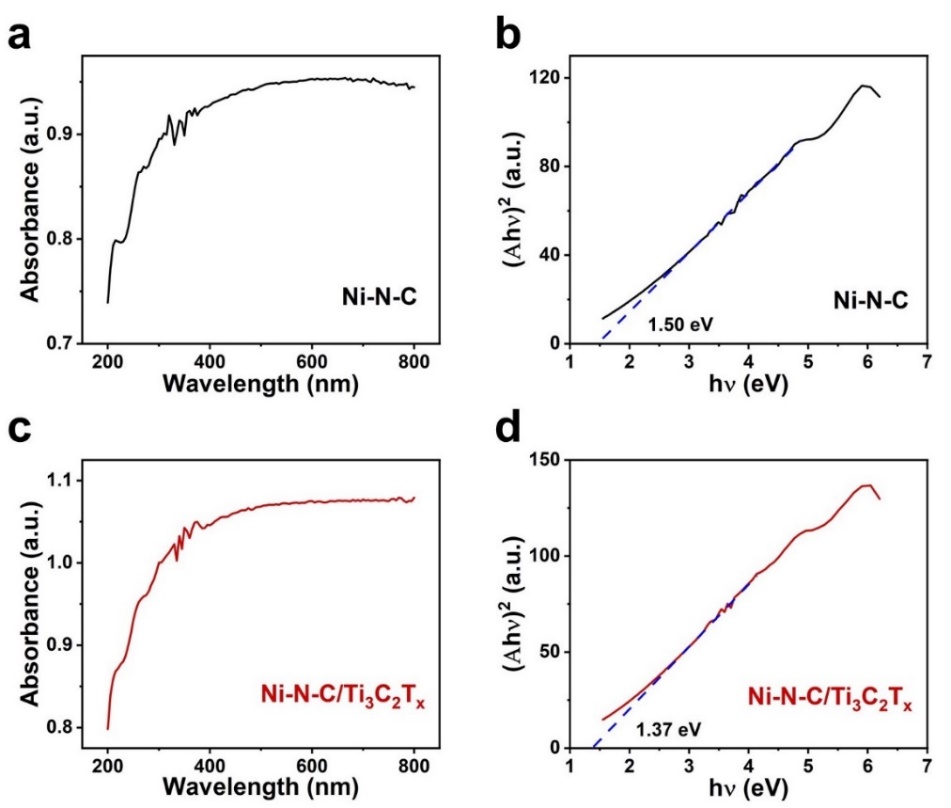


**Fig. S37 Band gap analysis of Ni-N-C and Ni-N-C/Ti_3_C_2_T_x_ materials.** **a** UV-vis spectra of Ni-N-C. **b** plot of (Ahν)^2^ against (hν) of Ni-N-C. **c** UV-vis spectra of Ni-N-C/Ti_3_C_2_T_x_. **d** plot of (Ahν)^2^ against (hν) of Ni-N-C/Ti_3_C_2_T_x_.


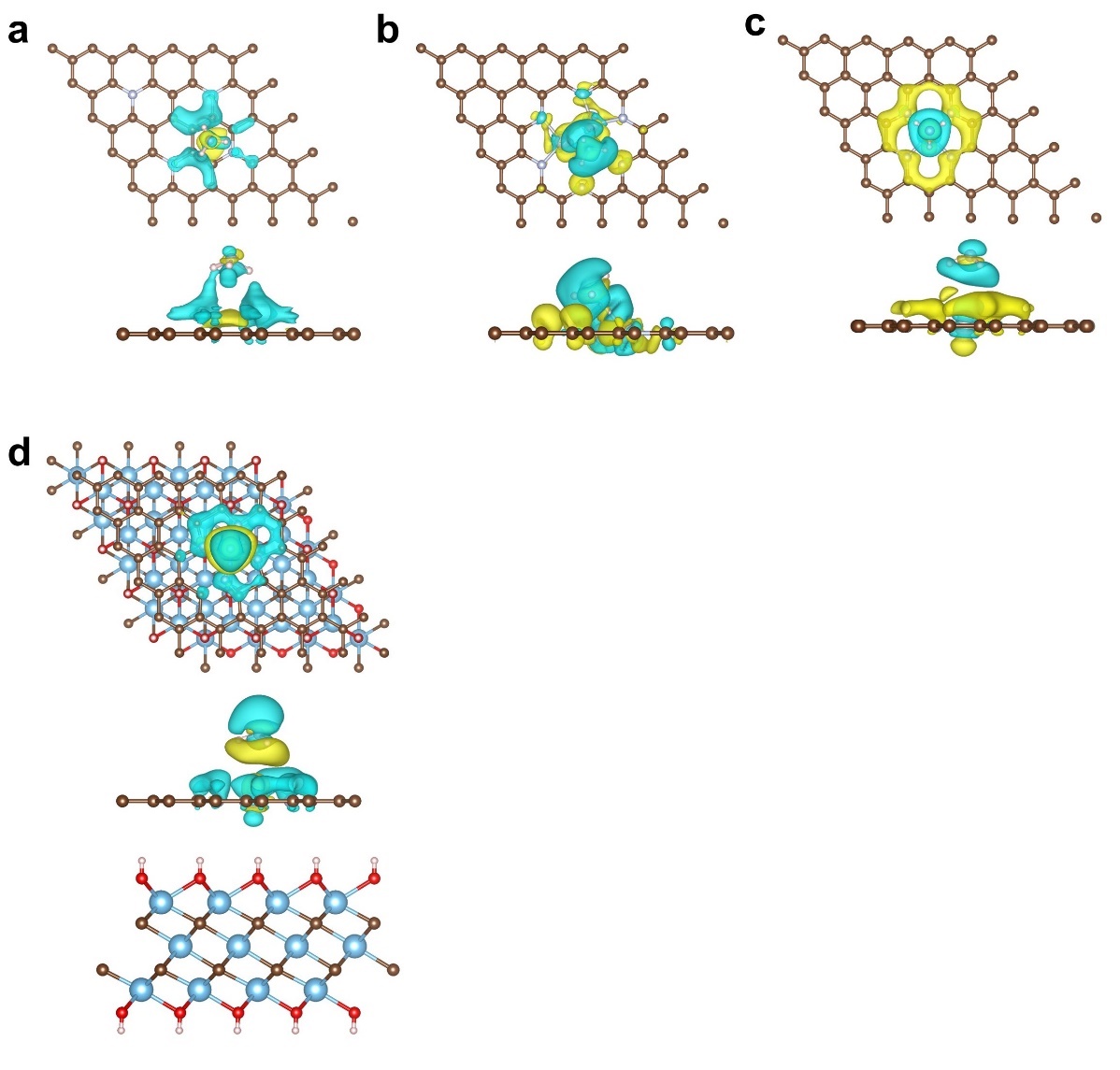


**Fig. S38 Gas adsorption analysis of N-C, Ni NPs/N-C, Ni-N-C, and Ni-N-C/Ti_3_C_2_T_x_ materials.** Top and side views of the isosurface charge density plots of NH_3_ adsorbed on the **a** N-C, **b** Ni NPs/N-C, **c** Ni-N-C, and **d** Ni-N-C/Ti_3_C_2_T_x_, respectively, using an isovalue of 0.0005 e Å^−3^ for NH_3_. Cyan: charge depletion. Yellow: charge accumulation.


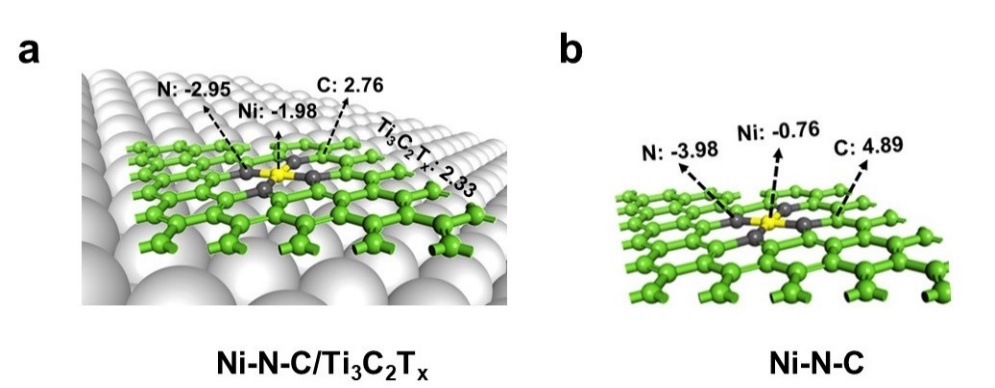


**Fig. S39 Electron transfer analysis of Ni-N-C and Ni-N-C/Ti_3_C_2_T_x_ materials.** Bader charge and charge density difference of **a** Ni-N-C/Ti_3_C_2_T_x_ and **b** Ni-N-C.


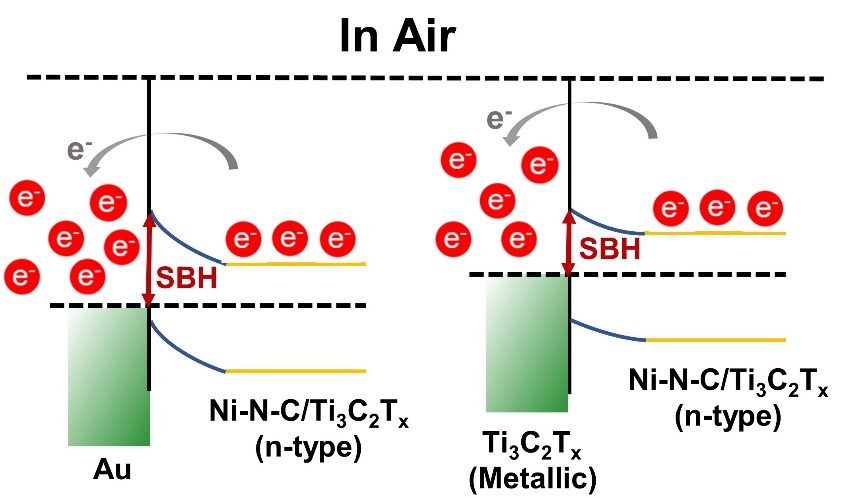


**Fig. S40 SBH analysis of Au-electrode-Ni-N-C/Ti_3_C_2_T_x_ and Ti_3_C_2_T_x_-based electrode-Ni-N-C/Ti_3_C_2_T_x_.** Schematic diagrams of band structure for Au-electrode-Ni-N-C/Ti_3_C_2_T_x_ and Ti_3_C_2_T_x_-based electrode-Ni-N-C/Ti_3_C_2_T_x_ in the air at room temperature.**Table S1.** Atomic contents (at.%) determined by ICP-OES for Ni-N-C and Ni-N-C/Ti_3_C_2_T_x_ catalysts.

| Catalysts | Ni (at.%) |
| --- | --- |
| Ni-N-C | 0.10 |
| Ni-N-C/Ti_3_C_2_T_x_ | 0.25 |

**Table S2.** BET surface areas (S_BET_) and pore distribution (V_total_) of the precursors and corresponding calcination catalysts.

| Samples | S_BET_ (m^2^/g) | V_total_ (cm^3^/g) |
| --- | --- | --- |
| ZIF-8 | 998.62 | 0.62 |
| Ni-ZIF-8 | 943.18 | 0.53 |
| Ni-ZIF-8-CTAB-Ti_3_C_2_T_x_ | 381.16 | 0.22 |
| N-C | 695.79 | 0.82 |
| Ni NPs/N-C | 180.63 | 0.15 |
| Ni-N-C | 352.41 | 0.33 |
| Ni-N-C/Ti_3_C_2_T_x_ | 535.10 | 0.52 |

**Table S3.** Structural parameters extracted from the EXAFS fitting of Ni-N-C and Ni-N-C/Ti_3_C_2_T_x_.

CN, coordination numbers; *R*, bond distance; σ^2^, Debye-Waller factors; ΔE_0_, the inner potential correction. *R* factor, the goodness of fit. *Ѕ*_0_^2^ was set to 0.80, according to the experimental EXAFS fit of Ni foil reference by fixing CN as the known crystallographic value.

| Sample | shell | CN | R(Å) | σ^2^ | ΔE_0_ | R factor |
| --- | --- | --- | --- | --- | --- | --- |
| Ni foil | Ni-Ni | 12 | 2.48±0.01 | 0.0060 | −6.1±0.6 | 0.0045 |
| Ni-N-C | Ni-N | 3.4±0.1 | 1.86±0.01 | 0.0069 | −7.9±1.1 | 0.0030 |
|  | Ni-Ni | 0.8±0.1 | 2.53±0.01 | 0.0069 |  |  |
|  | Ni-Ni1 | 1.4±0.4 | 2.77±0.02 | 0.0162 |  |  |
| Ni-N-C/Ti_3_C_2_T_x_ | Ni-N | 3.8±0.2 | 1.85±0.01 | 0.0073 | −7.6±1.8 | 0.0122 |
|  | Ni-Ni | 0.4±0.1 | 2.80±0.03 | 0.0062 |  |  |

**Table S4.** Fluidic properties of ME and MNPE inks.

| Sample | Density  (g/cm^3^) | Surface tension  (mN/m) | Viscosity  (cP) | *Z* |
| --- | --- | --- | --- | --- |
| ME | 0.951 | 56.90 | 7.04 | 10.44 |
| MNPE | 0.964 | 15.26 | 9.17 | 4.18 |

Note: The viscosity was evaluated at the sheer rate of 5 s^−1^

The following Equation is used to calculate the *Z* value to describe the printability [S10]:

$$z=\frac{1}{Oh}=\frac{\sqrt{\gamma\alpha\rho}}{\eta}$$

Where *γ* is the surface tension, *ρ* is the density of the fluid, α is the inkjet nozzle diameter, and *η* is the dynamic viscosity. Generally, the *Z* value of inks suitable for inkjet printing is regarded as between 1 and 14 [S11]. The *Z* values of ME and MNPE inks are 10.44 and 4.18, respectively, suggesting that both two inks can spray stably in the printing process.

**Table S5.** Resolution comparison of reported functional ink printing.

| Materials | Solvent | Method | σ, S cm^−1^ | Substrate | Post-processing | Ref. |
| --- | --- | --- | --- | --- | --- | --- |
| Zn microparticles | PVP | Screen printing | 3.0×10^3^ | PLGA | Electrochemical sintering | [S12] |
| Ti_3_C_2_T_x_ | Water | Extrusion printing | 6.90×10^3^ | PET | - | [S13] |
| AgInGa-SIS | Toluene | Extrusion printing | 7.02×10^3^ | SIS | 60 °C/60 mins | [S14] |
| 56 wt% silver nanoflakes | Water | Stencil printing | 7.38×10^3^ | PDMS | 80 °C/30 mins | [S15] |
| 80 wt% silver nanoflakes | PDMS | Stencil printing | 1.51×10^4^ | Silicone | 160 °C/60 mins | [S16] |
| Ti_3_C_2_T_x_/TFA | Ethanol | electrohydrodynamic (EHD) printing | 8.90×10^3^ | SiO_2_/Si | 120 °C/60 mins | [S17] |
| Ti_3_C_2_T_x_ | NMP | Inkjet printing | 2.75×10^2^ | PET | - | [S18] |
| Ti_3_C_2_T_x_ | NMP | Inkjet printing | 2.77×10^3^ | AlO_x_-coated PET | 60 °C | [S19] |
| Ti_3_C_2_T_x_ | DMSO | Inkjet printing | 1.08×10^3^ | PET | 70 °C | [S20] |
| Ti_3_C_2_T_x_/siloxane surfactant | Water | 3D printing | 2.45×10^3^ | PDMS | room temperature/72h | [S21] |
| Ti_3_C_2_T_x_ | Water | Inkjet printing | 6.38×10^3^ | Paper | 60 °C/10 mins | This work |
| Ti_3_C_2_T_x_ | PEDOT: PSS/  NMP | Inkjet printing | 3.70×10^3^ | Paper | 60 °C/10 mins | This work |

**Table S6.** FoM data (FoM = σc) of the different inks.

| Samples | σ (S cm^−1^) | c (mg mL^−1^) | FoM (S cm^−1^ mg mL^−1^) | Ref. |
| --- | --- | --- | --- | --- |
| rGO | 15 | 3 | 45 | [S22] |
| GO/PA | 64 | 25 | 1600 | [S23] |
| Graphene | 391 | 2.25 | 879.75 | [S24] |
| Graphene | 240 | 3 | 720 | [S25] |
| Graphene | 250 | 20 | 5000 | [S26] |
| PEDOT:PSS | 700 | 10 | 7000 | [S27] |
| PEDOT:PSS | 22 | 10 | 220 | [S28] |
| MXene | 1080 | 2.25 | 2430 | [S20] |
| p-MXene | 161 | 20 | 3220 | [S29] |
| MXene | 2770 | 36 | 99720 | [S19] |
| ME | 6380 | 14 | 89320 | This work |
| MNPE | 3700 | 16 | 59200 | This work |

**Table S7.** The comparison of sensing performance of NH_3_ gas sensors with different MXene-based sensing materials.

| Materials | Temp. | Response | τ_res._ (s) | τ_rec._ (s) | | LOD (ppb) | Ref. |
| --- | --- | --- | --- | --- | --- | --- | --- |
| Au-Ti_3_C_2_T_x_ MXene | RT | 16%  (100 ppm)^a^ | 87 | 217 | - | | [S30] |
| MXene/rGO | RT | 6.77%  (50 ppm)^b^ | 25 | 25 | - | | [S31] |
| TiO_2_-Ti_3_C_2_T_x_ | RT | 3.1%  (10 ppm)^a^ | 33 | 277 | - | | [S32] |
| Ti_3_C_2_ MXene | RT | 6.13%  (500 ppm)^a^ | 45  (25 ppm) | 94  (25 ppm) | - | | [S33] |
| Ti_3_C_2_T_x_-SnO_2_ | RT | 75%  (500 ppm)^a^ | 109 | 342 | - | | [S34] |
| ME-Ni-N-C/Ti_3_C_2_T_x_ | RT | 33.2%  (5 ppm)^a^ | 30 | 80 | 27.0 | | This work |
| MNPE-Ni-N-C/Ti_3_C_2_T_x_ | RT | 27.3%  (5 ppm)^a^ | 30 | 150 | 12.1 | | This work |

Temp.: Work temperature, RT: Room temperature, *τ_res_*_._: Response time, *τ_rec_*_._: Recovery time, -: Not mention.

^a^ (Δ*R*/*R_a_)*100%*.

^b^ Δ*R*/*R_0_*.

**Table S8.** Comparison of the response to NH_3_ of three sensors under different RH conditions.

| Sensors | Response to NH_3_ in dry air (R_DA_) | Response to NH_3_ at 90%RH (R_90%RH_) | | | (R_DA_-R_90%RH_)/R_DA_ |
| --- | --- | --- | --- | --- | --- |
| AuE-Ni-N-C/Ti_3_C_2_T_x_ | 20.1% | | 8.3% | 58.7% | |
| ME-Ni-N-C/Ti_3_C_2_T_x_ | 33.2% | | 15.2% | 54.2% | |
| MNPE-Ni-N-C/Ti_3_C_2_T_x_ | 27.3% | | 16.2% | 40.7% | |

**Table S9.** The quantification of different oxygen species was determined by analyzing the O 1s XPS spectra for Ni NPs/N-C, Ni-N-C, and Ni-N-C/Ti_3_C_2_T_x_ materials.

| Materials | O_2_^−^  (% Area) | O_2_^−^  Position | O^2−^  (% Area) | O^2−^  Position | O_2_^−^/O^2−^ |
| --- | --- | --- | --- | --- | --- |
| Ni NPs/N-C | 27.3 | 533.6 eV | 72.7 | 531.9 eV | 0.38 |
| Ni-N-C | 37.2 | 533.4 eV | 62.8 | 532.0 eV | 0.59 |
| Ni-N-C/Ti_3_C_2_T_x_ | 72.8 | 532.6 eV | 27.2 | 531.0 eV | 2.67 |

**Table S10.** The surface adsorption energies and charge transfer on N-C (001), Ni NPs/N-C (001), Ni-N-C (001), and Ni-N-C (001)/Ti_3_C_2_T_x_ (001).

| Substrate | E_ad._ (eV) | Charge transfer (e) |
| --- | --- | --- |
| N-C (001) | −0.59 | −0.04 |
| Ni NPs/N-C (001) | −0.70 | −0.11 |
| Ni-N-C (001) | −0.73 | −0.15 |
| Ni-N-C (001)/Ti_3_C_2_T_x_ (001) | −0.76 | −0.16 |

**Table S11.** The surface adsorption energies and charge transfer of NH_3_, NO_2_, NO, acetone, ethanol, and H_2_ on Ni-N-C (001)/Ti_3_C_2_T_x_ (001).

| gas | E_ad._ (eV) | Charge transfer (e) |
| --- | --- | --- |
| NH_3_ | −0.76 | −0.16 |
| NO_2_ | −0.66 | 0.12 |
| NO | −0.22 | 0.06 |
| Acetone | −0.19 | −0.05 |
| Ethanol | −0.18 | −0.05 |
| H_2_ | −0.11 | −0.03 |

**Supplementary References**

[S1] C.M. Zhao, X.Y. Dai, T. Yao, W. X. Chen, X.Q. Wang et al., Ionic exchange of metal organic frameworks to access single nickel sites for efficient electroreduction of CO_2_. J. Am. Chem. Soc. **139**, 8078–8081 (2017).

<https://doi.org/10.1021/jacs.7b02736>

[S2] C.C. Cai, Y. A. Chen, P. Hu, T. Zhu, X.Y. Li et al., Regulating the interlayer spacings of hard carbon nanofibers enables enhanced pore filling sodium storage. Small **18**(6), 2105303 (2022).

<https://doi.org/10.1002/smll.202105303>

[S3] X. Wang, H. Li, H. Li, S. Lin, W. Ding et al., 2D/2D 1T-MoS_2_/Ti_3_C_2_ MXene heterostructure with excellent supercapacitor performance. Adv. Funct. Mater. **30**(15), 0190302 (2020).

<https://doi.org/10.1002/adfm.201910302>

[S4] Z.W. Guo, W. Li, W.X. Li, X.J. Hou, S. Luan et al., Hierarchically porous zeolitic imidazolate framework-8 with tunable mesostructure as pickering interfacial catalyst. Microporous Mesoporous Mater. **310**, 110677 (2021).

<https://doi.org/10.1016/j.micromeso.2020.110677>

[S5] G.C. Zheng, Z.W. Chen, K. Sentosun, I. Pérez-Juste, S. Bals et al., Shape control in ZIF-8 nanocrystals and metal nanoparticles@ZIF-8 heterostructures. Nanoscale **9**(43), 16645–16651 (2017).

<https://doi.org/10.1039/C7NR03739B>

[S6] X.Y. Lu, L. Kuai, F. Huang, J.S. Jiang, J.K. Song et al., Single-atom catalysts-based catalytic ROS clearance for efficient psoriasis treatment and relapse prevention via restoring ESR1. Nat. Commun. **14**(1), 6767 (2023).

<https://doi.org/10.1038/s41467-023-42477-y>

[S7] Y. Xiong, J. Dong, Z.Q. Huang, P. Xin, W. Chen et al., Single-atom Rh/N-doped carbon electrocatalyst for formic acid oxidation. Nat. Nanotechnol. **15**, 390–397 (2020).

https://doi.org/10.1038/s41565-020-0665-x

[S8] X. Xie, L. Peng, H. Yang, G.I.N. Waterhouse, L. Shang et al., MIL-101-derived mesoporous carbon supporting highly exposed Fe single-atom sites as efficient oxygen reduction reaction catalysts. Adv. Mater. **33**, 2101038 (2021).

https://doi.org/10.1002/adma.202101038

[S9] K. Ghosh, J. Ghosh, P.K. Giri, Accordion-like multilayered two-dimensional Ti_3_C_2_T_x_ MXenes for catalytic elimination of organic dyes from wastewater the fenton reaction. ACS Appl. Nano Mater. **5**(11), 16451–16461 (2022).

<https://doi.org/10.1021/acsanm.2c03511>

[S10] G.H. Hu, J. Kang, L.W.T. Ng, X.X. Zhu, R.C.T. Howe et al., Functional inks and printing of two-dimensional materials. Chem. Soc. Rev. **47**(9), 3265–3300 (2018).

<https://doi.org/10.1039/C8CS00084K>

[S11] L.J. Deiner, T.L. Reitz, Inkjet and aerosol jet printing of electrochemical devices for energy conversion and storage. Adv. Eng. Mater. **19**(7), 1600878 (2017).

<https://doi.org/10.1002/adem.201600878>

[S12] Y.K. Lee, J. Kim, Y. Kim, J.W. Kwak, Y. Yoon et al., Room temperature electrochemical sintering of Zn microparticles and its use in printable conducting inks for bioresorbable electronics. Adv. Mater. **29**(38), 1702665 (2017).

<https://doi.org/10.1002/adma.201702665>

[S13] Y.Z. Shao, L.S. Wei, X.Y. Wu, C.M. Jiang, Y. Yao et al., Room-temperature high-precision printing of flexible wireless electronics based on MXene inks. Nat. Commun. **13**(1), 3223 (2022).

<https://doi.org/10.1038/s41467-022-30648-2>

[S14] P.A. Lopes, D.F. Fernandes, A.F. Silva, D.G. Marques, A.T. de Almeida et al., Bi-phasic Ag-in-Ga-embedded elastomer inks for digitally printed, ultra-stretchable, multi-layer electronics. ACS Appl. Mater. Interfaces **13**(12), 14565–14574 (2021).

<https://doi.org/10.1021/acsami.0c22206>

[S15] N. Matsuhisa, M. Kaltenbrunner, T. Yokota, H. Jinno, K. Kuribara et al., Printable elastic conductors with a high conductivity for electronic textile applications. Nat. Commun. **6**, 7461 (2015).

<https://doi.org/10.1038/ncomms8461>

[S16] Z. Li, T.R. Le, Z. Wu, Y.G. Yao, L.Y. Li et al., Rational design of a printable, highly conductive silicone-based electrically conductive adhesive for stretchable radio-frequency antennas. Adv. Funct. Mater. **25**(3), 464–470 (2015).

<https://doi.org/10.1002/adfm.201403275>

[S17] X.W. Tang, G. Murali, H. Lee, S. Park, S. Lee et al., Engineering aggregation-resistant MXene nanosheets as highly conductive and stable inks for all-printed electronics. Adv. Funct. Mater. **31**(29), 2010897 (2021).

<https://doi.org/10.1002/adfm.202010897>

[S18] D. Wen, X. Wang, L. Liu, C. Hu, C. Sun et al., Inkjet printing transparent and conductive MXene (Ti_3_C_2_T_x_) films: a strategy for flexible energy storage devices. ACS Appl. Mater. Interfaces **13**(15), 17766–17780 (2021).

<https://doi.org/10.1021/acsami.1c00724>

[S19] C.F. Zhang, L. McKeon, M.P. Kremer, S.H. Park, O. Ronan et al., Additive-free MXene inks and direct printing of micro-supercapacitors. Nat. Commun. **10**, 1795 (2019).

<https://doi.org/10.1038/s41467-019-09398-1>

[S20] M. Vural, A. Pena-Francesch, J. Bars-Pomes, H. Jung, H. Gudapati et al., Inkjet printing of self-assembled 2D titanium carbide and protein electrodes for stimuli-responsive electromagnetic shielding. Adv. Funct. Mater. **28**(32), 1801972 (2018).

<https://doi.org/10.1002/adfm.201801972>

[S21] M. Aakyiir, B. Tanner, P.L. Yap, H. Rastin, T.T. Tung et al., 3D printing interface-modified PDMS/MXene nanocomposites for stretchable conductors. J. Mater. Sci. Technol. **117**, 174–182 (2022).

<https://doi.org/10.1016/j.jmst.2021.11.048>

[S22] V. Dua, S.P. Surwade, S. Ammu, S.R. Agnihotra, S. Jain et al., All-organic vapor sensor using inkjet-printed reduced graphene oxide. Angew. Chem. Int. Ed. **49**(12), 2154–2157 (2010).

<https://doi.org/10.1002/ange.200905089>

[S23] Q. Liu, B.B. Zhang, Q. Xu, Y.Y. Hou, S. Seyedin et al., Development of graphene oxide/polyaniline inks for high performance flexible microsupercapacitors via extrusion printing. Adv. Funct. Mater. **28**(21), 1706592 (2018).

<https://doi.org/10.1002/adfm.201706592>

[S24] K. Parvez, R. Worsley, A. Alieva, A. Felten, C. Casiraghi, Water-based and inkjet printable inks made by electrochemically exfoliated graphene. Carbon **149**, 213–221 (2019).

<https://doi.org/10.1016/j.carbon.2019.04.047>

[S25] S. Majee, W. Zhao, A. Sugunan, T. Gillgren, J.A. Larsson et al., Highly conductive films by rapid photonic annealing of inkjet printable starch-graphene ink. Adv. Mater. Interfaces **9**(5), 2101884 (2022).

<https://doi.org/10.1002/admi.202101884>

[S26] E.B. Secor, B.Y. Ahn, T.Z. Gao, J.A. Lewis, M.C. Hersam, Rapid and versatile photonic annealing of graphene inks for flexible printed electronics. Adv. Mater. **27**(42), 6683–6688 (2015).

<https://doi.org/10.1002/adma.201502866>

[S27] U. Kraft, F. Molina-Lopez, D. Son, Z.A. Bao, B. Murmann, Ink development and printing of conducting polymers for intrinsically stretchable interconnects and circuits. Adv. Electron. Mater. **6**(1), 1900681 (2020).

<https://doi.org/10.1002/aelm.201900681>

[S28] B. Schmatz, A.W. Lang, J.R. Reynolds, Fully printed organic electrochemical transistors from green solvents. Adv. Funct. Mater. **29**(44), 1905266 (2019).

<https://doi.org/10.1002/adfm.201905266>

[S29] Z.M. Deng, L.L. Li, P.P. Tang, C.Y. Jiao, Z.Z. Yu et al., Controllable surface-grafted MXene inks for electromagnetic wave modulation and infrared anti-counterfeiting applications. ACS Nano **16**(10), 16976–16986 (2022).

<https://doi.org/10.1021/acsnano.2c07084>

[S30] M.S. Nam, J.Y. Kim, A. Mirzaei, M. H. Lee, H. W. Kim et al., Au- and Pt-decorated Ti_3_C_2_T_x_ MXenes for preparing self-heated and flexible NH_3_ gas sensors. Sensors and Actuators B: Chemical **403**, 135112 (2024).

<https://doi.org/10.1016/j.snb.2023.135112>

[S31] S.H. Lee, W. Eom, H. Shin, R.B. Ambade, J.H. Bang et al., Room-temperature, highly durable Ti_3_C_2_T_x_ MXene/graphene hybrid fibers for NH_3_ gas sensing. ACS Appl. Mater. Interfaces **12**(9), 10434–10442 (2020).

<https://doi.org/10.1021/acsami.9b21765>

[S32] H.L. Tai, Z.H. Duan, Z.Z. He, X. Li, J.L. Xu et al., Enhanced ammonia response of Ti_3_C_2_T_x_ nanosheets supported by TiO_2_ nanoparticles at room temperature. Sensors and Actuators B: Chemical **298**, 126874 (2019).

<https://doi.org/10.1016/j.snb.2019.126874>

[S33] M. Wu, M. He, Q.K. Hu, Q.H. Wu, G. Sun et al., Ti_3_C_2_ MXene-based sensors with high selectivity for NH_3_ detection at room temperature. ACS Sens. **4**(10), 2763–2770 (2019).

<https://doi.org/10.1021/acssensors.9b01308>

[S34] H.M. Yu, L.H. Dai, Y.Q. Liu, Y. Zhou, P. Fan et al., Ti_3_C_2_T_x_ MXene-SnO_2_ nanocomposite for superior room temperature ammonia gas sensor. J. Alloys Compd. **962**, 171170 (2023).

<https://doi.org/10.1016/j.jallcom.2023.171170>
